# Importins promote high-frequency NF-κB oscillations increasing information channel capacity

**DOI:** 10.1186/s13062-016-0164-z

**Published:** 2016-11-11

**Authors:** Zbigniew Korwek, Karolina Tudelska, Paweł Nałęcz-Jawecki, Maciej Czerkies, Wiktor Prus, Joanna Markiewicz, Marek Kochańczyk, Tomasz Lipniacki

**Affiliations:** 1 Institute of Fundamental Technological Research, Polish Academy of Sciences, Warsaw, Poland; 2 College of Inter-Faculty Individual Studies in Mathematics and Natural Sciences, University of Warsaw, Warsaw, Poland

**Keywords:** Karyopherins, Nucleocytoplasmic transport, Negative feedback, Channel information capacity, Mathematical modelling

## Abstract

**Background:**

Importins and exportins influence gene expression by enabling nucleocytoplasmic shuttling of transcription factors. A key transcription factor of innate immunity, NF-κB, is sequestered in the cytoplasm by its inhibitor, IκBα, which masks nuclear localization sequence of NF-κB. In response to TNFα or LPS, IκBα is degraded, which allows importins to bind NF-κB and shepherd it across nuclear pores. NF-κB nuclear activity is terminated when newly synthesized IκBα enters the nucleus, binds NF-κB and exportin which directs the complex to the cytoplasm. Although importins/exportins are known to regulate spatiotemporal kinetics of NF-κB and other transcription factors governing innate immunity, the mechanistic details of these interactions have not been elucidated and mathematically modelled.

**Results:**

Based on our quantitative experimental data, we pursue NF-κB system modelling by explicitly including NF-κB–importin and IκBα–exportin binding to show that the competition between importins and IκBα enables NF-κB nuclear translocation despite high levels of IκBα. These interactions reduce the effective relaxation time and allow the NF-κB regulatory pathway to respond to recurrent TNFα pulses of 45-min period, which is about twice shorter than the characteristic period of NF-κB oscillations. By stochastic simulations of model dynamics we demonstrate that randomly appearing, short TNFα pulses can be converted to essentially digital pulses of NF-κB activity, provided that intervals between input pulses are not shorter than 1 h.

**Conclusions:**

By including interactions involving importin-α and exportin we bring the modelling of spatiotemporal kinetics of transcription factors to a more mechanistic level. Basing on the analysis of the pursued model we estimated the information transmission rate of the NF-κB pathway as 1 bit per hour.

**Reviewers:**

This article was reviewed by Marek Kimmel, James Faeder and William Hlavacek.

**Electronic supplementary material:**

The online version of this article (doi:10.1186/s13062-016-0164-z) contains supplementary material.

## Background

Control of nuclear localization of proteins,especially transcription factors (TFs), is a crucial aspect of gene expression regulation [1, 2]. While nuclear pore complexes (NPCs) allow passive diffusion of molecules of mass below approximately 40 kDa [3], larger molecules require active transport to cross the nuclear envelope. As a result, nuclear transport of most TFs is energy-dependent and in most cases involves homologous family of carrier molecules called karyopherins, with import carriers called importins and export carriers called exportins [4]. Importins recognize cargoes containing a signal peptide sequence called the nuclear localization signal (NLS). The signal peptide sequence recognized by exportins is called the nuclear export signal (NES). In the classical nuclear protein import pathway, the importin-α family functions as an NLS-recognizing adaptor, which is in turn recognized by importin-β (KPNB1), a carrier mediating interactions with NPC. In the best recognized pathway of nuclear protein export, exportin 1 (XPO1) functions both in NES recognition and as a carrier. Usually, cargoes possessing both NLS and NES sequences undergo continuous shuttling between the cytoplasmic and the nuclear compartment. Thus, localization of a TF can be dynamically regulated by its conformational change or association with other molecules affecting the accessibility of the NLS or NES for karyopherin binding [5–7].

The energy required for nuclear transport is supplied by a GTPase called Ran (RAN) and is used among others for importin-β dissociation. Following classical nuclear protein import, conversion of importin-bound RanGDP into RanGTP causes the release of importin-α:cargo complex from importin-β in the nucleus [3]. The mechanisms of importin-α release vary and their details are still debated. However, since the affinity of importin-α for its target classical NLS is ~10 nM, the release is likely to require catalysis or competitive binding [4]. GTPases are essential for active nuclear transport, but due to their abundance [8, 9], they do not limit the rates of nuclear transport processes. Instead, these rates can be limited by diffusion or active transport along microtubules [10], as the karyopherin:cargo complexes are formed anywhere in a cellular compartment, and need to translocate first into the vicinity of the nuclear envelope before translocation across a NPC can occur.

Here, we focus on NF-κB, an ubiquitous TF fundamental in innate immune response, which upon stimulation exhibits oscillatory nucleocytoplasmic shuttling. These oscillations result from the negative feedbacks mediated by its inhibitors, IκBα (NFKBIA) [11] and A20 (TNFAIP3) [12], and require bidirectional transport across the nuclear membrane. The most abundant of NF-κB heterodimers consist of RelA (RELA) and p50 (NFKB1) subunits, and in resting cells most of them are retained in the cytoplasm by IκBα which masks the NLS of RelA [13, 14]. Upon TNFα or LPS stimulation, IκBα is phosphorylated by kinase complex IKK, and then ubiquitinated and degraded by the 26S proteasome [15–18]. IκBα degradation exposes the NLS of RelA, allowing importin-α3 (KPNA3) or α4 (KPNA4) binding [19, 20]. Then, the NF-κB:importin-α complex can be intercepted by importin-β, which interacts with the nuclear pore to effect NF-κB translocation [21]. In the nucleus, NF-κB triggers the expression of numerous target genes, including two of its inhibitors, A20 and IκBα [22]. A20 attenuates IKK activity [23], allowing for accumulation of newly synthesized IκBα, which diffuses into the nucleus and binds NF-κB. The transcriptionally active NF-κB is removed from gene promoters by IκBα binding, which terminates transcription. After exportin 1 recognizes the NES of IκBα, it enables free IκBα as well as the IκBα:NF-κB complexes to pass through the NPC and leave the nucleus [24–27]. In this way, exportins participate in the suppression of NF-κB signalling.

Despite the fact that modelling of nuclear trafficking has been considered in the past [28, 29] and NF-κB signalling network has been extensively studied both experimentally and by mathematical modelling [30–39], the regulation of NF-κB translocation by karyopherins has not been modelled explicitly. Following the work of Hoffmann et al. [30], existing computational models have simplified these interactions by assuming that free NF-κB translocates to the nucleus, while NF-κB:IκBα complexes translocate to the cytoplasm. We found that this approach falls short of capturing the spatiotemporal coevolution of NF-κB and IκBα levels during the second NF-κB pulse. We observe, both at the population and single-cell level, that NF-κB enters the nucleus despite the excess of IκBα. This suggests that a fraction of released NF-κB is rapidly captured by importin-α and in this way escapes from binding by the remaining IκBα. Therefore, we pursued NF-κB modelling towards a more detailed mechanistic description, which better explains the observed spatiotemporal coevolution of levels of NF-κB and its inhibitor IκBα in response to stimulation with TNFα or LPS. The proposed model captures the puzzling short-period NF-κB oscillations in response to pulsed TNFα stimulation observed recently by Zambrano et al. [40]. As the NF-κB–IκBα feedback loop emerges as the canonical example of regulation of transcription factor signalling [11], its mechanistic modelling can add to understanding of spatiotemporal kinetics of other transcription factors [41, 42].

Cheong et al. [43] and Selimkhanov et al. [44] demonstrated that the NF-κB pathway transmits only 1 bit of information about the level of TNFα, which is equivalent to resolving whether TNFα is present or not. The interesting question is how frequently this bit of information can be transmitted. The ability to respond to frequent pulses is controlled by the refractory time, which may depend on the specific cytokine or other stimuli, and as found by Adamson et al. [45] can be shorter when one type of the stimuli is replaced by another. Based on the proposed model, we theoretically demonstrate that the NF-κB pathway can transmit information about the short TNFα pulses as long as their frequency does not exceed 1 per hour. Since TNFα is short lived in vivo with half-time of order of 10 min [18, 46] we expect that information is encoded in the sequence of TNFα pulses rather than in their amplitude.

## Results

### Spatiotemporal profiles of NF-κB and IκBα in response to TNFα and LPS

We investigated the spatiotemporal NF-κB–IκBα relationship using immunofluorescent staining. We chose this technique in addition to Western blotting (WB) to obtain information about the levels and localization of NF-κB and IκBα in single cells. Fluorescent tagging of NF-κB and IκBα [47], although a method of choice for examining NF-κB regulation in single cells, could influence protein interactions with karyopherins and, by increasing IκBα mass above 40 kDa, suppress its passive diffusion through nuclear pores.

**Fig. 1:**
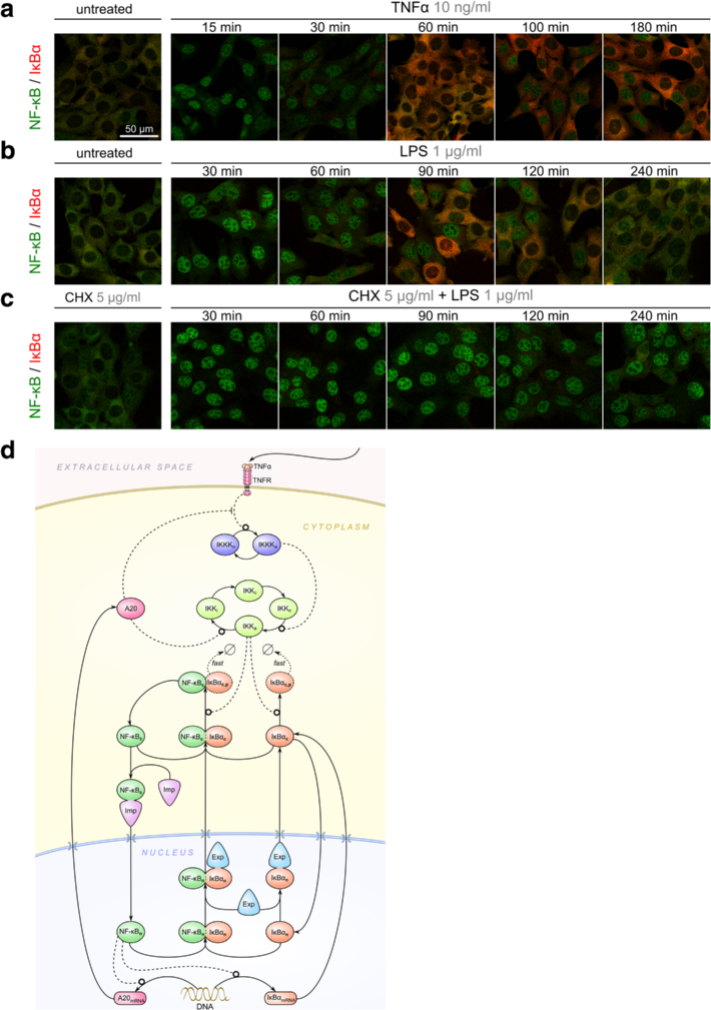
Spatiotemporal profiles of NF-κB and IκBα in response to TNFα and LPS and the emerging model. Immunostaining confocal images showing localization and level of RelA (component of NF-κB dimer) and IκBα after (**a**) TNFα stimulation, (**b**) LPS stimulation, or (**c**) LPS + CHX costimulation. In (**c**), CHX was added 1 h before LPS stimulation. Additional file 4, Additional file 5 and Additional file 6 provide full confocal images corresponding to the images shown in (**a**–**c**). Additional file 7 provides confocal images of cells stimulated with 10 ng/ml TNFα for short time points (0, 5, 10, 15, 20 and 30 min). **d** Model scheme. Arrow-headed lines denote transitions, mRNA or protein synthesis, complex formation, or fast degradation; circle-headed lines denote positive influence; hammer-headed line denotes negative influence. Importins direct NF-κB to the nucleus, whereas exportins bind IκBα and IκBα–NF-κB dimers and shepherd them to the cytoplasm. All other nucleocytoplasmic translocations are assumed to proceed in a karyopherin-independent manner

As shown in Fig. 1a and b, in unstimulated cells most of NF-κB is sequestered by IκBα in the cytoplasm. TNFα- or LPS-induced IκBα degradation observed at 15 and 30 min after stimulation results in nuclear NF-κB translocation at 15–30 min for TNFα and 30–60 min for LPS stimulation. Immunostaining images in Fig. 1a show that upon TNFα stimulation NF-κB returns to cytoplasm at 60 min and then translocates to the nucleus again at 100 min despite accumulation of IκBα above its baseline level. Interpretation of responses to LPS is more difficult due to a delayed and more heterogeneous cell activation [48]. Nevertheless, also in this case immunostaining images indicate that at 90 and 120 min a fraction of cells have increased level of cytoplasmic IκBα and simultaneously a sizable nuclear NF-κB translocation. In response to LPS costimulation with a protein synthesis inhibitor, cycloheximide (CHX), IκBα is degraded but not resynthesized, allowing NF-κB to remain in nucleus for 4 h (Fig. 1c).

### Formulation of the computational model

We propose that the nuclear translocation of NF-κB occurring after 90 min of TNFα stimulation despite the excess of IκBα is enabled by NF-κB–importin interactions, which prevent the newly released NF-κB from binding to remaining IκBα molecules. In order to investigate this hypothesis we pursued our previous model [33, 49] and explicitly considered NF-κB–importin binding in the cytoplasm and IκBα–exportin binding in the nucleus. The emerging model is outlined in Fig. 1d. IκBα kinase complex (IKK) is activated via kinase IKKK in response to TNFα (or LPS). Active IKK (IKK_a_) phosphorylates free as well as NF-κB-bound IκBα (at Ser 32 and 36) leading to its polyubiquitination and rapid degradation by the 26S proteasome [15, 18]. When IκBα is in excess, the newly released NF-κB can either bind another IκBα molecule or translocate to the nucleus. Since nuclear translocation of NF-κB is preceded by binding of importins to its NLS sequences, we propose that competition for NF-κB binding between importin-α and IκBα determines whether released NF-κB enters the nucleus or becomes sequestered in the cytoplasm by another IκBα molecule. Although previous models assumed that free NF-κB may translocate to the nucleus, importin-α binding precluding sequestration by IκBα has not been explicitly considered.

NF-κB translocates back to the cytoplasm complexed with IκBα, which may pass through nuclear pores after association with exportin 1. To reduce complexity of the model we assume that both importin-α and exportin 1 dissociate from their cargo immediately after crossing the nuclear pore. Later we will compare it with a model variant in which the dissociation step is considered explicitly. IκBα, a small 32 kDa protein, can translocate to the nucleus independently of importin-α, so for simplicity we do not assume any IκBα–importin-α interactions. Finally, because karyopherins have multiple binding partners apart from IκBα and NF-κB, we assume that they are present in excess to IκBα and NF-κB and that their levels are not influenced by IκBα and NF-κB binding.

The rates of karyopherin binding and of translocation of cargo-karyopherin complexes are set based on the following assumptions. Karyopherin-bound proteins can freely but unidirectionally move across the nuclear membrane. For freely diffusing molecules the ratio of nuclear export rate to nuclear import rate is equal to the ratio of cytoplasmic-to-nuclear volume, k_v_, which ensures cell-uniform concentration in equilibrium. Hence, we assume that the ratio of karyopherin-dependent nuclear export to import rate is equal k_v_. We assume that the effective NF-κB:importin-α binding rate is higher than NF-κB:IκBα binding rate. Under this assumption, NF-κB can translocate to the nucleus even when IκBα is temporarily in excess (Fig. 1a and b). In contrast, we assume that IκBα:exportin 1 binding rate is lower than that of NF-κB and IκBα, which in turn allows nuclear IκBα to bind NF-κB before it is transported back to the cytoplasm. Overall, the effective IκBα nuclear export rate is higher than its import rate, which reflects the observation that IκBα, even if present in excess to NF-κB, localizes mainly in the cytoplasm.

The structure of our model, except for the interactions of IκBα and NF-κB with karyopherins, is laid out as in our previous paper [33] (see Additional file 1 for a complete model definition and parameters). However, following our later study [35], we consider all reactions stochastic, firing with concentration-dependent propensities. We used the model-specification language of BioNetGen (BNGL) to define types of molecules included in our model and to specify rules of interactions. The conventions of BNGL are described in detail elsewhere [50, 51]. BioNetGen allows for efficient deterministic and stochastic simulation employing a variation of Gillespie’s direct method [52]. A BioNetGen language-encoded model is enclosed in Additional file 2.

**Fig. 2:**
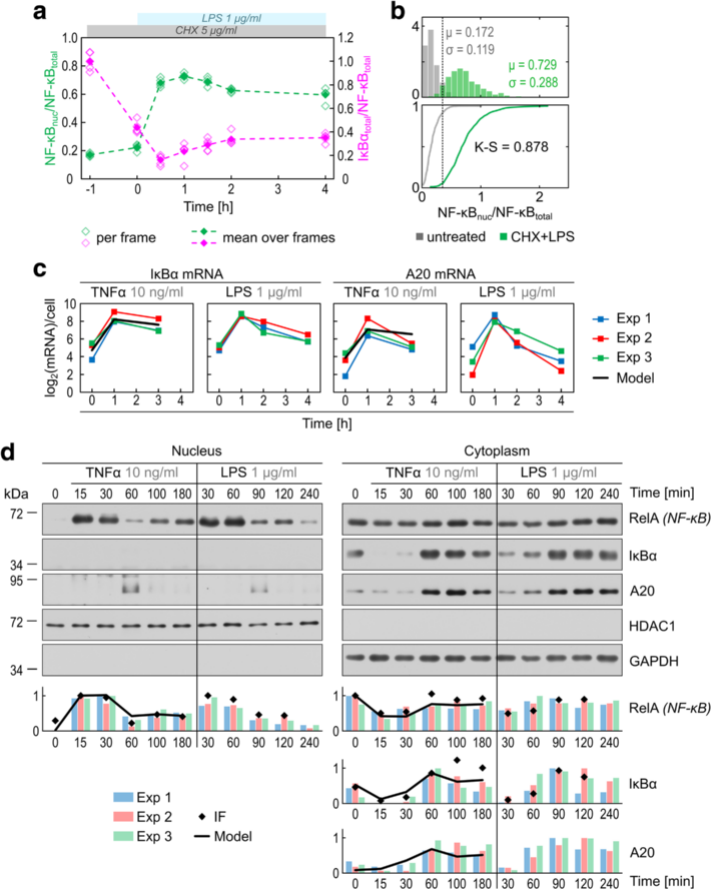
Quantification of NF-κB, IκBα and A20 levels in response to TNFα and LPS stimulation. **a** Immunostaining time profiles of RelA (NF-κB) nuclear/total ratio and total IκBα/total RelA (NF-κB) ratio (in arbitrary units) in response to 1 μg/ml LPS with 5 μg/ml CHX costimulation started 1 h before LPS. Open squares show values calculated in each of five confocal frames analysed. Filled squares show mean over these five frames containing in total more than 500 cells for each time point. **b** Histograms showing nuclear RelA (NF-κB) fluorescence normalized to cell average fluorescence for unstimulated cells (*grey*) and 1 h after 1 μg/ml LPS with 5 μg/ml CHX costimulation (*green*); see Methods for details of normalization. Coefficient μ is the histogram average while σ is the standard deviation. In stochastic numerical simulations, total single-cell NF-κB levels were drawn at random based on the data used to plot the histogram; see Methods. Bottom subpanel shows cumulative distributions for unstimulated (*grey line*) and stimulated (*green line*) cells. Kolmogorov–Smirnov statistic (K–S) equals 0.878, which implies that at least 87.8 % of cells respond to stimulation. **c** Experimental IκBα and A20 mRNA time profiles after 10 ng/ml TNFα and 1 μg/ml LPS stimulation from three independent measurements. Data show absolute quantification by digital PCR for TNFα stimulation or rescaled RT-PCR quantification using digital PCR measurements in selected time points. Model simulated mRNA profiles after 10 ng/ml TNFα show the average over 300 stochastic simulations. The numerical values are shown only for experimental time points, and are connected by line only to guide the eye. **d** Western blot analysis of cytoplasmic and nuclear fractions of RelA (NF-κB), IκBα and A20. Blots from one of three quantified experiments are shown. Nuclear IκBα and A20 were near the limit of detection. Model simulated protein profiles after 10 ng/ml TNFα show the average over 300 stochastic simulations

As described previously [33], the model accounts for two types of noise: intrinsic, associated with low numbers of molecules, and extrinsic, arising from initial heterogeneity of cells in the population. The major source of intrinsic noise is A20 and IκBα genes switching between on and off states (saying that a gene is on we state that the transcription factor is bound to DNA and all other conditions are satisfied for transcription to proceed). Extrinsic noise arises from variable expression of TNF receptors (TNFRs) and NF-κB. The receptor level variability results in heterogeneous cell sensitivity to the signal, whereas the NF-κB level variability is responsible for a broad distribution of NF-κB nuclear intensities observed even when almost whole NF-κB pool is translocated into the nucleus. Following our previous study [33], we assume a lognormal distribution of TNFR, but the distribution of NF-κB is estimated based on data from 1 h time-point from the CHX + LPS costimulation experiment (Figs. 1c and 2a–b). As shown in Fig. 2a, after 0.5 h of CHX + LPS costimulation about 90 % of IκBα is degraded, while NF-κB translocation reaches its maximum at 1 h with about 70 % of total NF-κB translocated to the nucleus. The distribution shown in Fig. 2b was obtained under a condition when the synthesis of NF-κB inhibitors is almost fully suppressed so it can be interpreted as the distribution of all of the NF-κB that has the potential to translocate to the nucleus upon LPS or TNFα stimulation. Therefore, based on the assessments by Carlotti et al. [53, 54], we assume that the median level of NF-κB is 10^5^ molecules per cell (with on average 30 % of NF-κB associated with inhibitors that are not degraded in response to LPS or TNFα) and we use this distribution to draw numbers of NF-κB molecules for stochastic simulations.

Based on the absolute quantification of IκBα and A20 transcripts (Fig. 2c) we modified IκBα and A20 mRNA synthesis and degradation coefficients, so that IκBα and A20 mRNA are transcribed at the rate of 0.2 mRNA/(s × gene copy) and translated at the rate of 0.5 protein/(s × mRNA). These two rates appear to be close to physiological maxima. The transcription speed was measured at ~60 nt/s [55] and minimal spacing between RNA polymerases as small as ~100 nt [56], which gives maximal transcription rate of about 0.6 mRNA/(s × gene copy). The rate of translation in eukaryotic cells has been estimated at 6 aa/s (or 18 nt/s) [57] and the ribosome centres can be only 40–60 nucleotides apart in 3D helical polysomal conformation [58], which implies the upper bound estimate of 0.5 protein/(s × mRNA). The assumed transcription rate assures that IκBα transcript level reaches about 300 mRNA molecules during the first NF-κB pulse, which together with the high translation rate allows for the rapid *de novo* synthesis of more than 10^5^ IκBα molecules needed to shepherd NF-κB back to the cytoplasm. Our estimates suggest that the IκBα–NF-κB negative loop is optimized to produce high-amplitude, short-lasting pulses of NF-κB activity.

Finally, with respect to the previous study, we reduce the coefficient of TNFα degradation to 10^-4^/s. TNFα is known to be short-lived in vivo, with reported half-life times ranging from 4.6 to 10.5 min [59–61]. However, these values represent the rate of elimination of TNFα from murine plasma/bloodstream resulting from many different processes occurring simultaneously in a complex system of the animal organism. Aside from internalisation by target stimulated cells, TNFα is mainly cleared from blood by liver and kidneys and is also broken down by plasma proteases (such as neutrophil elastase and cathepsin G – see [62] and references therein), resulting in short in vivo half-life time. These processes are either absent (blood filtration) or severely limited in vitro (protease degradation). Zambrano et al. [40] reported negligible degradation of TNFα used for stimulation of medium-cultured MEFs in a microfluidic chamber. Our own data indicate slow degradation (or activity loss) of TNFα incubated with cells. In an experiment reported in Additional file 3: Figure S1, we stimulated naive cells with media extracted after 6 h following stimulation of naive cells with TNFα at the initial concentration of 10 ng/ml. We observed a somewhat weaker and more heterogeneous response, resembling the response to 1 ng/ml dose, which allowed us to estimate the effective TNFα degradation/loss rate for our experimental conditions as 10^−4^/s. For such degradation rate, 10 ng/ml is degraded in 6 h to 10 ng/ml × e^−2.16^ ≈ 1.15 ng/ml. As we discussed previously [33], in small microfluidic chambers at low TNFα concentrations, effective TNFα loss rate can be much higher due to binding by more abundant TNFR and endocytosis. We observed also, based on ELISA, negligible degradation of TNFα in cell-free medium during the course of 24 h incubation at 22 °C and 37 °C. Taken together, these results suggest that cellular internalization is the main process in decreasing TNFα levels in the described in vitro conditions.

Accumulation of IκBα above initial level shown in Fig. 1a and 1b is corroborated by population data obtained by WB, which was quantified and used to fit model parameters, and juxtaposed with simulated trajectories (Fig. 2d). WB analysis indicate that in unstimulated cells the nuclear NF-κB level is very low. The TNFα- and LPS-induced degradation of IκBα at 15 and 30 min results in nuclear NF-κB translocation. The decrease of cytoplasmic IκBα (at 15 and 30 min for TNFα and 30 min for LPS) is more pronounced than the decrease of cytoplasmic NF-κB, which suggests that a fraction of NF-κB is sequestered by other inhibitors, such as IκBε or IκBβ, which are not degraded as rapidly as IκBα [30]. These additional NF-κB inhibitors are implicitly modelled by assuming that on average 30 % of NF-κB is associated with inhibitors that are not degraded in response to TNFα.

**Fig. 3:**
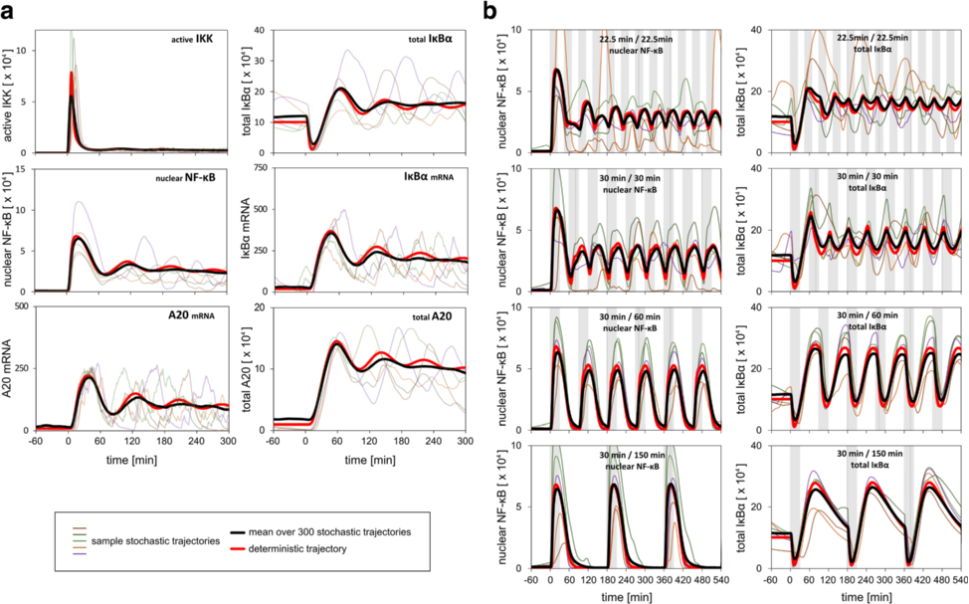
Numerical simulations. **a** Time profiles of active IKK (IKK_a_), total IκBα, nuclear NF-κB, IκBα mRNA, A20 mRNA, and total A20 in response to 10 ng/ml TNFα stimulation. Bold red line denotes deterministic stimulation, bold black line denotes average over 300 stochastic simulations, 5 thin colour lines show single cell stochastic simulations. TNFα stimulation starts at time = 0 and lasts till 300 min. **b** Stochastic (*thin colour lines*), deterministic (*bold red line*) and population average (bold black line) time profiles of total IκBα and nuclear NF-κB in response to pulsed 10 ng/ml TNFα stimulation. The simulation protocols correspond to repeated TNFα pulses in experiment performed by Zambrano et al. [40]: 22.5-min TNFα stimulation, 22.5-min break; 30-min TNFα stimulation, 30-min break; 30-min TNFα stimulation, 60-min break; 30-min TNFα stimulation, 150-min break

Figure 3 illustrates how the signal propagates from TNFR to NF-κB and through negative feedback loops. In short, TNFR activation leads to a pulse of IKK activity followed by an oscillating tail. Active IKK (IKK_a_) phosphorylates IκBα, leading to its rapid degradation, which allows NF-κB to translocate to the nucleus and trigger transcription of its inhibitors, IκBα and A20. Resythesized IκBα enters the nucleus, binds NF-κB and exportin 1, and the complex translocates back to the cytoplasm. A20 blocks TNFR activity and enhances transformation of IKK_a_ to inactive IKK_i_. The first pulse of nuclear NF-κB is followed by subsequent less pronounced pulses, which occur despite the accumulation of IκBα above its initial levels. Single-cell trajectories show progressing desynchronization of responses initially synchronized by TNFα stimulation. Due to the loss of cell synchronization, oscillations of the population average are more dampened than oscillations of individual cell trajectories. In the numerical simulations, cells were equilibrated in the absence of TNFα for 100 h. As shown in Additional file 3: Figure S2, unstimulated cells exhibit irregular oscillations, arising from spontaneous degradation of IκBα and resulting in low-level bursts of NF-κB activity.

In order to verify whether the modified model is capable of reproducing previously reported key experiments posing constraints on pathway connectivity and parameters, we performed a series of numerical simulations (Fig. 3b and Additional file 3: Figures S3, S4 and S5). In Fig. 3b we show stochastic, deterministic and population average time profiles of IκBα and nuclear NF-κB in response to pulsed 10 ng/ml TNFα stimulation. These protocols correspond to the recent experiment by Zambrano et al. [40], who observed NF-κB pulses in response to periodic TNFα stimulation with periods of 45, 60, 90, 180 min. The simulations show that the system can respond by NF-κB translocations even to the shortest-period pulses, and that during these pulses IκBα remains above the level expected for resting cells. The 45-min pulsing period is about half of the intrinsic oscillation period of 90–100 min [35, 63], and this intrinsic oscillation frequency becomes increasingly more visible when signal propagates downstream of IKK (Additional file 3: Figure S6). In (Additional file 3: Figure S3) we reproduce the responses of A20-deficient fibroblasts, observed by Lee et al. [12]. The stable, switch-like NF-κB activation in this knock-out cell line results from the lack of the A20-mediated negative feedback [31]. In Additional file 1: Figure S4 we reproduce responses to 1, 2, 5, 15, 45 min 10 ng/ml TNFα pulses, known to result in a single NF-κB translocation [30, 64] of amplitude weakly dependent on TNFα pulse duration. In Additional file 3: Figure S5 we reproduce responses to three series of 5-min TNFα pulses separated by time intervals of 60, 100 or 200 min from the experiment by Ashall et al. [34]. They observed that the NF-κB translocation amplitude of the second and third pulse is equal to that of the first pulse for time intervals of 200 min, and reduced to about 30 % for time intervals of 60 and 100 min.

### Model validation

Quantification of time series of confocal immunofluorescent images was performed to validate the proposed model. With the use of our in-house software, DAPI-stained nuclei were detected automatically (with occasional manual correction) and nuclear fluorescence was quantified in single cells. To obtain accurate single-cell cytoplasmic fluorescence, cytoplasmic contours were marked manually. Automatic quantification (see Methods for details of confocal images quantification) allowed us to calculate frame-average ratios such as: nuclear NF-κB/total NF-κB, total IκBα/total NF-κB. The last ratio can be only expressed in arbitrary units, since the actual values depend on staining protocols and laser intensities (kept low and the same in all experiments). Based on manual identification of cells, we calculate these values in selected representative cells (see Additional file 4 and Additional file 5 for confocal images with marked cells used for quantification).

**Fig. 4:**
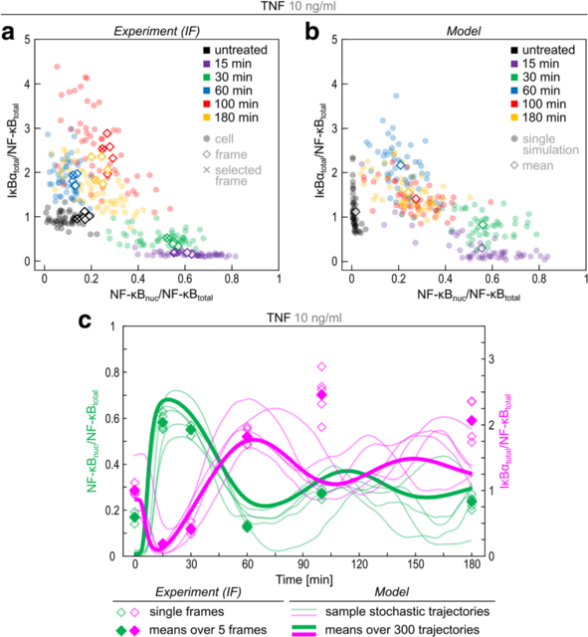
Model validation. **a**, **b** Scatter plots showing evolution of total IκBα/total (NF-κB) ratio and nuclear NF-κB/total NF-κB in response to 10 ng/ml TNFα. **a** Experiment-based scatter plots are based on quantified confocal images shown in Additional file 4. For each time point, boundaries of 50 stained cells and their nuclei were manually determined and IκBα and NF-κB were quantified. Dots represent single cells, squares represent averages over confocal images, crosses represent confocal images (see Additional file 4) from which single cells were analysed. **b** Simulation-based scatter plots were obtained in stochastic model simulations. **c** Model simulated time profiles of total IκBα/total NF-κB and nuclear NF-κB/total versus experimental data. Black bold line shows average over 300 stochastic simulations, colour lines show single-cell stochastic simulations. Open squares represent average over confocal images, filled squares represent mean over 5 frames

In Fig. 4a (experiment) and 4b (model) we provides scatter plots showing the coevolution of two observables: IκBα/NF-κB ratio in the cytoplasm and NF-κB nuclear fraction. At 15 and 30 min after TNFα stimulation we observe a decrease of cytoplasmic IκBα/NF-κB ratio and simultaneous increase of NF-κB nuclear fraction. In the experiment, between 30 and 60 min cytoplasmic IκBα increases above initial levels, and nuclear NF-κB drops to nearly initial levels. Then, despite the continuous rise of cytoplasmic IκBα/NF-κB ratio, nuclear NF-κB fraction increases. We should notice that although upraised IκBα level (with respect to the initial value) is observed both in WB and immunostaining images, the increase of IκBα level between 60 and 100 min is not observed in WB (Fig. 2d). Upraised IκBα level observed between 100 and 180 min confirms that the excess of cytoplasmic IκBα does not prevent the next pulse of NF-κB activity. Similar behaviour is also observed in the case of LPS stimulation (Additional file 3: Figure S5), but identification of the second pulse is difficult due to greater cell heterogeneity. Figure 4c, showing coevolution of total IκBα/NF-κB ratio and NF-κB nuclear fraction, confirms that at the second pulse the total amount of IκBα also exceeds that of NF-κB.

### Importins and information transmission through the NF-κB pathway

Negative feedbacks mediated by IκBα and A20 allow the system to be reset rapidly, however combined with noise and ultrasensitivity they reduce the information that can be transmitted by a single NF-κB pulse. By theoretical system modelling we found that NF-κB system exhibits stochastic robustness, allowing cells to respond differently to the same stimuli, but causing their individual responses to be unequivocal, essentially of all-or-nothing character [49]. It was confirmed by observation that the expression level of early genes, when calculated per responding cell, is independent of the TNFα dose [33]. Cheong et al. [43] and Selimkhanov et al. [44] demonstrated in a more rigorous way that the NF-κB pathway transmits only *n* ≈ 1 bit of information about the level of TNFα, which is equivalent to resolving whether TNFα is present or not.

According to the Shannon's definition [65] the information channel capacity can be expressed as

$$ C=\underset{T\to \infty }{ \lim}\left(\frac{{ \log}_2M}{T}\right), $$

where *M* is the number of different signal functions that can be reliably distinguished in time *T*. Thus *C* can be estimated as [65]

$$ C=f{ \log}_2{M}_0, $$

where *f* is frequency and *M*_0_ is a number of states that can be distinguished. Since the number of distinct states is 2, *C* is equal to the maximal frequency.

**Fig. 5:**
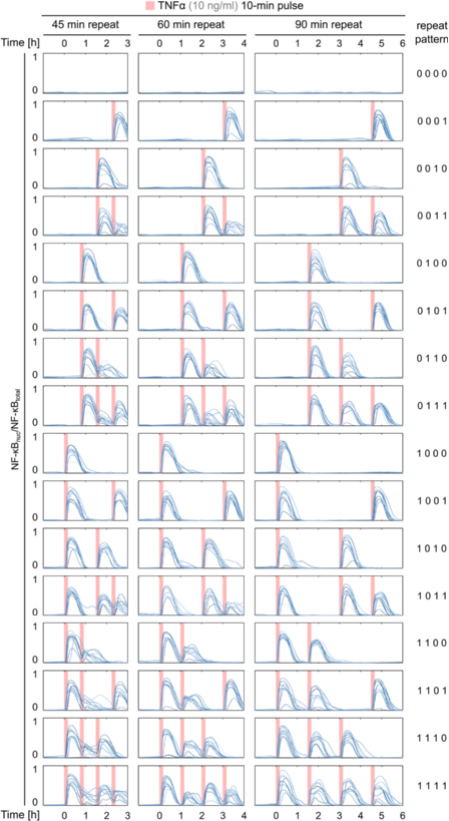
Transmission of information. NF-κB responses to 10 min 10 ng/ml TNFα pulses, that occur (or not) at the beginnings of subsequent 4 time intervals of length *T*, equal respectively 45 min (first column), 60 min (second column), 90 min (third column). Each of 16 sequences of 4 “true” or “false” TNFα pulses carry 4 bits of information. Simulations show that this information can be reliably transmitted for *T* ≥ 60 min, i.e., each “true” TNFα pulse is visible in the NF-κB nuclear fraction for almost all cells, while “false” pulses do not induce any response

In Fig. 5 we produce simulated nuclear NF-κB trajectories in response to series of four “true”-or-“false” TNFα pulses occurring in time intervals *T* of 45, 60 or 90 min. The simulations indicate that when *T* ≥ 60 min, in most cells NF-κB translocates in response to the “true” pulses and does not translocate in response to “false” pulses. This shows the system is capable of transmitting 2^4^ different signal functions in 4 h, which from definition allows to estimate *C* = 1 bit/h. The system’s ability to respond to pulses randomly placed in time reflects the lack of memory as postulated by Zambrano et al. [40].

**Fig. 6:**
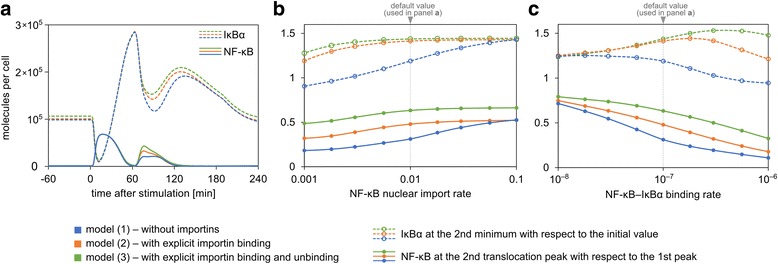
Analysis of three alternative models. Model without explicit implementation of importins (*blue*), model considering NF-κB–importin binding (analysed throughout this paper - *orange*) and a more detailed model in which the process of importin dissociation from NF-κB after translocation is not immediate, but has a finite rate 0.003/s (*green*). A protocol with two 10-min pulses of TNFα (10 ng/ml) at 60-min interval is analysed. **a** Deterministic trajectories of nuclear NF-κB and total IκBα for each model. **b**, **c** Parameter analysis: height of the second NF-κB translocation peak (as a fraction of the first peak height - *solid line*) and IκBα second minimum (normalised to concentration before stimulation - *dashed line*) are plotted against the varying NF-κB import rate (**b**) and NF-κB–IκBα binding rate (**c**). Other parameters are set to default values

The ability of the system to respond to high-frequency pulses follows from high rates of synthesis of inhibitors, molecular stripping, i.e., the process in which IκBα abruptly terminates transcription by actively removing NF-κB from gene promoters [66], and fast circulation of IκBα and NF-κB between cell compartments. The latter is enabled by importins and exportins. In Fig. 6 we show that explicit inclusion of importins allows the model to better reproduce high-frequency TNFα pulses. As shown in Fig. 5, responding to the second TNFα pulse is critical for the system’s ability to transmit high-frequency signals, therefore we analyse NF-κB and IκBα time profiles in response to two 10 min-long TNFα pulses at 1 h interval. We compare three models: (1) a model without importins, in which free NF-κB translocates to the nucleus, (2) the proposed model with importins, (3) a more detailed model with importins, which includes an additional step of NF-κB:importin-α dissociation in nucleus at the rate of 0.003/s. In Fig. 6a we show that the second peak of nuclear NF-κB is lowest in the model without importins, and highest in the more detailed model with importins. Importantly, it is also the detailed model where IκBα remains at the highest level during the second NF-κB translocation pulse.

Next, we compare the behaviour of these three model variants when two rate parameters are varied: NF-κB import rate (Fig. 6b) and NF-κB and IκBα binding rate (Fig. 6c). The rates are varied ten-fold below and ten-fold above their default values. In the whole range, model (3) shows the highest level of IκBα (at second minimum) and simultaneously the highest second-to-first peak amplitude ratio. Model (1) shows the lowest level of IκBα and simultaneously the lowest second-to-first peak amplitude ratio. In short, the more detailed the description, the higher the model ability to produce significant NF-κB translocations with a relatively small decrease in the level of IκBα. Unsurprisingly, when NF-κB translocation rate comes close to the assumed rate of NF-κB–importin binding (0.1/s), the dynamical role of this additional step becomes negligible and differences between models (1) and (2) vanish.

## Discussion

Karyopherins play a crucial role in the regulation of nucleocytoplasmic localization of transcription factors (of molecular mass exceeding 40 kDa), however there is no unique scheme of regulation. Two other transcription factors critical in innate immune responses, IRF3 and STAT1, are regulated in a different way. In unstimulated cells, IRF3 is mainly cytoplasmic but, possessing both a NLS and a NES, circulates between the cytoplasm and the nucleus. After infection, phosphorylated and dimerized IRF3 is captured by the nuclear CBP/p300 proteins [67]. In contrast, STAT1 localizes to the nucleus after phosphorylation on Tyr701, which makes its NLS available for binding by importin-α5 [68]. STAT1 after dephosphorylation may return to the cytoplasm, with the help of exportin 1, which recognizes its amino acid sequence located within the DNA-binding domain [69]. Lack of immediate inhibitors regulating translocation to the cytoplasm suggests that spatial dynamics of IRF3 and STAT1 is not pulsatile as in the case of NF-κB. These three transcription factors govern antiviral responses; NF-κB and IRF3 co-regulate transcription of IFNβ (IFNB1) [70] which via paracrine interactions triggers activation of STAT1 [71] and drives the cell into an antiviral state [72]. Deciphering complex interactions of innate immune responses would thus require specific mechanistic models of regulation of these three transcription factors.

NF-κB pathway is one of the best resolved regulatory systems of innate immunity. Since the seminal work by Hoffmann et al. [30] identifying IκBα-mediated negative feedback loop as responsible for oscillations, the NF-κB system was intensely modelled. We demonstrated [31] that the IκBα feedback loop functions only in the presence of A20, which mediates another negative feedback and attenuates IKK activity, protecting IκBα from rapid phosphorylation and degradation. These two loops control spatiotemporal activity of NF-κB, allowing its nucleocytoplasmic circulation observed at single-cell level even at constant TNFα stimulation [47]. As found by Nelson et al. [47], oscillations of NF-κB are indispensable for activation of NF-κB-responsive genes. Unsurprisingly, the pulsed TNFα stimulation leading to more pronounced pulses of NF-κB has received a lot of attention [34, 40, 63]. Kellogg and Tay [63] found that when TNFα pulsing frequency is close to the intrinsic frequency of NF-κB oscillations, 1/(90 min), or twice lower, NF-κB oscillations are well pronounced which increases NF-κB transcriptional efficiency. Zambrano et al. [40] found that high-amplitude TNFα oscillations can induce recurrent NF-κB translocations even when frequency of TNFα pulsing is twice higher than the intrinsic frequency of NF-κB oscillations. The authors proposed that the system has no memory, and oscillations are to segment time and provide “renewing opportunity windows for decision”.

Based on simultaneous measurements of IκBα and NF-κB levels in single cells, we found that NF-κB can translocate to the nucleus even when its immediate inhibitor, IκBα, is present in excess. To explain this observation, we propose that the NF-κB molecule released from the NF-κB:IκBα complex due to IκBα degradation can be rapidly bound by importin-α3/α4, which both protects it from binding by the remaining cytoplasmic IκBα molecules, and targets it for nuclear import. Only after remaining or newly synthesized IκBα translocates to the nucleus, can it bind NF-κB and exportin 1, which directs the complex back to the cytoplasm. By explicitly including importin-α and exportin 1 binding we pursued modelling of the NF-κB system towards a more mechanistic description. This allowed us to explain the observed nuclear translocations of NF-κB despite high IκBα levels, as well as the NF-κB translocation pulses observed by Zambrano et al. [40] in response to short-period TNFα stimulation. The model predicts that during these short NF-κB pulses IκBα is only partially degraded.

Absolute quantification of IκBα and A20 mRNA time profiles imposed constraints on kinetic model parameters. Based on the fitted parameters we conclude that IκBα/NF-κB feedback is optimized for fast response/fast inhibition. In response to 10 ng/ml TNFα, IκBα is almost fully degraded within 10–15 min, and restored at 60 min of TNFα stimulation to levels somewhat higher than initial. This can be achieved because IκBα and A20 transcription and translation rates are close to physiological maxima. Rapid synthesis of A20 is also important because it ensures attenuation of IKK activity, which in turn allows for accumulation of IκBα. Properties of the IκBα-controlled feedback loop, analysed recently by Fagerlund et al. [11], allow for rapid activation of NF-κB-responsive genes and nearly perfect adaptation to the signal at 60 min after TNFα stimulation. After 60 min of TNFα stimulation the A20/IκBα/NF-κB system returns to the proximity of the initial state, with somewhat increased levels of A20 and IκBα. However, as demonstrated here, in case of subsequent or continued TNFα stimulation, increased IκBα level does not preclude further NF-κB activation.

The importin/exportin system helps to reduce pathway repeating time *τ* to about 1 h and thus makes it possible to reach transmission frequency *f* = 1/h, which gives estimate of information channel capacity *C* = 1 bit/h. Because the IκBα transcription and translation rates are close to their physiological maxima, this short resetting time probably verges on the minimum for the systems based on degradation and resynthesis of the inhibitor protein. Combination of all-or-nothing type responses with fast resetting time suggests that the NF-κB system is optimized to digitize TNFα or LPS signals and convert them into pulses of transcription of NF-κB-responsive genes [33, 40].

## Conclusions

In summary, based on single-cell and population-wise quantification of mRNA and protein expression levels of IκBα and A20, as well as NF-κB translocation, we pursued NF-κB system modelling by including IκBα and NF-κB interactions with karyopherins. This novel model enables us to explain nuclear NF-κB translocations arising even when the level of IκBα is increased with respect to the level of unstimulated cells, as well as NF-κB translocations in response to high-frequency TNFα pulsing. By means of stochastic simulations we demonstrate that NF-κB pulses can be employed to transmit information at the rate of about 1 bit/h.

## Methods

### Cell lines and compounds

Experiments were performed on wild-type (WT) mouse embryonic fibroblasts (MEFs). The cells were cultured in Dulbecco’s Modified Eagle Medium (DMEM) with 4.5 g/l of D-glucose and 0.1 mM L-glutamine (ThermoFischer Scientific), supplemented with 10 % fetal bovine serum (ThermoFischer Scientific) and 100 mg/ml penicillin/streptomycin mix (Sigma-Aldrich). Cells were grown and maintained in a conditioned incubator at 37 °C, 5 % CO_2_. For stimulation, cells were seeded on dishes, multi-well plates or coverslips, depending on the type of experiment and allowed to adhere overnight at 37 °C. All cell lines were routinely tested against mycoplasma contamination by DAPI staining and PCR. Lipopolysaccharide (LPS) from Escherichia coli 0111:B4 (purified by ion-exchange chromatography) and mouse recombinant Tumor Necrosis Factor alpha (TNFα) were purchased from Sigma Aldrich. In order to disrupt LPS micelles, it was solubilised in a bath sonicator for 15 min and vortexed vigorously for additional 1 min prior to making further dilutions and adding to cells. Cycloheximide (Sigma-Aldrich) was administered to cells at a final concentration of 5 μg/ml 60 min before LPS.

### Immunostaining

Cells were seeded on a 12 mm-diameter round glass coverslips. Seeding density was 50,000 cells/coverslip. After stimulation, cells on coverslips were washed with PBS and immediately fixed with 4 % formaldehyde (20 min, room temperature). Cells were then washed thoroughly and incubated for 10 min with 50 mM NH4Cl in order to block reactive aldehyde groups left after fixation. Cell membranes were permeabilized with 0.1 % Triton X-100 (Sigma-Aldrich) for 5 min, washed again and blocked with 5 % BSA/PBS. Antibodies detecting target proteins, anti-p65 (D14E12, Cell Signalling Technologies) or anti-IκBα (L35A5, Cell Signalling Technologies) were then added to the cells in 5 % BSA/PBS, and incubated for 1.5 h. After washing with PBS, appropriate secondary antibodies conjugated with fluorescent dyes (Alexa 488/Alexa 555) were added and incubated for another 1.5 h. Subsequently, cells were washed and their nuclei were stained for 10 min with 200 ng/ml DAPI (Sigma-Aldrich). Coverslips were mounted on microscope slides with a drop of Mowiol (Sigma-Aldrich) and observed using Leica TCS SP5 X confocal microscope with Leica Application Suite AF software.

### Microscopic image analysis

Confocal images obtained from the immunostaining were segmented within our in-house software (MEFTrack). Automatically detected nuclear contours (based on DAPI nuclear staining) were corrected manually or excluded when the corresponding nuclei turned out to be on border-line or unfit (mitotic, overlapping or otherwise misshapen). For a limited number of cells (see Additional file 4 and Additional file 5) cell contours were marked manually. Analysis of these cells was used for producing Fig. 4a (dots) and Additional file 3: Figure S7 (dots). Fluorescence of each region enclosed by those nuclear or cellular contours was calculated as a sum of intensities of its pixels. The fluorescence of all regions were subject to analysis with auxiliary Matlab scripts, which eventually provided estimates of the magnitude of nuclear translocation and protein abundance. A correction for background noise was applied to fluorescence intensities in all contour-enclosed regions, in all relevant channels (green for NF-κB, red for IκBα, and DAPI for nuclear staining). In a given channel, for a given compartment the background-corrected fluorescence is denoted by *I*_compartment_^channel^, where superscript denotes channel (NF-κB, IκBα or DAPI), and subscript denotes compartment (c – cytoplasm, n – nucleus, cell – whole cell). Bold ***I*** denotes value averaged over all compartments of a given type within a frame.

#### Quantification of NF-κB nuclear fraction

1. For single-cell analysis (Fig. 4a and Additional file 3: Figure S7 – *dots*) in the case when both nuclear and cell contours are determined, nuclear NF-κB fraction in *i* ^th^ cell is calculated as

1 $$ \mathrm{raw}\_\mathrm{N}\mathrm{F}\hbox{-} {\upkappa \mathrm{B}}_{\mathrm{n}\mathrm{uc}}/\mathrm{N}\mathrm{F}\hbox{-} {\upkappa \mathrm{B}}_{\mathrm{total}}(i) = \frac{I_{{\mathrm{n}}_i}^{\mathrm{NF}\upkappa \mathrm{B}}}{I_{{\mathrm{cell}}_i}^{\mathrm{NF}\upkappa \mathrm{B}\ }}\ . $$

The “raw” values are further corrected for a residual fluorescence as described at the end of this subsection.

(2) For single-cell analysis (Fig. 2b – *histogram*) in the case when only nuclear contours are determined, nuclear NF-κB fraction in *i* ^th^ cell is calculated as

2 $$ \mathrm{raw}\_\mathrm{N}\mathrm{F}\hbox{-} {\upkappa \mathrm{B}}_{\mathrm{n}\mathrm{uc}}/\mathrm{N}\mathrm{F}\hbox{-} {\upkappa \mathrm{B}}_{\mathrm{total}}(i)=\frac{I_{{\mathrm{n}}_i}^{\mathrm{NF}\upkappa \mathrm{B}}}{{\boldsymbol{I}}_{\mathrm{cell}}^{\mathrm{NF}\upkappa \mathrm{B}}}\frac{{\boldsymbol{I}}_{\mathrm{cell}}^{\mathrm{DAPI}}}{I_{{\mathrm{cell}}_i}^{\mathrm{DAPI}}}, $$

where the average cell fluorescences in the NF-κB and DAPI channels, ***I***_cell_^NFκB^ and ***I***_cell_^DAPI^, are calculated by dividing total frame fluorescence (with background correction) in respective channel by the number of cells in the analyzed frame. The applied normalization using DAPI staining corrects possible errors resulting from out of focus cell displacements (intensity of displaced cells registers weakly in both the NF-κB and DAPI channels).

(3) For frame-wise average analysis (Figs. 2a and 4a and c – *diamonds*) nuclear NF-κB fraction is calculated as

3 $$ \mathrm{raw}\_\mathrm{N}\mathrm{F}\hbox{-} {\upkappa \mathrm{B}}_{\mathrm{n}\mathrm{uc}}/\mathrm{N}\mathrm{F}\hbox{-} {\upkappa \mathrm{B}}_{\mathrm{total}}\left(\mathrm{frame}\right)=\frac{{\boldsymbol{I}}_{\mathrm{n}}^{\mathrm{NF}\upkappa \mathrm{B}}}{{\boldsymbol{I}}_{\mathrm{cell}}^{\mathrm{NF}\upkappa \mathrm{B}}}, $$

where the average nuclear fluorescence in the NF-κB channel, ***I***_n_^NFκB^, is calculated by dividing the sum of nuclear fluorescence in NF-κB channel by the number of cells in the analyzed frame.

#### Correction for the residual fluorescence

Our Western blot data show almost no nuclear NF-κB in untreated cells, but the lowest ratio of NF-κB_nuc_ to NF-κB_total_ fluorescence that we observed in untreated cells is about *δ* = 0.1. We assume that this effect is due to the presence of cytoplasmic NF-κB above and below the nucleus, and that this residual fluorescence registers as nuclear. Thus we correct “raw” nuclear NF-κB fraction given in Eqs. (1), (2) and (3) for this spurious contribution following the generic formula:

$$ \mathrm{N}\mathrm{F}\hbox{-} {\upkappa \mathrm{B}}_{\mathrm{nuc}}/\mathrm{N}\mathrm{F}\hbox{-} {\upkappa \mathrm{B}}_{\mathrm{total}} = \frac{\mathrm{raw}\_\mathrm{N}\mathrm{F}\hbox{-} {\upkappa \mathrm{B}}_{\mathrm{nuc}}/\mathrm{N}\mathrm{F}\hbox{-} {\upkappa \mathrm{B}}_{\mathrm{total}} - \delta }{1 - \delta }. $$

The corrected values are used to produce figures as indicated in points (1), (2) and (3). The raw values are given in Additional file 4 and Additional file 5.

#### Quantification of frame-average nuclear and cytoplasmic abundancies of NF-κB and IκBα

The average nuclear fluorescence in NF-κB and IκBα channels, ***I***_n_^NFκB^ and ***I***_n_^IκBα^, are calculated by dividing the sum of nuclear fluorescence in respective channel by the number of cells in the analyzed frame. The average cell fluorescence in NF-κB and IκBα channels, ***I***_cell_^NFκB^ and ***I***_cell_^IκBα^, are calculated by dividing total frame fluorescence (with background correction) in respective channel by the number of cells. Finally, the average cytoplasmic fluorescence in NF-κB and IκBα channels, ***I***_c_^NFκB^ and ***I***_c_^IκBα^, is estimated as

***I***_c_^NFκB^ = ***I***_cell_^NFκB^ − ***I***_n_^NFκB^,  ***I***_c_^IκBα^ = ***I***_cell_^IκBα^ − ***I***_n_^IκBα^ .

Based on these frame-average values we generated the following figures:

- Figs. 2a and 4a, b, c and Additional file 3: Figure S7. Here ${\mathrm{I}\upkappa \mathrm{B}\upalpha}_{\mathrm{total}}/\mathrm{N}\mathrm{F}\hbox{-} {\upkappa \mathrm{B}}_{\mathrm{total}}=\kern0.5em \frac{{\boldsymbol{I}}_{\mathrm{cell}}^{\mathrm{I}\upkappa \mathrm{B}\upalpha}}{{\boldsymbol{I}}_{\mathrm{cell}}^{\mathrm{NF}\upkappa \mathrm{B}}}\ .$ Since the ratio of fluorescence in the NF-κB and IκBα channels depends on the laser intensities and specific fluorescence of antibodies, it cannot be determined up to an absolute value. Therefore, the values in these plots are normalized such that IκBα_total_/NF-κB_total_ averaged over all frames equals 1 for unstimulated cells.

- Fig. 2d: Nuclear NF-κB is ***I***_n_^NFκB^ averaged over analysed frames, normalized (like WB data) such that it assumes value of 1 in its maximum, i.e., for 15 min of TNFα stimulation. Cytoplasmic NF-κB is ***I***_c_^NFκB^ averaged over analysed frames, normalized (like WB data) such that it assumes value of 1 for unstimulated cells. Cytoplasmic IκBα is ***I***_c_^IκBα^ averaged over analysed frames, normalized such that it assumes value of 1 at 60 min after TNFα stimulation (i.e., in the maximum according to WB data). Let us notice that the normalization for LPS stimulation is applied jointly with that for TNFα stimulation.

### Western blotting

#### Cell-fractionation

Cells were seeded on a 100 mm tissue culture-treated dishes, at a density of 1,000,000/dish, and incubated overnight. After stimulation, cells were placed on ice, washed with ice-cold PBS, scraped from the dish in PBS and centrifuged (4 °C, 100 × g, 5 min). Cell pellet was then suspended in 1.5 ml of hypotonic cytoplasmic fraction buffer (20 mM HEPES pH 8.0, 0.2 % IGEPAL CA-630, 1 mM EDTA, 1 mM DTT, protease and phosphatase inhibitor cocktail, as above) and incubated on ice for 10 min with occasional shaking. After centrifugation (4 °C, 1700 × g, 5 min), supernatant was set aside and treated as the cytoplasmic fraction; pellet was washed in the same buffer and recentrifuged, and supernatant was discarded. Remaining pellet was suspended in 150 μl of nuclear fraction buffer (20 mM HEPES pH 8, 420 mM NaCl, 20 % glycerol, 1 mM EDTA, 1 mM DTT, protein and phosphatase inhibitors, as above), incubated on ice for 30 min with occasional mixing and then centrifuged at 4 °C, 10,000 × g, 10 min. Supernatant containing nuclear fraction was transferred to a fresh tube and left for further processing.

#### SDS-PAGE and Western blot

Cell lysate was used to determine protein concentration using Bradford method against BSA standard. Cell lysate was precipitated by adding trichloroacetic acid (TCA) to a final concentration of 10 % and keeping on ice for 30 min. After centrifugation at 4 °C, 12,000 g, 10 min. Protein pellet was washed by adding cold acetone, vortexing and re-centrifuging. Finally, proteins were resuspended in standard Laemmli sample buffer containing 10 mM DTT and boiled at 95 °C for 10 min. Equal amounts of each protein sample was loaded onto 10 % polyacrylamide gel and SDS-PAGE was performed with Mini-PROTEAN Tetra System (Bio-Rad). Proteins were transferred to nitrocellulose membrane using wet electrotransfer in the Mini-PROTEAN apparatus, according to the modified Towbin method (400 mA, 50 min). Membrane was rinsed with TBST (TBS buffer containing 0.1 % Tween-20) and blocked for 1 h with 5 % BSA/TBS or 5 % non-fat dry milk. Membranes were incubated at 4 °C overnight with one of the primary antibodies. Following antibodies were used: anti-p65 D14E12 (CST), anti-IκBα L35A5 (CST), anti-A20 D13H3 (CST), anti-GAPDH (EMD Millipore) and anti-HDAC-1 (Santa Cruz Biotechnology). After washing with TBST, membranes were incubated with secondary antibodies conjugated with horseradish peroxidase (Goat anti-rabbit and anti-mouse immunoglobulins/HRP, Dako) for 1 h, at room temperature. After washing, chemiluminescent reaction was developed with Clarity Western ECL system (Bio-Rad). Specific proteins were detected in the dark room on the medical X-ray film. After taking scans of western blots, densitometric quantification of protein bands was performed using ImageJ software using normalization against indicated reference proteins (GAPDH or HDAC-1).

### Gene expression analysis

#### RNA isolation and reverse transcription

Cells were seeded on 12-well plates at a density of 100,000 cells/well. Upon stimulation, cells were washed once with PBS and submitted to isolation of total RNA using PureLink RNA Mini Kit (ThermoFischer Scientific), following manufacturer’s instructions. Concentration and quality of isolated RNA was determined by measuring UV absorbance of diluted samples at 260 and 280 nm, using Multiskan GO Microplate Spectrophotometer (ThermoFischer Scientific). If not used immediately, RNA was stored for later use at –80 °C. Reverse transcription with random primers was performed from about 2 μg of template RNA using High Capacity cDNA Reverse Transcription Kit (ThermoFischer Scientific). Reaction was performed in Mastercycler Gradient thermal cycler (Eppendorf) under following conditions: 10 min 25 °C, 120 min 37 °C, and 5 min 85 °C.

#### Real-Time Polymerase Chain Reaction (RT-PCR)

RT-PCR was performed on a QuantStudio 12 K Flex Real-Time PCR system with Array Card block (ThermoFischer Scientific). Reverse transcribed cDNA (1000 ng) was mixed with reaction Master Mix and loaded onto TaqMan Array Card containing probes and primers including endogenous reference controls. Reaction was conducted using QuantStudio “Standard” protocol, with FAM/ROX chemistry. Upon completion, expression of target genes was analysed using comparative ΔCT method with QuantStudio 12 K Flex software, normalized against GAPDH gene expression. TaqMan assays Mm00477798_m1 and Mm00437121_m1 were used for analysing the expression of IκBα and A20 genes, respectively.

#### Digital PCR (dPCR)

Digital PCR measurements for IκBα and A20 genes were performed using QuantStudio 3D system (Life Technologies). Sample loaded onto QuantStudio 3D Digital PCR Chip was thermocycled using ProFlex PCR System (ThermoFischer Scientific) according to the manufacturer’s instruction. Chips were analysed using QuantStudio 3D Digital PCR Instrument and ANALYSIS SUITE cloud software. In the case of TNFα stimulation the dPCR, measurements were used to calculate absolute numbers of IκBα and A20 mRNA/cell. In the case of LPS stimulation the dPCR measurements were used to rescale RT-PCR data to absolute numbers of mRNA/cell.

## Reviewers’ comments

We thank the Reviewers for their valuable comments which helped us to improve the manuscript. These comments allowed us to view our study from a different angle, and for this reason we modified (and shortened) the title which now emphasizes the result of analysis of NF-κB information channel capacity shown in Fig. 5. Additionally, we reformulated the image analysis section in Methods hoping to improve its clarity. Below, we include our responses, which indicate also how the manuscript has been modified to address Reviewers’ concerns. We hope that the revised manuscript is now suitable for publication in Biology Direct.

### Reviewer’s report 1

Marek Kimmel, Rice University, Houston, TX, USA

**Reviewer’s summary:** *This is an interesting paper*, *contributing to the understanding of the mechanistic details of the active and passive transport between cytoplasm and nucleus using an important example of NF*-*kB. The message of the paper is very well documented*, *both experimentally and by simulations. I have three remarks or rather discussion item*, *which in my opinion are worthy of clarification*.

#### Reviewer’s recommendations to authors

1. *Naively*, *one obvious thing to do is to compare functioning of the system with importins and exportins to the system without these molecules. It seems easy computationally*, *but more difficult experimentally. It would be good to know the authors*’ *opinion on this and maybe some simulations carried out*.

**Authors’ response:** Knocking out or silencing importins presents an experimental difficulty, since it would disturb the functioning of an entire cell. However, in the case of NF-κB family interactions with importins are well established due to experiments in which NLS sequences in p65, and other NF-κB proteins were mutated, see [19, 20]. Wolynes group [73] and others [13, 14] have also shown that IκBα mask these NLS. Therefore the question we consider is not whether importins are indispensable for NF-κB regulation, but rather whether explicit modelling of importins–NF-κB interactions adds to the understanding of kinetics of the NF-κB pathway. We address this question in response to reviewer 2, by comparing models in which the importin binding step is modelled explicitly or lumped with NF-κB nuclear import step.

2. *In several places*, *the possibility of the cell optimizing this or that is mentioned. There are two caveats to such hypotheses. One is that most real*-*life systems* (*not only biological*) *are clearly suboptimal*, *but linger for long periods regardless* (*trilobites and human genome*, *being ad hoc examples*). *Why should cells be different*? *Second*, *optimization of two different processes may be contradictory* (*consider cancer cells dividing slower than normal cells*). *The NF*-*κB system is a multifunctional hub*, *so how to optimize such an object*? *Authors*’ *insights are welcome*.

**Authors’ response:** We agree that there are many systems that appear to be far from optimal. However, since NF-κB pathway has been evolutionarily conserved from *Drosophila* to mammals (Ghosh et al., 1998, Annu. Rev. Immunol. 16:225–260), and is on the first lines of defence against pathogens, we expect that it is optimized for fast responses. Additionally, since excessive inflammation can be harmful we hypothesize that it is also designed for fast shut-off.

3. *I like the* “*digitized*” *pulse*-*series experiment. However*, *what I would expect is that if a* “*1*” *is succeeded by a* “*0*”, *there should be some low*-*level transients observed*, *while there is a complete absence of signal. Might you explain how it is possible*? *Is this exactly what is also expected in an experiment* (*no such experiment has been performed*, *correct*?)?

**Authors’ response:** The model predicts that pulses lasting 15 min or less produce a single pulse of nuclear NF-κB with no tail (see Additional file 3: Figure S4) and this prediction is in agreement with experimental data at the population level [30, 64]. See also Additional file 3: Figure S5 and Ashall et al. [34] for single cell analysis of responses to repeated 5 min TNFα pulses, also showing no tail in NF-κB activity.

4. *DETAILS*: *Background*: *Please explain if exportins help export mRNA and proteins*, *while importins only help import proteins*, *or is the distinction more complex*.

**Authors’ response:** Importins and exportins, together termed karyopherins, are involved in the classical pathway of nuclear transport of proteins. On top of that, pre-microRNA and tRNA are also exported by members of the exportin family. In contrast, mRNA do not use karyopherins but instead are exported by a heterodimer of NXF1 and NXT1 proteins [4]. The distinctions amongst karyopherins, importins and exportins are now explained better in the Background section.

5. *Results*: “*NF*-*κB is transported back to the cytoplasm complexed with IκBα*, *which passes through nuclear pores after association with exportin 1*”. *Do you mean that exportin pulls IkBa*, *which in turn pulls NF*-*kB out of the nucleus*?

**Authors’ response:** IκBα binds NF-κB. After exportin 1 binds the NES of IκBα, it enables the free IκBα and IκBα:NF-κB complex to cross the nuclear pore. This is now clarified in the main text.

6. *Is dynamics of exporting of such large complex different from say*, *exporting of pure IkBa*?

**Authors’ response:** IκBα, having a mass of 32 kDa, is at least partially independent of importins and exportins, i.e. it can cross nuclear pores alone, see Fagerlund et al. [11]. Since IκBα is mostly cytoplasmic, we assume in the model that IκBα translocates to the nucleus independently of importins but uses exportins to translocate out of the nucleus.

7. “*For freely diffusing molecules the ratio of nuclear export to nuclear import* …” *Do you mean the ratio of rates*? *The reasoning outlined in this paragraph is pivotal for the paper*, *so it should be carefully phrased*.

**Authors’ response:** Yes, we mean the ratio of rates, we have now clarified it in revised text.

### Responses to Reviewer 2

**James Faeder**, University of Pittsburgh, Pittsburgh, PA, USA

**Reviewer’s summary:** *This paper presents an extended computational model of NF*-*κB signaling that specifically considers the interactions of NF*-*κB and IκBαlpha* (*IκBα*) *with the importin and exportin proteins that mediate nuclear import and export respectively. Experimental data from fibroblasts in the form of Western blots and fixed cell immunofluorescence staining is obtained that shows that at times between about 60 and 90 min the apparent concentration of IκBα in the cytoplasm exceeds that of NF*-*κB and yet NF*-*κB is still able to translocate in substantial amounts to the nucleus. It is claimed that this phenomenon can only be accurately described by the extended model that includes NF*-*κB interaction with importin. It is further argued that the ability of NF*-*κB to translocate to the nucleus even when the cytoplasmic concentration of IκBα exceeds that of NF*-*κB allows the system to reset more quickly following pulsatile stimulation than would otherwise be the case*, *which thus increases the maximum possible rate of information transmission*, *which is estimated to be 1 bit* (*based on the ability to detect only the presence or absence of TNF*) *times one over the reset time*, *which is estimated to be 60 min based on the simulations shown in* Fig. 5. *Overall*, *I see this work as a significant contribution to the ongoing effort to model and understand the mechanisms that influence NF*-*κB dynamics. I have some reservations about the claimed importance and novelty of the mechanisms being considered here*, *which I would like the authors to address prior to publication. In particular*, *the authors claim but do not demonstrate that the proposed model uniquely captures a key finding in their experimental data*, *which is that NF*-*κB translocation can continue even when the level of IκBα exceeds that of NF*-*κB. I would like to see a more conclusive demonstrate that the nuclear import mechanism the authors have explicitly added to their model is required to capture this effect*.

#### Reviewer’s recommendations to authors

1. *The main issue I have with this paper is that it is not clear from the presentation in the paper that the explicit consideration of karyopherin* (*importin*/*exportin*) *mediated transport actually results in a novel mechanism. It seems like any model that explicitly considers free cytoplasmic NF*-*κB will include a competition between binding to free IκBα and nuclear transport*, *although the parameters governing that competition could be different from those proposed in the current model. On p. 4 it is stated*, *however*, *that* “*although previous models assumed that free NF*-*κB may translocate to the nucleus*, *importin alpha binding precluding sequestration by IκBα was not explicitly considered. As a result*, *in these models efficient NF*-*κB translocation was possible only after the IκBα level dropped below that of NF*-*κB*.” *So*, *the authors are claiming the rate in these models was set so low that translocation could not compete with IκBα rebinding until IκBα levels fell below those of cytoplasmic NF*-*κB*, *but no references are given so it*’*s hard to tell which models the authors are referring to. I check one model that I*’*m familiar with*, *that of Lee* et al. (*2014*), *and this didn*’*t seem to be the case. Also*, *the model already has the competition mechanism built in*, *and it*’*s just a matter of varying the import rate relative to the binding rate to capture the mechanism that is described here*, *so although it*’*s useful to identify a mechanistic basis for this competition*, *it is not clear that the present model uniquely captures this mechanism. I think that to demonstrate that this competition parameter is indeed important for providing a correct description of the NF*-*κB*/*IκBα dynamics*, *they should show how varying the import rate affects the overall dynamics and also demonstrate how previous models do not capture this effect correctly*.

**Authors’ response:** The role of importins and exportins in NF-κB regulation is well documented. The questions is whether explicit implementation of the NF-κB–importin binding step is important in modelling. We think it is, and it can be explained as follows. In the model without importins, in the presence of free IκBα, released NF-κB may translocate to the nucleus if the expected entry time is shorter (or at least comparable) with expected binding time with IκBα. The Reviewer is right that this can be assured in the model by assuming sufficiently fast nuclear translocation of NF-κB. However, in the reality the NF-κB translocation time is controlled by the size of the cell and its nucleus, diffusion coefficient, binding with importins, and the translocation through nuclear pores. It is therefore possible that imposing constrains on NF-κB translocation time (in order to make it shorter than NF-κB–IκBα binding time) we obtain the wrong picture of spatial regulation of the system. This has important implications for the more detailed reaction-diffusion models that are emerging in recent years for NF-κB and other regulatory systems (see Terry & Chaplain [42] and Sturrock et al. [41]).

In the model with importins it is NF-κB may enter the nucleus despite elevated levels of IκBα provided that expected NF-κB–importin binding time is shorter than the expected binding time with IκBα. We expect that this condition holds until concentration of free cytoplasmic IκBα is smaller than that of importins.

Therefore, we think that explicitly accounting for NF-κB–importin interactions is important in order to bring modelling to a more precise mechanistic description. This does not mean that the models that lumped together various reactions may not serve as an reasonable description. We now formulate presentation of our results in a more modest way.

We also supplement the Results section with a new figure (Fig. 6), in which we compare our model with its variant that lumps together the processes of NF-κB–importin binding and NF-κB nuclear translocation. The analysis is performed in correspondence to pulsed stimulation considered in Fig. 5 (in response to a suggestion by Reviewer 3, we introduce this figure in the Results section). Fig. 5 shows that the system can transmit information about NF-κB pulses as long as their frequency is not larger than 1/h. From the stochastic time profiles shown in Fig. 5 one can see that the response to second pulse TNFα is critical, i.e. the amplitude of the response to the second pulse is the lowest. Therefore in Fig. 6 we compare two model variants analysing the ratio of the second to the first peak amplitude and the difference between levels of IκBα in its second minimum and at *t* = 0. The comparison is done as a function of IκBα–NF-κB binding rate and NF-κB nuclear import coefficient. Generally in the novel model, the NF-κB translocation at the second peak is higher and is accompanied by a smaller decrease in the level of IκBα. The difference between two models is pronounced for small NF-κB import coefficient and for high IκBα–NF-κB binding rate, and as expected by the Reviewer, it vanishes when NF-κB import coefficient is large.

Additionally, we consider a more detailed model in which the three processes of NF-κB binding, complex translocation to the nucleus and importin dissociation are considered separately. As a reminder, in the original model we lumped processes of NF-κB nuclear translocation and importins dissociation in the nucleus. We demonstrate that this more detailed description enhances the effect of importins. Nonetheless, one should keep in mind that this is also a simplified picture, as in reality NF-κB is first bound by importin-α3 or α4, which are in turn bound by importin-β, the ternary complex diffuses into the vicinity of the nucleus and passes through nuclear pores. Next, in the nucleus importin β dissociates in response to RanGTP binding, and only then importin-α may dissociate from NF-κB. Since the affinity between importin-α and NLS is high (typically 10 nM) this process must be also somehow induced [4].

Regarding the values that the coefficients mentioned above take in existing NF-κB models, in Lee et al. [38] the authors assume IκBα–NF-κB binding rate equal 0.5 (μM s)^-1^ and NF-κB nuclear import rate equal 0.0026 s^-1^. Assuming NF-κB concentration equal 0.1 μM (Lee et al. scan the range 0.04–0.4 μM; the value 0.1 μM corresponding to roughly 10^5^ was estimated by Carlotti et al. [53, 54], and assuming that IκBα concentration exceeds that of NF-κB by 50 % (i.e., assuming that there is 0.05 μM of free IκBα) we obtain the pseudo-first order NF-κB binding rate equal 0.025 s^-1^ (versus NF-κB nuclear import rate 0.0026 s^-1^). This implies that a released NF-κB molecule has about a 10 times higher chance of binding a free IκBα molecule than of translocating to the nucleus. In our earlier model [33] the NF-κB nuclear import rate was equal 0.01 s^-1^, while IκBα–NF-κB binding rate was equal 5 × 10^-7^ s^-1^. These values mean that NF-κB nuclear translocation is 2.5 times less probable than IκBα binding. The substantially different import coefficient is assumed/fitted in the models developed by Levchenko and Hoffmann (see Werner et al. [64] and Werner et al., 2005, Science 309:1857–61). In these models NF-κB import rate is 0.09 s^-1^, while the IκBα–NF-κB binding rate is also equal 0.5 (μM s)^-1^. Therefore in these models the NF-κB translocation outcompetes IκBα binding. We expect however, that NF-κB import rate is rather of order of 0.01 s^-1^ (or smaller) than of order of 0.1 s^-1^ (which would imply average translocation time of 10 s). To our knowledge, the NF-κB import rate was never measured directly.

2. *Another possible problem with the modeling and inference here is the discrepancy between model and experiment that is displayed in* Fig. 3c, *about which I could not find any comment in the manuscript. The issue is this*: *in the experiment the IκBα level continues to rise between 60 and 90 min while at the same time the amount of nuclear NF*-*κB rises and both IκBα and NF*-*κB remain elevated at 180 min. The model*, *on the other hand*, *exhibits a decrease in the IkB level on the same time interval. This result begs the question how in the experiment the NF*-*κB level can rise as the IkB level also rises*, *but the model doesn*’*t display this behavior and hence can*’*t provide an explanation. The model clearly shows that IκBα and NF*-*κB oscillate out of phase*, *whereas the measured levels do not. It seems likely that some other mechanism is at play here*, *which is not being captured by the model. Another issue that concerns the modeling and also the interpretation of the experimental data is the basal level of NF*-*κB in the nucleus. On p. 6 it is stated that* “*WB analysis indicate*[*s*] *that in unstimulated cells the nuclear NF*-*κB level is very low*.” *That is indeed what is shown in* Fig. 2d, *but it is contradicted by the fixed cell imaging data shown in* Fig. 2a *and in* Fig. 3a, c, *which indicate than an average of about 20* % *of NF*-*κB is in the nucleus prior to stimulation. The model does not capture this effect*, *which calls into question whether is it also missing some key aspects of the IkB*/*NF*-*κB interaction dynamics. Something curious about the initial conditions of the model is also revealed by looking at the black points in* Fig. 4a and b *showing the initial conditions in individual cells for the experiments and model respectively. Whereas the experiments exhibit considerable variability in the fractional amount of nuclear NF*-*κB and relatively little variation in the ratio of IkB*/*NF*-*κB*, *the model shows little nuclear NF*-*κB but considerable variability of in the relative amount of IkB. How might this discrepancy affect the observed results*?

**Authors’ response:** Since there is no Fig. 3c, we think that the Reviewer means Fig. 4c. Indeed, there is a discrepancy between the model and single cell data, as observed by the Reviewer. The model was fitted to the population data obtained in the form of Western blots (see Fig. 2d). The immunofluorescence single cell data were provided to show that also at single cell level NF-κB translocation is possible even when IκBα exceeds initial levels. By analysing single cell images we rule out the possibility that IκBα level is very high only in the fraction of cells that do not exhibit second NF-κB translocation.

In fact this effect is more pronounced when analysed at the level of immunostaining images. As shown in Figs. 2d and 4c the average IκBα level (between 100 and 180 min) calculated based on immunofluorescence images is higher than obtained in Wester blots, and surprisingly it increases between 60 and 100 min apparently in phase with nuclear NF-κB.

The discrepancy between the fraction of nuclear NF-κB in unstimulated cell obtained by quantified Western blots and immunofluorescence images follows possibly from overshadowing of nuclei by cytoplasm, which is hard to avoid even when using confocal microscopy. This overshadowing depends on cell morphology and we failed to fully correct it by our quantification method (see Methods).

Considering the above, we think that population data better represent the average NF-κB and IκBα levels, while image-based single-cell quantification can give some insight into heterogeneity of the response. In the revised manuscript we mention and briefly discuss these discrepancies.

### Reviewer’s Report 3

**William Hlavacek**, CNLS, Los Alamos, NM, USA

**Reviewer’s summary:** *Korwek* et al. *report results from a study that involved both experimentation and modeling. The study was focused on understanding oscillations in nuclear localization of the transcription factor NF*-*kappaB in response to stimulation by an endotoxin* (*lipopolysaccharide*, *LPS*) *or a cytokine* (*TNFalpha*). *These signals induce the degradation of IkappaB*, *which is responsible for sequestering NF*-*kappaB in the cytosol. Degradation of IkappaB allows NF*-*kappaB to concentrate in the nucleus*, *which leads to new synthesis of IkappaB. The authors explain how*, *after an initial pulse of nuclear localization*, *NF*-*kappaB is able to concentrate in the nucleus a second time even though the overall abundance of its inhibitor IkappaB rises above its baseline abundance before the second pulse of nuclear localization. The explanation is that IkappaB must compete for binding to NF*-*kappaB with importin alpha proteins*, *which are karyopherins that mediate transport of NF*-*kappaB into the nucleus. It seems that this report offers an answer to a puzzling question about the dynamics of NF*-*kappaB nuclear localization. I think this report would be rather interesting to other researchers working on regulation of NF*-*kappaB. I suppose the major weakness of this report would be that the conclusions of the authors about the influence of karyopherins on NF*-*kappaB dynamics have not been directly tested*, *for example*, *by modulation of the strength of interaction between RELA and KPNA2*.

#### Reviewer’s recommendations to authors

1. *There are some points in the report of Korwek* et al. *that could be clarified. I wonder if the authors could present more illustrative simulations or introduce a simplified model to more clearly explain how the competition between IkappaB and importin alpha gives rise to the faster*-*than*-*expected oscillations in NF*-*kappaB nuclear localization. I*’*m not confident that I was able to fully appreciate the authors*’ *insights*.

**Authors’ response:** In the revised manuscript we provide a comparison between the model with and without explicitly accounting for importins (see Fig. 6 and the response to Reviewer 2).

2. *I think that competition alone is not the only deciding factor but rather it is the competition in combination with the fact that there are two different compartments where NF*-*kappaB can be found* (*cytosol and nucleus*). *In any case*, *I would appreciate a clearer explanation of the role of karyopherins in NF*-*kappaB nuclear localization dynamics*.

**Authors’ response:** Yes, the Reviewer is indeed right that the discussed effect is the competition of IκBα and importin-α in combination with the fact that there are two different cellular compartments where NF-κB can be found. In the revised manuscript we clarified role of *karyopherins in NF*-*kappaB nuclear localization dynamics*.

3. *It is not entirely clear from the manuscript as written if the above*-*baseline level of IkappaB during the second pulse of NF*-*kappaB nuclear localization is a novel observation of the authors being reported for the first time here*, *or rather a previously observed phenomenon*.

**Authors’ response:** To our knowledge, we are the first to show that in single cells nuclear NF-κB translocation coincides with above-baseline levels of IκBα. Although in a report by Fagerlund et al. (2015) [11] NF-κB translocation and elevated IκBα are also shown by Western blotting at 90-120 min after stimulation, only our immunofluorescence single-cell data demonstrate that this effect cannot be explained by high accumulation of IκBα in some cells and nuclear translocation in others.

4. *The authors make several assumptions about protein copy numbers. I think these assumptions could be bolstered by referring to protein copy numbers reported by the Mann group for various mammalian cell lines*, *such as the report by Geiger T* et al. (*2012*) [*Mol Cell Proteomics DOI* 10.1074/mcp.M111.014050].

**Authors’ response:** The use of data detailing the exact protein copy number is indeed very compelling. Concerning the work of Geiger et al. (2012), however, we found the values included in the paper unsuitable for our model. First of all, all eleven cell lines screened for proteins by Geiger et al. (2012) were of human origin and mostly of epithelial phenotype (we are aware that it does not necessarily preclude this data from use in modelling of MEFs). Secondly, in most of these cell lines copy numbers for all of the pertinent proteins like RelA, IκBα and A20 were not quantified simultaneously. Only three lines (GAMG, Jurkat and LnCap) had iBAQ values quantified for all three of these proteins and some of these values come from only single replicate or exhibit quite significant intra-replicate variance. Furthermore, these data suggest that copy number of RelA exceeds that of IκBα by one order of magnitude, or in some cases even two (e.g. log-transformed IBAQ values for RelA and IκBα in the GAMG cell line are around 7 and 5.2, respectively). Although we admire the scope of the cited paper, we find these values hard to reconcile with our current understanding of the role of IκBα as the main RelA inhibitor.

We have decided to use the estimations of NF-κB copy number included in the works of Carlotti et al. [53, 54], as stated in the manuscript, while the values for other proteins were mostly predicted by the model.

5. *It would be appreciated if the authors provided the HGNC names for proteins* (e.g., *NF*-*KBIA for IkappaB*).

**Authors’ response:** We now provide HGNC names for genes in revised manuscript.

6. *The description of nuclear trafficking is incomplete. For example*, *RAN is never mentioned. A more complete description of nuclear trafficking would be helpful*.

**Authors’ response:** We include more detailed description of nuclear trafficking and discuss the role of RAN.

7. *Furthermore*, *the authors may wish to acknowledge that modeling of nuclear trafficking has been considered in the past*, *as in the work of Zilman A*., *Effects of multiple occupancy and interparticle on selective transport through narrow channels*: *theory* versus *experiment. Biophysical Journal Volume 96 February 2009 1235*–*1248*

**Authors’ response:** We refer to the work of Zilman et al. [28] and the recent work of Lolodi et al. [29] in the Background section.

8. *In the abstract*, *the authors assume that readers will know that importins and exportins are karyopherins. The word* “*karyopherin*” *should probably be defined upon first use*.

**Authors’ response:** We define the term karyopherins in the revised manuscript.

9. *The authors state that BioNetGen implements the Gillespie algorithm. It would be more precise to state that BioNetGen implements an efficient variation of Gillespie*’*s direct method*.

**Authors’ response:** This is now corrected it in the revised manuscript.

10. *It is said that TNFalpha is unstable* in vivo *but stable* “*under experimental conditions*.” *Could the authors say more about how conditions affect TNFalpha stability*? *Does* “*resynthesized*” *mean* “*newly synthesized*?”

**Authors’ response:** We discussed TNFα stability in vivo and in vitro studies in revised manuscript in the section “Formulation of the computational model”.

11. *Formulation of the computational model. The abbreviation* “*WB*” *should be defined upon first use*.

**Authors’ response:** This is now corrected it in the revised manuscript.

12. *In the abstract*, *the authors make a point about information transmission rate*, *but this issue is next considered only in the Discussion section. It*’*s odd that* Fig. 5 *is not cited in the Results section. The authors claim that information channel capacity depends on n and tau without explaining or citing a source. It would be helpful if the authors could say more about this point and cite appropriate supporting references*.

**Authors’ response:** We now include Fig. 5 in the Results section and clarify what we mean by information channel capacity and how it is estimated.

13. *The authors claim that the rates of transcription and translation for IkappaB and A20 are near their maximum values. How are the maximum values estimated*? *Could the authors cite appropriate supporting references for the estimates of the maximum rates*?

**Authors’ response:** We discussed how these rates are estimated and included appropriate references in the section “Formulation of the computational model” in revised manuscript.

## Additional files

Additional file 1: Computational model description. (PDF 349 kb)
Additional file 2: Computational model in BNGL. (BNGL 14 kb)
Additional file 3:
**Figure S1.** Analysis of functional TNFα degradation in experimental conditions. (a) Immunostaining confocal images of unstimulated cells and cells stimulated for 15 and 30 min with fresh TNFα at 10 ng/ml concentration. (b) Immunostaining images of cells stimulated for 15 and 30 min with the media harvested from above cells stimulated for 6 h with TNFα, at the initial concentration of 10 ng/ml. (c,d) Simulated single cell (thin lines) and population average (bold lines) trajectories in responses to TNFα stimulation with concentration *D*_1_ = 10 ng/ml (orange lines) and *D*_2_ = 1.15 ng/ml (blue lines). At the assumed TNFα degradation coefficient c_deg_ = 10^-4^/s the initial TNFα concentration *D*_1_ is reduced to *D*_2_ after 6 h. **Figure S2.** Model simulation trajectories showing unstimulated, equilibrated cells. **Figure S3.** Model simulation trajectories for A20-deficient cells in response to 10 ng/ml TNFα stimulation, studied experimentally by Lee et al. [12]. As in experiment, A20-deficient cells respond by a stable NF-κB translocation. **Figure S4.** Model simulated responses to single 10 ng/ml TNFα pulses of various durations. Simulations correspond to experimental data [30, 64] showing single NF-κB pulses of amplitude almost independent of pulse duration. **Figure S5.** Model simulated responses to the series of three 5 min, 10 ng/ml TNFα pulses, with pulse repeat of 60 min, 100 min, 200 min; corresponding to the experiment by Ashall et al. [34], who observed that almost all cells respond to first pulse, while about 30 % fraction of cells respond to the second and third pulse for 60 min, 100 min repeats. For 200 min repeats almost all cells respond to three TNFα pulses. **Figure S6.** Model simulated responses to repeated 10 ng/ml TNFα pulses corresponding to the experiment by Zambrano et al. [40], who observed NF-κB oscillations in response to pulses repeated every 45 min. **Figure S7.** Scatter plots showing evolution of the total IκBα/total NF-κB ratio and nuclear NF-κB/total NF-κB ratio in response to 1 μg/ml LPS. The scatter plot is based on quantified confocal images shown in Additional file 5. (PDF 3475 kb)
Additional file 4: Full confocal images with marked cells used for fluorescence quantification after 10 ng/ml TNFα stimulation for 0, 15, 30, 60, 90 and 180 min. Data table show raw nuclear to total NF-κB ratio and normalized total IκBα to total NF-κB ratio for each analysed cell (for quantification details see Methods). (PDF 1380 kb)
Additional file 5: Full confocal images with marked cells used for fluorescence quantification after 1 μg/ml LPS stimulation. Data table show raw nuclear to total NF-κB ratio and normalized total IκBα to total NF-κB ratio for each analysed cell (for quantification details see Methods). (PDF 1238 kb)
Additional file 6: Full confocal images with marked nuclei used for NF-κB fluorescence quantification after 5 μg/ml CHX + 1 μg/ml LPS stimulation. CHX stimulation starts 60 min before LPS. (PDF 1087 kb)
Additional file 7: Full confocal images of cells stimulated with 10 ng/ml TNFα for 0, 5, 10, 15, 20, and 30 min. (PDF 733 kb)

## Abbreviations

CHX: Cycloheximide
IKK: IκB kinase
IRF3: Interferon regulatory factor 3
IκBα: Nuclear factor of kappa light polypeptide gene enhancer in B-cells inhibitor α
LPS: Lipopolysaccharide
NES: Nuclear export sequence
NF-κB: Nuclear factor kappa-light-chain-enhancer of activated B cells
NLS: Nuclear localization sequence
STAT1: Signal transducer and activator of transcription 1
TF: Transcription factor
TNFR: Tumor necrosis factor α receptor
TNFα: Tumor necrosis factor α
WB: Western blot
