# Supplementary material for: Importins promote high-frequency NF-κB oscillations increasing information channel capacity
Source: Biol Direct. 2016 Nov 11;11:61. doi: 10.1186/s13062-016-0164-z (PMC5106790; doi:10.1186/s13062-016-0164-z)
Supplement: Additional file 1: — Computational model description. (PDF 349 kb) [file 13062_2016_164_MOESM1_ESM.pdf]

Importins promote high-frequency NF-κB oscillations increasing information channel capacity

Zbigniew Korwek[=], Karolina Tudelska[=], Paweł Nałęcz-Jawecki[=], Maciej Czerkies[=], Wiktor Prus, Joanna Markiewicz, Marek Kochańczyk, and Tomasz Lipniacki

([=] — equal contribution)

Computational model description

Supplementary information: Additional file 1

1 Parameters and reactions

Table A. Notation guide

Symbol	*Description*
TNF	TNFα trimers
$TNFR_i$	inactive TNFR receptors
$TNFR_a$	active TNFR receptors
$IKKK_n$	neutral form of IKKK
$IKKK_a$	active form of IKKK
IKK_i	inactive form of IKK
IKK_{ii}	inactive intermediate form of IKK
IKK_n	neutral form of IKK kinase
IKK_a	active form of IKK
$A20_{mRNA}$	A20 transcript
A20	A20 protein
$I\kappa B\alpha_{mRNA}$	IκBα transcript
$I\kappa B\alpha_c$	cytoplasmic IκBα
$I\kappa B\alpha_{c,p}$	phosphorylated cytoplasmic IκBα
$I\kappa B\alpha_n$	nuclear IκBα
$I\kappa B\alpha_{n,\mathbf{e}}$	nuclear IκBα bound to an exportin
$NF\kappa B_c$	cytoplasmic NF-κB
$NF\kappa B_{c,\mathbf{i}}$	cytoplasmic NF-κB bound to an importin
$NF\kappa B_n$	nuclear NF-κB
$NF\kappa B_c \cdot I\kappa B\alpha_c$	cytoplasmic NF-κB·IκBα complexes
$NF\kappa B_c \cdot I\kappa B\alpha_{c,p}$	phosphorylated IκBα complexed with NF-κB in the cytoplasm
$NF\kappa B_n \cdot I\kappa B\alpha_n$	nuclear NF-κB·IκBα complexes
$NF\kappa B_n \cdot I\kappa B\alpha_{n,\mathbf{e}}$	nuclear complex of NF-κB and IκBα bound to an exportin
$g^i_{I\kappa B\alpha}$	state of the ith IκBα gene copy, discrete random variable: $g^i_{I\kappa B\alpha} \in \{0, 1\}$
g^i_{A20}	state of the ith A20 gene copy, discrete random variable: $g^i_{A20} \in \{0, 1\}$

Table B. Cell parameters

Parameter	*Symbol*	*Value*	*Remarks*	*Refs*
C:N ratio = $\frac{\text{Volume of cytoplasm}}{\text{Volume of nucleus}}$	k_{v}	5	—	[L07]
Number of TNFRs	R	735	The median number of receptors is assumed equal 735, and this value was used in deterministic simulations. In stochastic simulations, the number of TNFRs is drawn from log-normal distribution with mean $= 2\times10^3$ and $\sigma = \sqrt{2}$, which gives median = mean$\times\exp(-1) \approx 7\times10^2$. $R = \mathit{TNFR}_{\mathrm{a}}(t)+\mathit{TNFR}_{\mathrm{i}}(t)$	[T10]
Number of IKKK molecules	K_{N}	10^5	$K_{\mathrm{N}} = \mathit{IKKK}_{\mathrm{n}}(t) + \mathit{IKKK}_{\mathrm{a}}(t)$	[T10]
Number of IKK molecules	K_{NN}	2×10^5	$K_{\mathrm{NN}} = \mathit{IKK}_{\mathrm{n}}(t) + \mathit{IKK}_{\mathrm{a}}(t) + \mathit{IKK}_{\mathrm{i}}(t) + \mathit{IKK}_{\mathrm{ii}}(t)$	[L07]
Number of NF-κB molecules	$\mathit{NF\kappa B}_{\mathrm{tot}}$	10^5	The median number of NF-κB molecules that can be imported into the nucleus is assumed equal 70 000, whereas the median number of inert (non-translocatable) NF-κB molecules (that are bound to inhibitors other than IκBα and in response to TNF are very slowly degraded or not degraded) is assumed 30 000. These numbers are used for deterministic simulations. In stochastic simulations, both molecule numbers are drawn independently from an experimental distributions of NF-κB levels (shown in Fig. 2b in the main text) which has been rescaled so that the median number of translocable NF-κB molecules is 70 000 and median of non-translocatable NF-κB molecules is 30 000.	[this study]
Number of IκBα gene copies	N_{I}	2	—	[T10]
Number of A20 gene copies	N_{A}	2	—	[T10]

(For references, see page 4.)

Table C. List of reactions

Reaction	*Rate*	*Coefficients*	*Value*	*References*
TNFR activation and signal transduction cascade				
TNF→ ∅	c_{deg}	c_{deg}	10^{-4} s^{-1}	[this study]
$\mathrm{TNFR_i} \rightarrow \mathrm{TNFR_a}$	$\dfrac{k_{\mathrm{b}} \cdot TNF}{TNF_{\mathrm{cell}}}$	k_{b}<br>TNF_{cell}	1.2×10^{-5} s^{-1}<br>6×10^{4} $^{(\star)}$	[T10]<br>(see footnote)
$\mathrm{TNFR_i} \leftarrow \mathrm{TNFR_a}$	k_{f}	k_{f}	1.2×10^{-3} s^{-1}	[T10]
$\mathrm{IKKK_n} \rightarrow \mathrm{IKKK_a}$	$\dfrac{k_{\mathrm{a}} \cdot k_{\mathrm{A20}}}{k_{\mathrm{A20}} + A20} \cdot TNFR_{\mathrm{a}}$	k_{a}<br>k_{A20}	2×10^{-5} s^{-1}<br>10^{5}	[this study]<br>[T10]
$\mathrm{IKKK_n} \leftarrow \mathrm{IKKK_a}$	k_{i}	k_{i}	10^{-2} s^{-1}	[T10]
$\mathrm{IKK_n} \rightarrow \mathrm{IKK_a}$	$k_1 \cdot IKKK_{\mathrm{a}}^2$	k_1	6×10^{-10} s^{-1}	[T10]
$\mathrm{IKK_a} \rightarrow \mathrm{IKK_i}$	$\dfrac{k_3}{k_2} \cdot (k_2 + A20)$	k_2<br>k_3	5×10^{3}<br>2×10^{-3} s^{-1}	[this study]<br>[T10]
$\mathrm{IKK_i} \rightarrow \mathrm{IKK_{ii}}$, $\mathrm{IKK_{ii}} \rightarrow \mathrm{IKK_n}$	k_4	k_4	2×10^{-3} s^{-1}	[this study]
IκBα and A20 gene expression				
$(g^i_{\mathrm{A20}} = 0) \rightarrow (g^i_{\mathrm{A20}} = 1)$<br>$(g^i_{\mathrm{I\kappa B\alpha}} = 0) \rightarrow (g^i_{\mathrm{I\kappa B\alpha}} = 1)$	$q_1 \cdot NF\kappa B_{\mathrm{n}}$	q_1	10^{-7} s^{-1}	[this study]
$(g^i_{\mathrm{A20}} = 0) \leftarrow (g^i_{\mathrm{A20}} = 1)$<br>$(g^i_{\mathrm{I\kappa B\alpha}} = 0) \leftarrow (g^i_{\mathrm{I\kappa B\alpha}} = 1)$	$q_2 \cdot I\kappa B\alpha_{\mathrm{n}}$	q_2	5×10^{-7} s^{-1}	[this study]
$\varnothing \rightarrow \mathrm{A20_{mRNA}}$	$c_1 \cdot g^i_{\mathrm{A20}}$	c_1	0.2 s^{-1}	[this study]
$\varnothing \rightarrow \mathrm{I\kappa B\alpha_{mRNA}}$	$c_1 \cdot g^i_{\mathrm{I\kappa B\alpha}}$			
$\varnothing \leftarrow \mathrm{A20_{mRNA}}$	$c_{3\mathrm{a}}$	$c_{3\mathrm{a}}$	1.5×10^{-3} s^{-1}	[this study]
$\varnothing \leftarrow \mathrm{I\kappa B\alpha_{mRNA}}$	$c_{3\mathrm{i}}$	$c_{3\mathrm{i}}$	7.5×10^{-4} s^{-1}	[L07]
$\varnothing \rightarrow \mathrm{A20}$	$c_4 \cdot A20_{\mathrm{mRNA}}$	c_4	0.5 s^{-1}	[L07]
$\varnothing \rightarrow \mathrm{I\kappa B\alpha}$	$c_4 \cdot I\kappa B\alpha_{\mathrm{mRNA}}$			
Protein interactions and lifetime				
$\mathrm{NF\kappa B_c} + \mathrm{I\kappa B\alpha_c} \rightarrow \mathrm{NF\kappa B_c{\cdot}I\kappa B\alpha_c}$	a_1	a_1	10^{-7} mlcl^{-1} s^{-1}	[this study]
$\mathrm{NF\kappa B_n} + \mathrm{I\kappa B\alpha_n} \rightarrow \mathrm{NF\kappa B_n{\cdot}I\kappa B\alpha_n}$	$a_1 \cdot k_{\mathrm{v}}$	k_{v}	5	[L07]
$\mathrm{I\kappa B\alpha_c} \rightarrow \mathrm{I\kappa B\alpha_{c,p}}$	$a_2 \cdot IKK_{\mathrm{a}}$	a_2	10^{-7} s^{-1}	[L07]
$\mathrm{NF\kappa B_c{\cdot}I\kappa B\alpha_c} \rightarrow \mathrm{NF\kappa B_c{\cdot}I\kappa B\alpha_{c,p}}$	$a_3 \cdot IKK_{\mathrm{a}}$	a_3	5×10^{-7} s^{-1}	[L07]
$\mathrm{A20} \rightarrow \varnothing$	c_5	c_5	5×10^{-4} s^{-1}	[L07]
$\mathrm{I\kappa B\alpha_{c,p}} \rightarrow \varnothing$<br>$\mathrm{NF\kappa B_c{\cdot}I\kappa B\alpha_{c,p}} \rightarrow \mathrm{NF\kappa B_c}$	t_{p}	t_{p}	10^{-2} s^{-1}	[L07]
$\mathrm{I\kappa B\alpha_c} \rightarrow \varnothing$	$c_{5\mathrm{a}}$	$c_{5\mathrm{a}}$	3×10^{-4} s^{-1}	[this study]
$\mathrm{NF\kappa B_c{\cdot}I\kappa B\alpha_c} \rightarrow \mathrm{NF\kappa B_c}$	$c_{6\mathrm{a}}$	$c_{6\mathrm{a}}$	2×10^{-5} s^{-1}	[L07]
Karyopherins and transport				
$\mathrm{I\kappa B\alpha_c} \rightarrow \mathrm{I\kappa B\alpha_n}$	$i_{1\mathrm{a}}$	$i_{1\mathrm{a}}$	7×10^{-4} s^{-1}	[this study]
$\mathrm{NF\kappa B_c} \rightarrow \mathrm{NF\kappa B_{c,\mathbf{i}}}$	$b_{\mathbf{i}_{\mathrm{N}}}$	$b_{\mathbf{i}_{\mathrm{N}}}$	0.1 s^{-1}	[this study]
$\mathrm{NF\kappa B_n{\cdot}I\kappa B\alpha_n} \rightarrow \mathrm{NF\kappa B_n{\cdot}I\kappa B\alpha_{n,\mathbf{e}}}$	$b_{\mathbf{e}_{\mathrm{I}}}$	$b_{\mathbf{e}_{\mathrm{I}}}$	5×10^{-3} s^{-1}	[this study]
$\mathrm{I\kappa B\alpha_n} \rightarrow \mathrm{I\kappa B\alpha_{n,\mathbf{e}}}$				
$\mathrm{NF\kappa B_{c,\mathbf{i}}} \rightarrow \mathrm{NF\kappa B_n}$	$i_{\mathbf{i}_{\mathrm{N}}}$	$i_{\mathbf{i}_{\mathrm{N}}}$	10^{-2} s^{-1}	[this study]
$\mathrm{NF\kappa B_n{\cdot}I\kappa B\alpha_{n,\mathbf{e}}} \rightarrow \mathrm{NF\kappa B_c{\cdot}I\kappa B\alpha_c}$	$e_{\mathbf{e}_{\mathrm{I}}} \cdot k_{\mathrm{v}}$	$e_{\mathbf{e}_{\mathrm{I}}}$	10^{-2} s^{-1}	[this study]
$\mathrm{I\kappa B\alpha_{n,\mathbf{e}}} \rightarrow \mathrm{I\kappa B\alpha_c}$		k_{v}	5	[L07]

$^{(\star)}$ TNF_{cell} – number of TNF trimers/cell at 1 ng/ml TNF stimulation at the cell density of 2×10^{5}/ml.

2 Methods and protocols of numerical simulations

Deterministic model

The model, provided in Additional file 2, is specified in BioNetGen language, which is intended for defining regulatory networks of high combinatorial complexity [F09]. BioNetGen software is capable of performing both deterministic and stochastic simulations of model dynamics. BioNetGen allows for rule-based specification of the model; the rules are used to build a system of ODEs which are solved by an integrated CVODE solver.

Stochastic simulations

The stochastic simulations were performed using the Gillespie direct Stochastic Simulation Algorithm [G77] as implemented in BioNetGen. The simulator allows for expressing reaction rates through functions of the system state, i.e., current number of molecules of given species. This allows for defining propensities not necessarily following the mass action kinetics. At every time step between consecutive reaction events, reaction propensities calculated using such functions remain constant.

To ensure random initial conditions at time $t = 0$, each stochastic simulation was started at time t drawn at random uniformly from the interval [−110 hr,−100 hr]. The population average was obtained from 300 simulations.

Supplementary references

[T10] Tay S, Hughey J, Lee T, Lipniacki T, Covert M, Quake M (2010) Single-cell NF-κB dynamics reveal digital activation and analogue information processing. *Nature* **466**:267–271.

[L07] Lipniacki T, Puszynski T, Paszek P, Brasier AR, Kimmel M (2007) Single TNFα trimers mediating NF-κB activation: Stochastic robustness of NF-κB signaling. *BMC Bioinformatics* **8**:376.

[F03] Faeder JR, Blinov ML, Hlavacek WS (2009) Rule-based modeling of biochemical systems with BioNetGen. *Methods Mol. Biol.* **500**:113–167.

[G77] Gillespie DT (1977) Exact stochastic simulations of coupled chemical reactions. *J. Phys. Chem.* **81**:2340–2361.
