# Supplementary material for: Importins promote high-frequency NF-κB oscillations increasing information channel capacity
Source: Biol Direct. 2016 Nov 11;11:61. doi: 10.1186/s13062-016-0164-z (PMC5106790; doi:10.1186/s13062-016-0164-z)
Supplement: Additional file 3: — Figure S1. Analysis of functional TNFα degradation in experimental conditions. (a) Immunostaining confocal images of unstimulated cells and cells stimulated for 15 and 30 min with fresh TNFα at 10 ng/ml concentration. (b) Immunostaining images of cells stimulated for 15 and 30 min with the media harvested from above cells stimulated for 6 h with TNFα, at the initial concentration of 10 ng/ml. (c,d) Simulated single cell (thin lines) and population average (bold lines) trajectories in responses to TNFα stimulation with concentration D 1 = 10 ng/ml (orange lines) and D 2 = 1.15 ng/ml (blue lines). At the assumed TNFα degradation coefficient cdeg = 10-4/s the initial TNFα concentration D 1 is reduced to D 2 after 6 h. Figure S2. Model simulation trajectories showing unstimulated, equilibrated cells. Figure S3. Model simulation trajectories for A20-deficient cells in response to 10 ng/ml TNFα stimulation, studied experimentally by Lee et al. [12]. As in experiment, A20-deficient cells respond by a stable NF-κB translocation. Figure S4. Model simulated responses to single 10 ng/ml TNFα pulses of various durations. Simulations correspond to experimental data [30, 64] showing single NF-κB pulses of amplitude almost independent of pulse duration. Figure S5. Model simulated responses to the series of three 5 min, 10 ng/ml TNFα pulses, with pulse repeat of 60 min, 100 min, 200 min; corresponding to the experiment by Ashall et al. [34], who observed that almost all cells respond to first pulse, while about 30 % fraction of cells respond to the second and third pulse for 60 min, 100 min repeats. For 200 min repeats almost all cells respond to three TNFα pulses. Figure S6. Model simulated responses to repeated 10 ng/ml TNFα pulses corresponding to the experiment by Zambrano et al. [40], who observed NF-κB oscillations in response to pulses repeated every 45 min. Figure S7. Scatter plots showing evolution of the total IκBα/total NF-κB ratio and nuclear NF-κB/total NF-κB ratio in [file 13062_2016_164_MOESM3_ESM.pdf]

Importins promote high-frequency NF-κB oscillations increasing information channel capacity

Zbigniew Korwek[=], Karolina Tudelska[=], Paweł Nałęcz-Jawecki[=], Maciej Czerkies[=], Wiktor Prus, Joanna Markiewicz, Marek Kochańczyk, and Tomasz Lipniacki

([=] equal contribution)

Supplementary Figures S1–S7

Supplementary information: Additional File 1

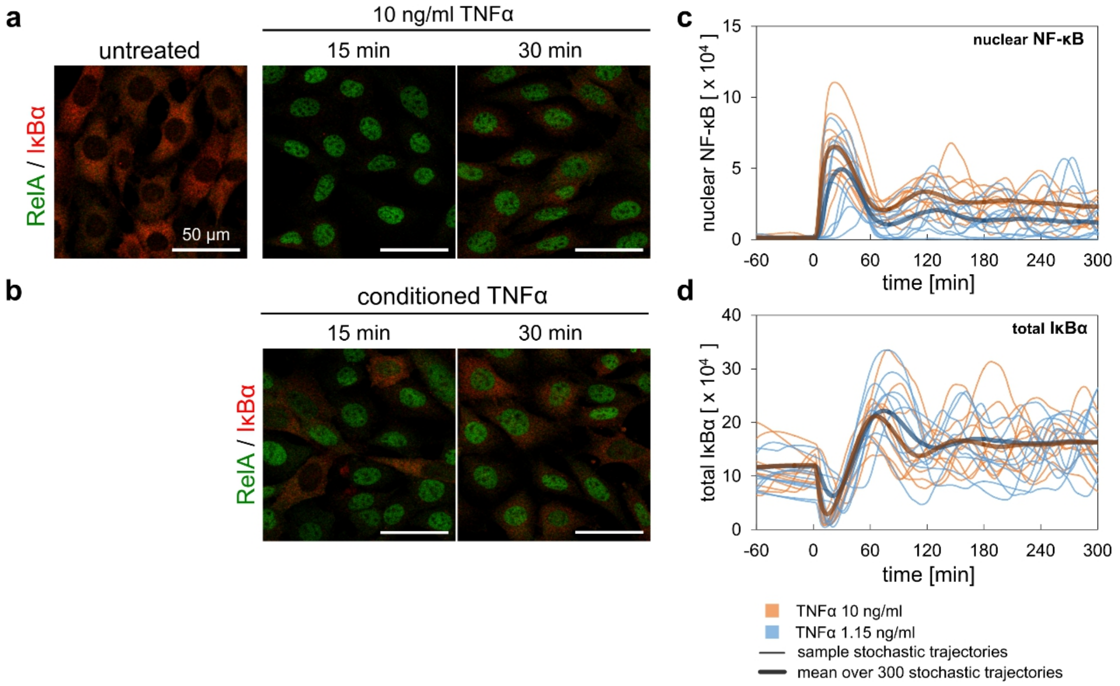

Fig. S1. Analysis of functional TNFα degradation in experimental conditions. (**a**) Immunostaining confocal images of unstimulated cells and cells stimulated for 15 and 30 min with fresh TNFα at 10 ng/ml concentration. (**b**) Immunostaining images of cells stimulated for 15 and 30 min with the media harvested from above cells stimulated for 6 h with TNFα, at the initial concentration of 10 ng/ml. (**c**,**d**) Simulated single cell (thin lines) and population average (bold lines) trajectories in responses to TNFα stimulation with concentration D_1 = 10 ng/ml (orange lines) and D_2 = 1.15 ng/ml (blue lines). At the assumed TNFα degradation coefficient $c_{deg} = 10^{-4}$/s the initial TNFα concentration D_1 is reduced to D_2 after 6 hr.

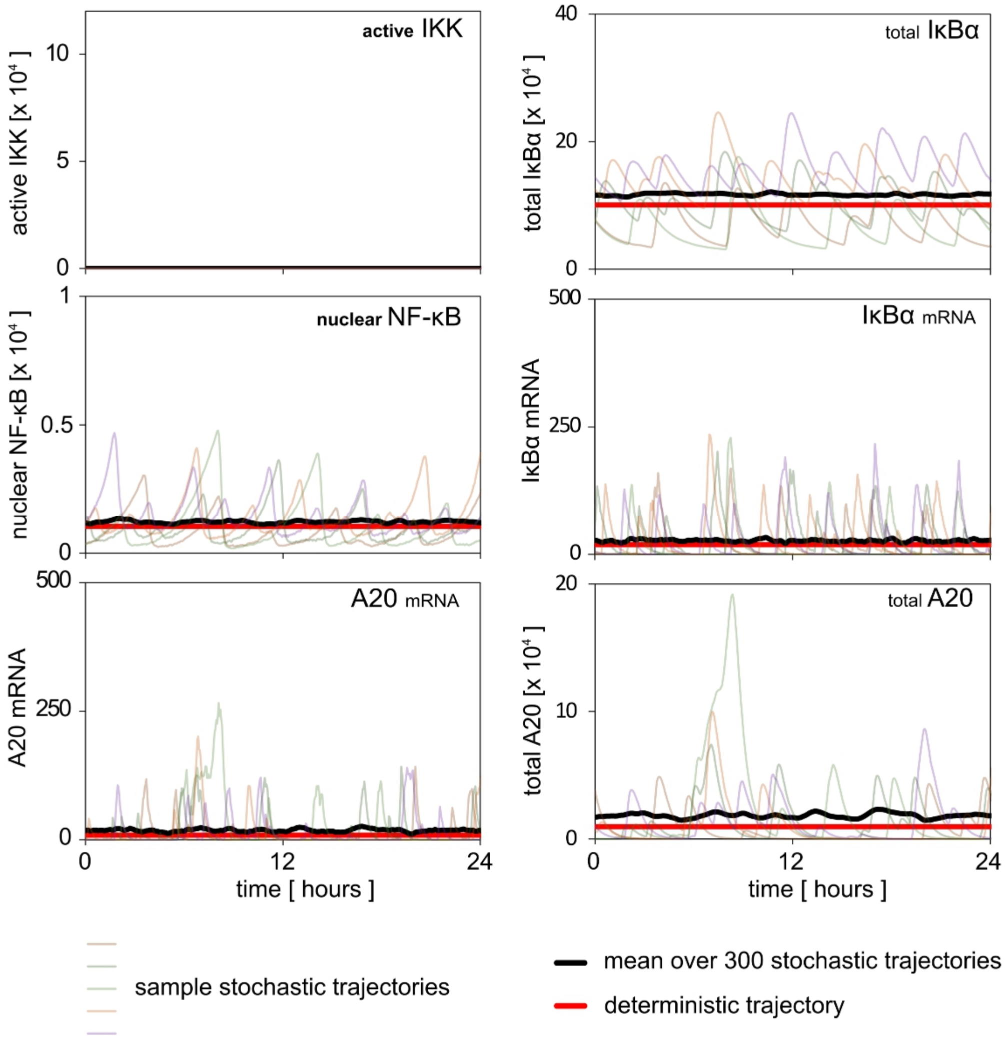

Fig. S2. Model simulation trajectories showing unstimulated, equilibrated cells.

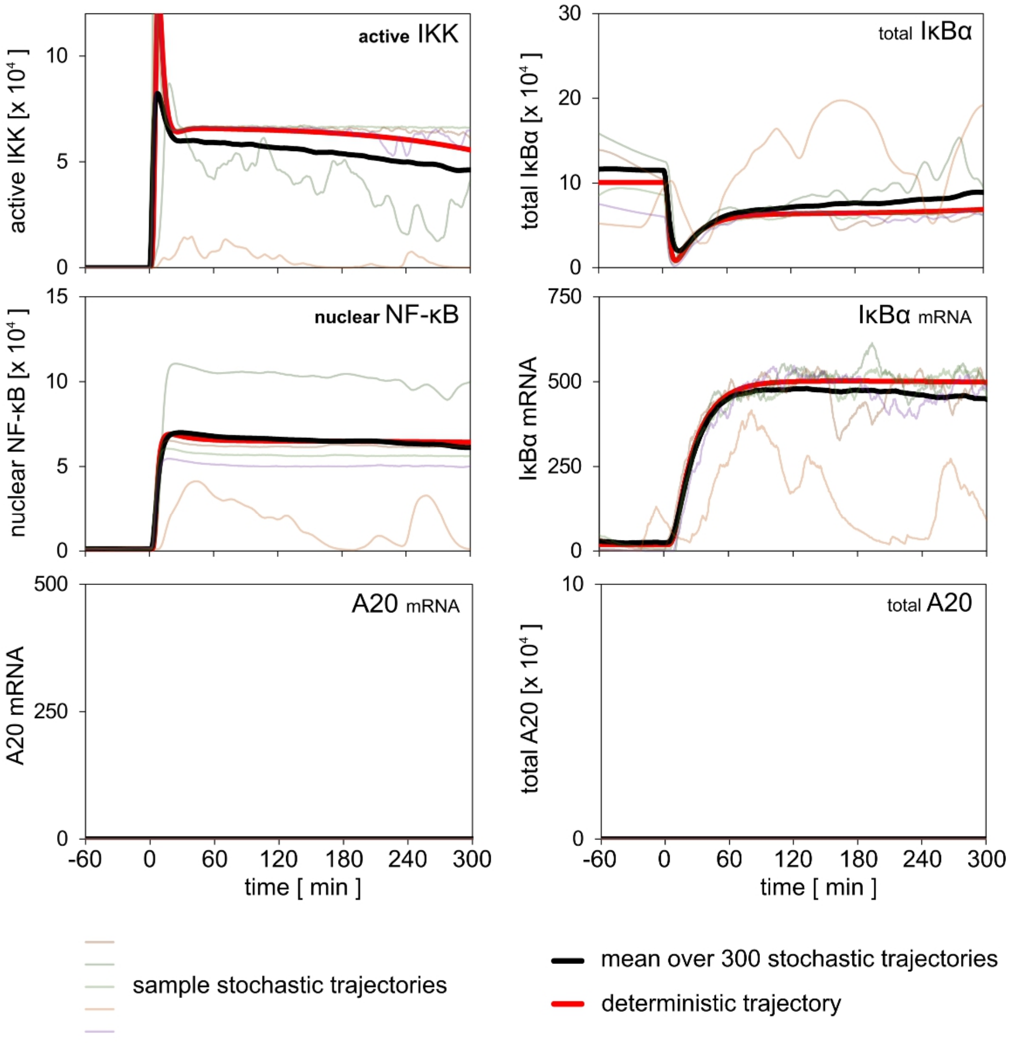

Fig. S3. Model simulation trajectories for A20-deficient cells in response to 10 ng/ml TNFα stimulation, studied experimentally by Lee et al. [12]. As in the experiment, A20-deficient cells respond by a stable NF-κB translocation.

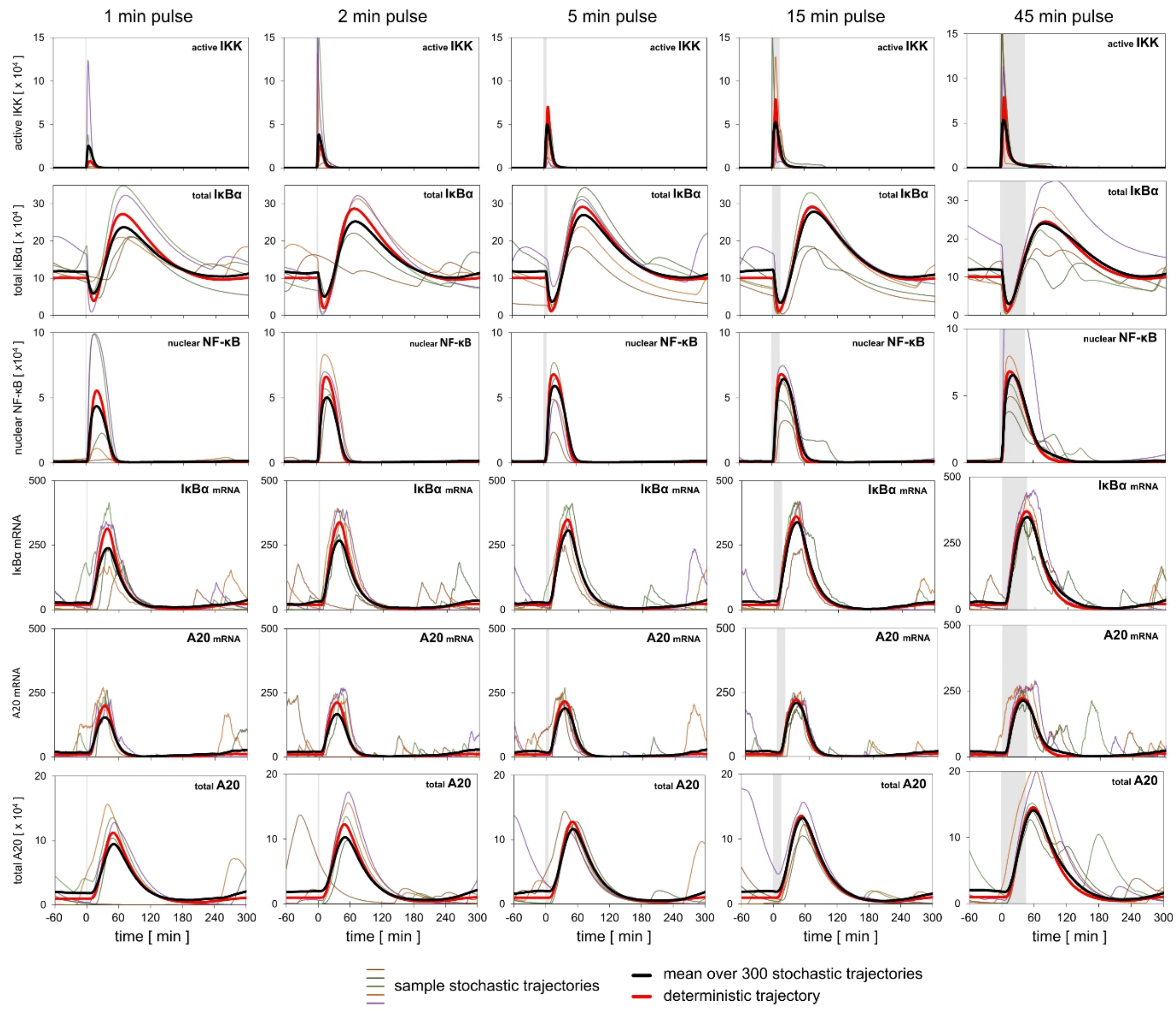

Fig. S4. Model simulated responses to single 10 ng/ml TNFα pulses of various durations. Simulations correspond to experimental data [30,64], showing single NF-κB pulses of amplitude almost independent of pulse duration.

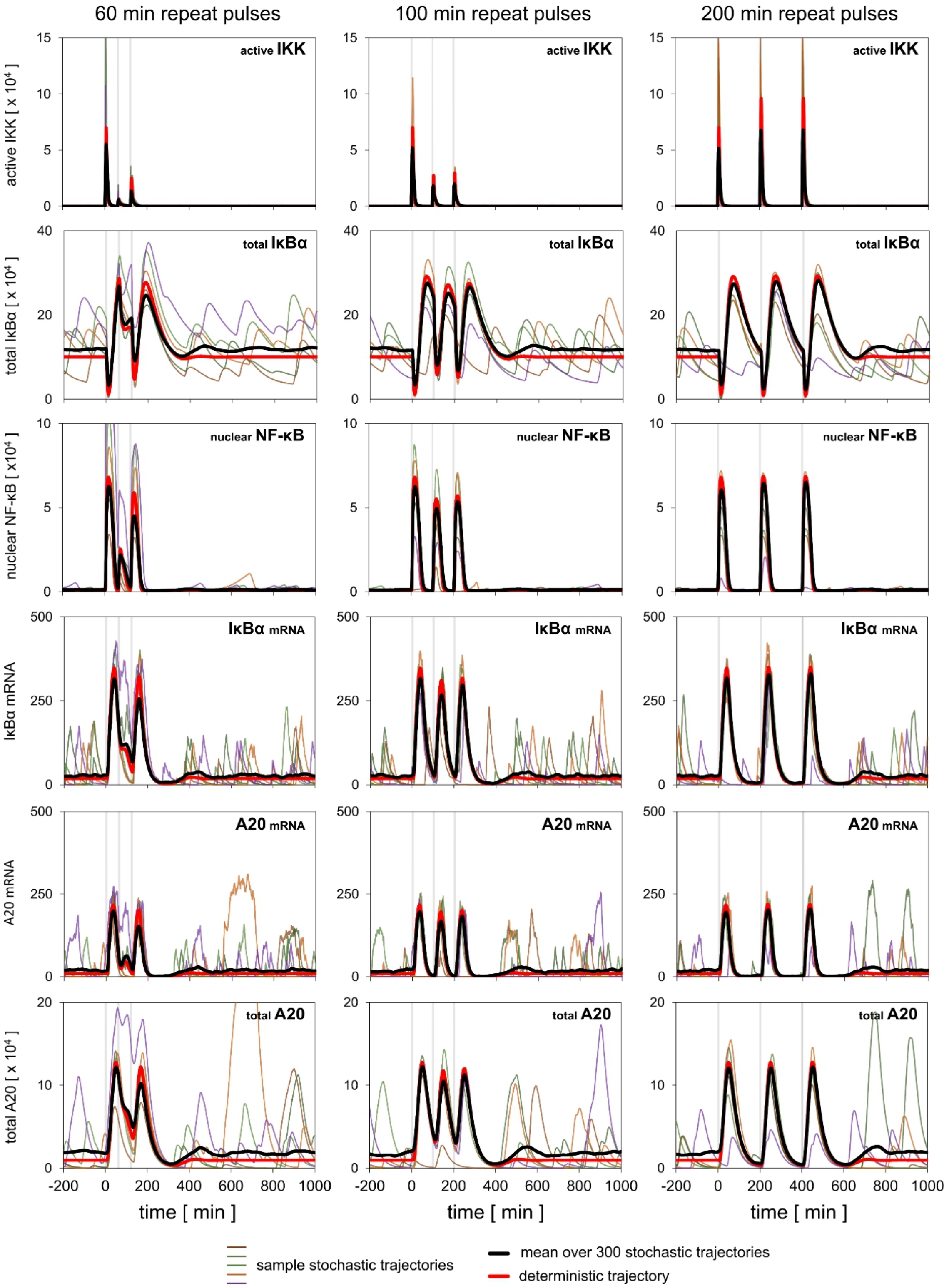

Fig. S5. Model simulated responses to the series of three 5 min, 10 ng/ml TNFα pulses, with pulse repeat of 60 min, 100 min, 200 min; corresponding to the experiment by Ashall et al. [34], who observed that almost all cells respond to first pulse, while about 30% fraction of cells respond to the second and third pulse for 60 min, 100 min repeats. For 200 min repeats almost all cells respond to three TNFα pulses.

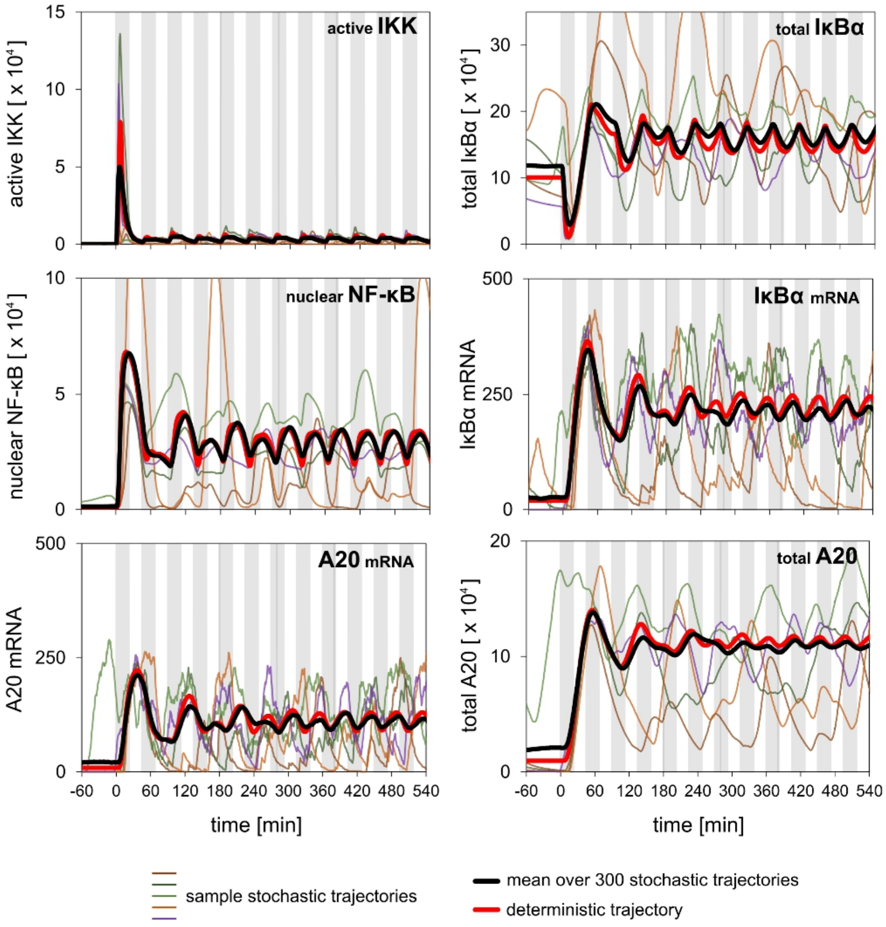

Fig. S6. Model simulated responses to repeated 10 ng/ml TNFα pulses corresponding to the experiment by Zambrano et al. [40], who observed NF-κB oscillations in response to pulses repeated every 45 min.

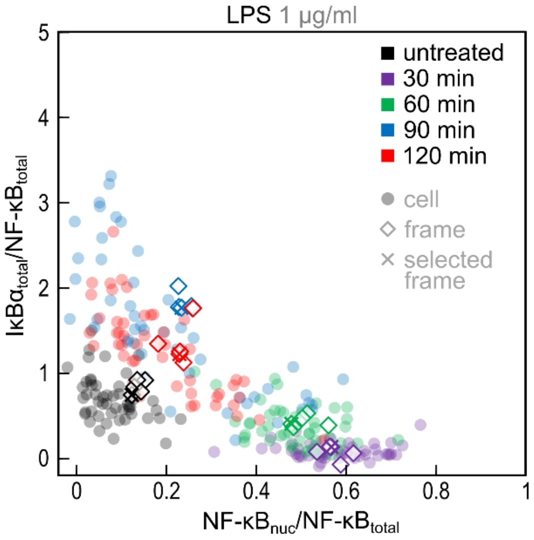

Fig. S7. Scatter plots showing evolution of the total IκBα/total RelA ratio and nuclear NF-κB/total NF-κB ratio in response to 1 µg/ml LPS. The scatter plot is based on quantified confocal images shown in Additional file 5.
