# Supplementary material for: Importins promote high-frequency NF-κB oscillations increasing information channel capacity
Source: Biol Direct. 2016 Nov 11;11:61. doi: 10.1186/s13062-016-0164-z (PMC5106790; doi:10.1186/s13062-016-0164-z)
Supplement: Additional file 4: — Full confocal images with marked cells used for fluorescence quantification after 10 ng/ml TNFα stimulation for 0, 15, 30, 60, 90 and 180 min. Data table show raw nuclear to total NF-κB ratio and normalized total IκBα to total NF-κB ratio for each analysed cell (for quantification details see Methods). (PDF 1380 kb) [file 13062_2016_164_MOESM4_ESM.pdf]

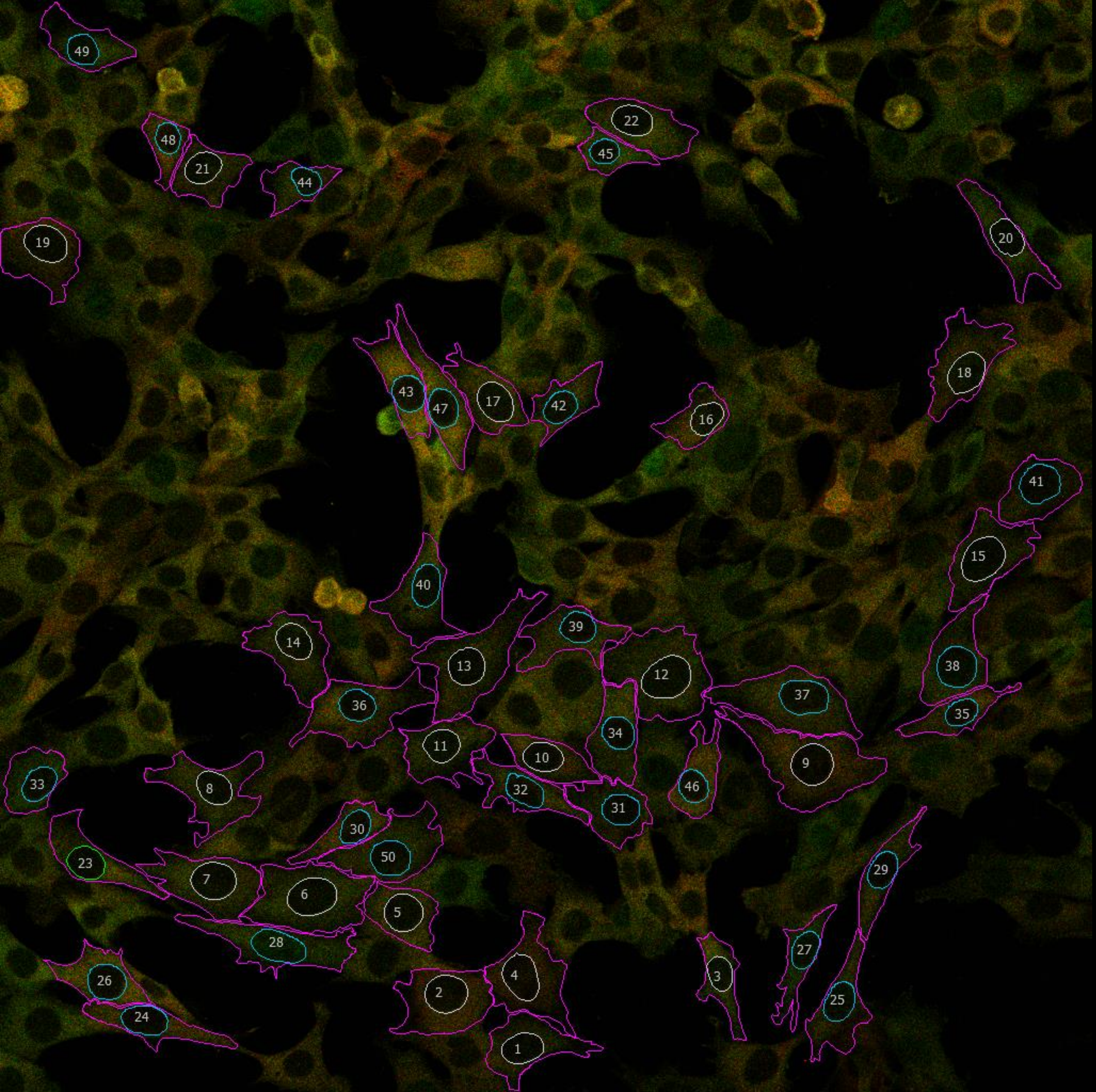

0 min - untreated

#	NFkB (nuc/total)	IkBa(total)/NFkB(total)
1	0.172	1.034
2	0.170	1.397
3	0.223	0.894
4	0.205	1.448
5	0.202	1.016
6	0.137	0.880
7	0.166	0.920
8	0.139	0.946
9	0.104	1.269
10	0.208	1.102
11	0.183	0.884
12	0.158	0.895
13	0.107	0.973
14	0.198	0.915
15	0.198	0.940
16	0.260	1.088
17	0.224	1.050
18	0.232	1.106
19	0.189	1.210
20	0.141	0.783
21	0.211	1.005
22	0.125	0.909
23	0.137	0.951
24	0.154	1.203
25	0.209	1.048
26	0.149	1.089
27	0.270	0.634
28	0.295	0.639
29	0.119	0.960
30	0.186	1.161
31	0.157	0.958
32	0.203	1.002
33	0.185	0.690
34	0.101	0.920
35	0.154	1.196
36	0.097	1.149
37	0.167	1.010
38	0.199	0.877
39	0.107	1.019
40	0.172	0.907
41	0.235	1.085
42	0.239	1.035
43	0.148	1.160
44	0.242	0.872
45	0.157	0.775
46	0.162	1.174
47	0.143	1.083
48	0.241	1.274
49	0.181	0.749
50	0.134	0.931

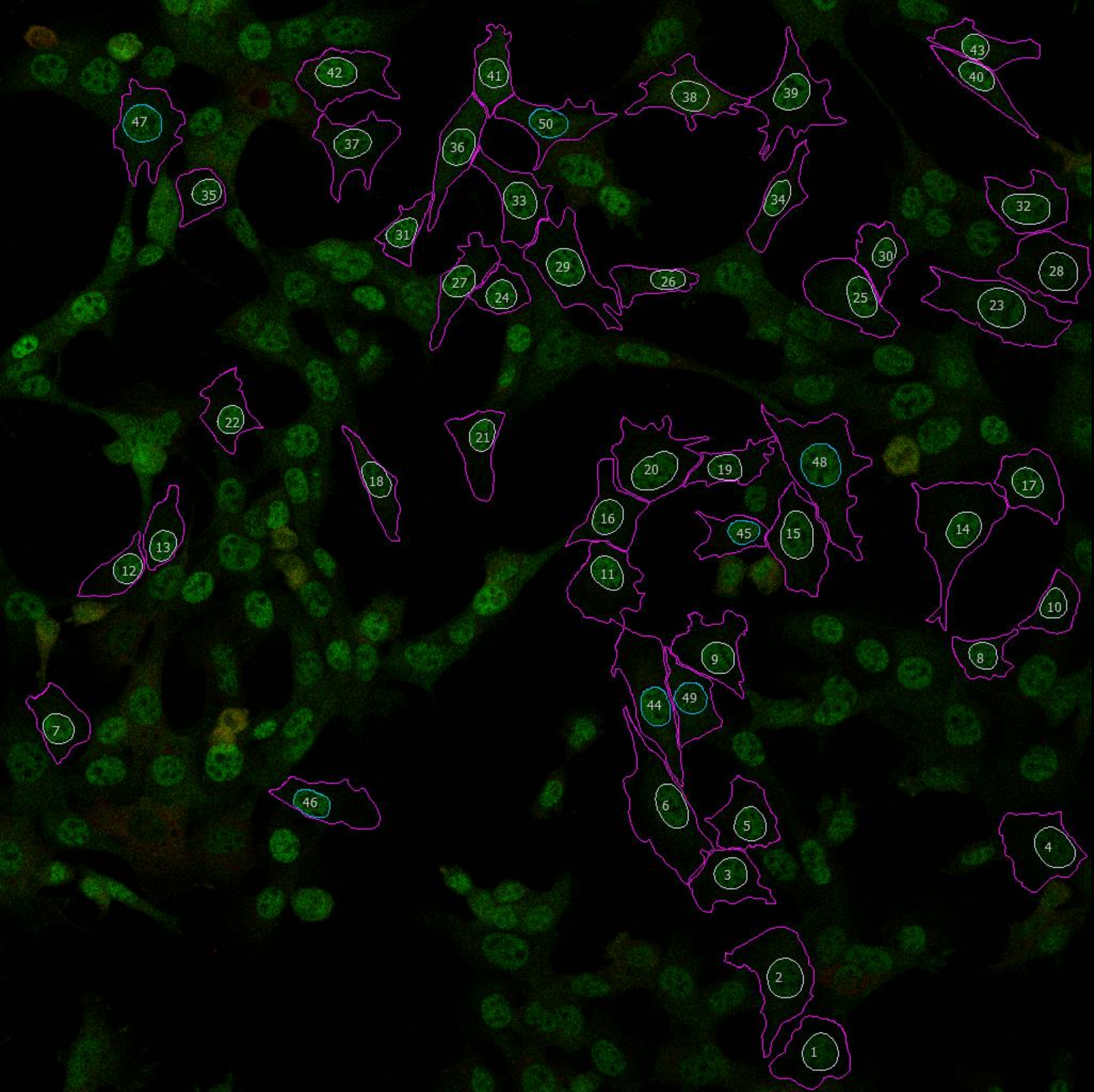

15 min TNFα 10 ng/ml

#	NFkB (nuc/total)	IkBa(total)/NFkB(total)
1	0.807	0.135
2	0.518	0.217
3	0.777	0.136
4	0.689	0.125
5	0.755	0.151
6	0.551	0.170
7	0.685	0.133
8	0.734	0.109
9	0.688	0.128
10	0.680	0.131
11	0.702	0.176
12	0.776	0.157
13	0.699	0.122
14	0.638	0.144
15	0.731	0.165
16	0.657	0.160
17	0.663	0.139
18	0.631	0.213
19	0.728	0.140
20	0.762	0.156
21	0.753	0.190
22	0.754	0.142
23	0.571	0.176
24	0.832	0.113
25	0.543	0.144
26	0.665	0.218
27	0.702	0.123
28	0.619	0.291
29	0.716	0.111
30	0.571	0.134
31	0.795	0.097
32	0.683	0.135
33	0.629	0.149
34	0.693	0.192
35	0.765	0.131
36	0.511	0.132
37	0.640	0.154
38	0.442	0.325
39	0.745	0.138
40	0.684	0.137
41	0.453	0.160
42	0.672	0.209
43	0.610	0.174
44	0.542	0.234
45	0.779	0.172
46	0.712	0.107
47	0.558	0.120
48	0.628	0.233
49	0.490	0.143
50	0.535	0.223

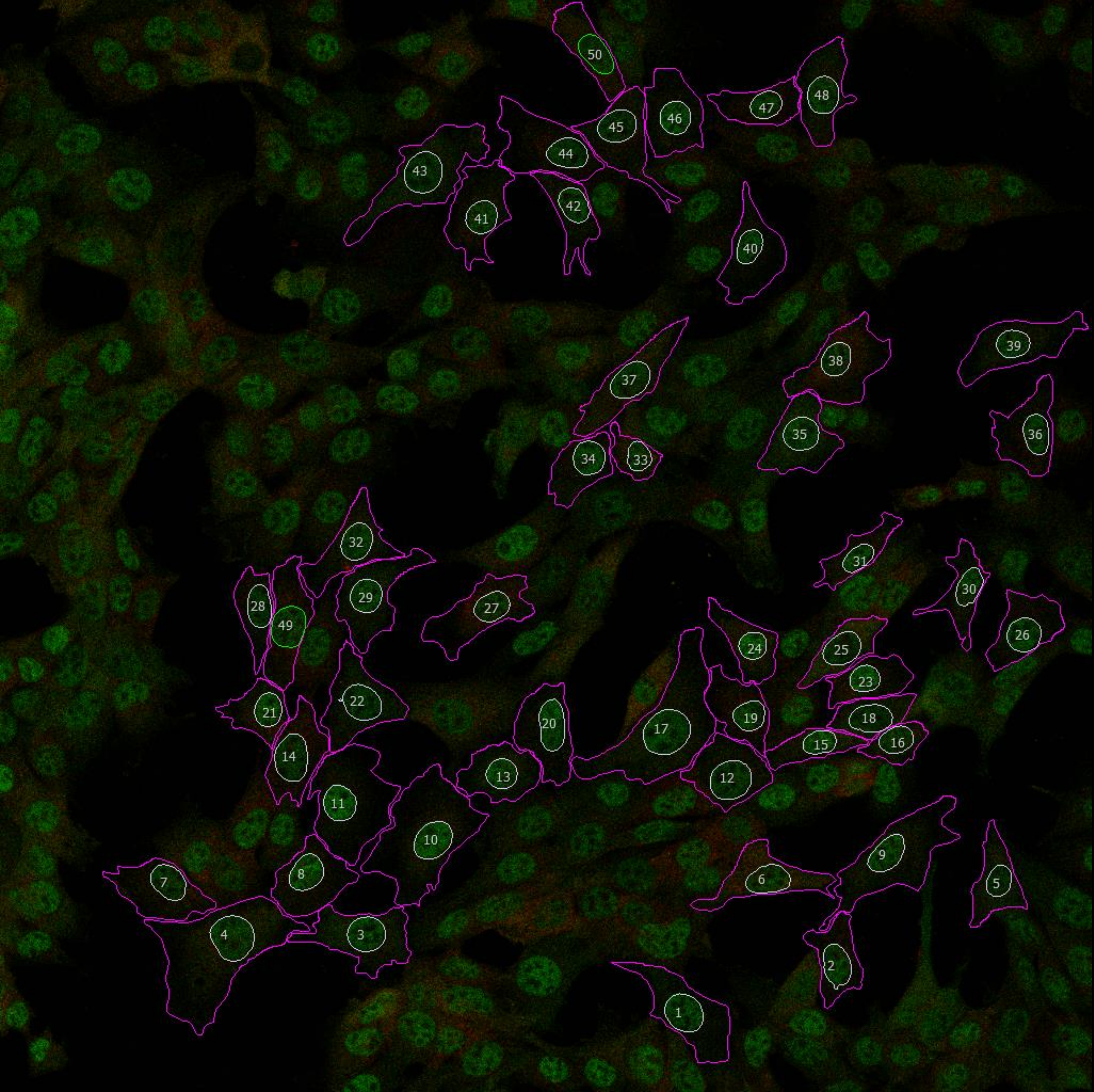

30 min TNFα 10 ng/ml

#	NFkB (nuc/total)	IkBa(total)/NFkB(total)
1	0.585	0.293
2	0.708	0.543
3	0.475	0.408
4	0.408	0.605
5	0.344	0.535
6	0.414	0.923
7	0.532	0.683
8	0.640	0.497
9	0.463	0.476
10	0.531	0.466
11	0.607	0.353
12	0.584	0.521
13	0.510	0.369
14	0.639	0.800
15	0.565	0.528
16	0.632	0.544
17	0.451	0.461
18	0.528	0.484
19	0.638	0.712
20	0.679	0.299
21	0.629	0.567
22	0.466	0.376
23	0.628	0.549
24	0.584	0.594
25	0.399	0.650
26	0.653	0.404
27	0.526	1.015
28	0.668	0.484
29	0.477	0.380
30	0.635	0.448
31	0.595	0.583
32	0.537	0.537
33	0.585	0.724
34	0.619	0.466
35	0.413	0.367
36	0.605	0.612
37	0.439	0.696
38	0.358	1.018
39	0.466	0.487
40	0.572	0.381
41	0.546	0.578
42	0.624	0.465
43	0.383	0.396
44	0.587	0.659
45	0.463	0.642
46	0.592	0.370
47	0.687	0.770
48	0.522	0.477
49	0.637	0.703
50	0.519	0.829

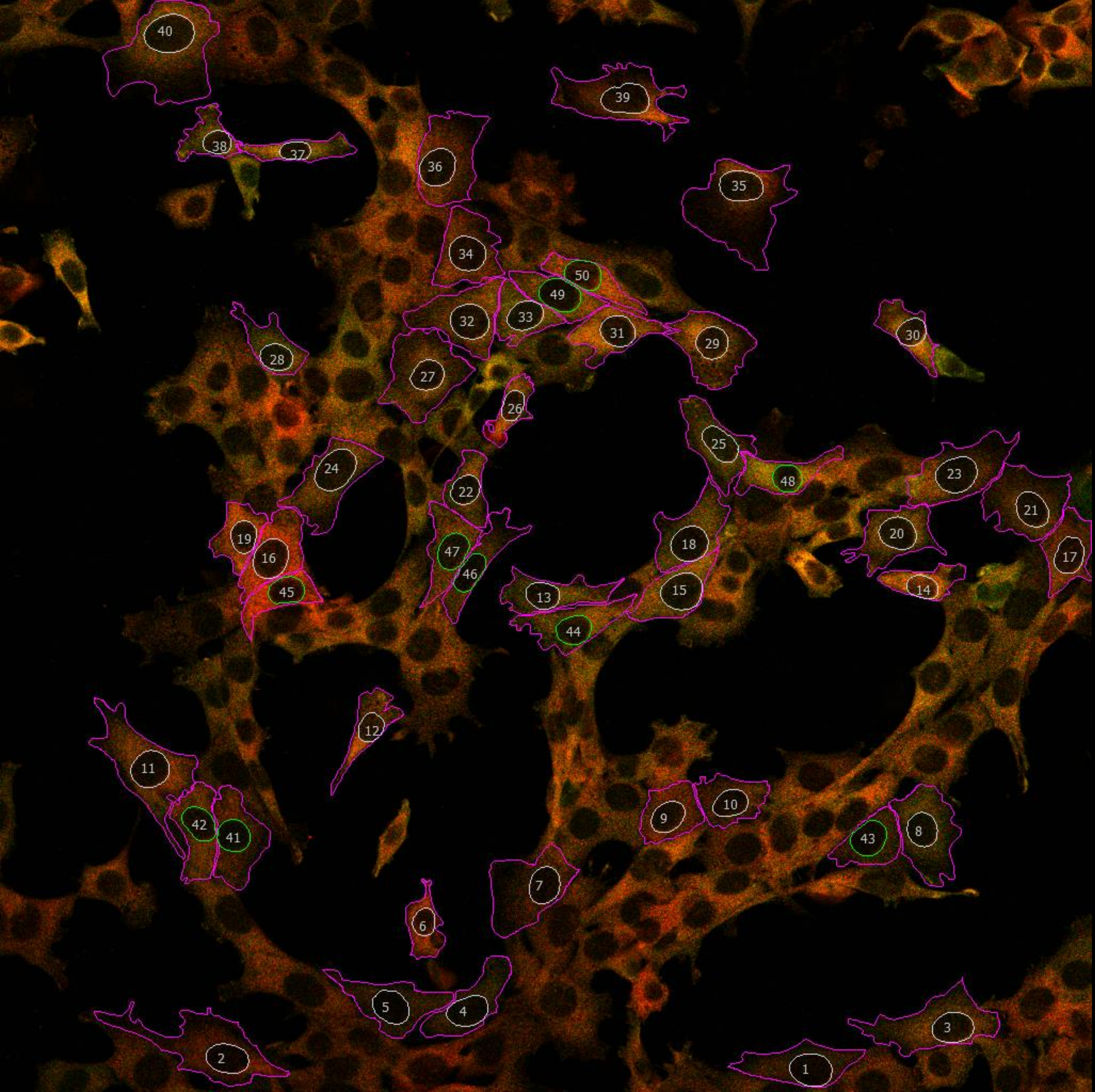

60 min TNFα 10 ng/ml

#	NFkB (nuc/total)	IkBa(total)/NFkB(total)
1	0.176	2.022
2	0.104	3.004
3	0.146	1.854
4	0.214	1.626
5	0.118	1.817
6	0.207	2.955
7	0.132	2.271
8	0.134	1.415
9	0.196	2.570
10	0.175	2.226
11	0.111	2.105
12	0.196	2.040
13	0.151	1.527
14	0.273	1.998
15	0.212	1.647
16	0.196	3.129
17	0.175	2.235
18	0.154	1.526
19	0.178	2.509
20	0.206	1.656
21	0.168	1.845
22	0.176	1.628
23	0.145	1.846
24	0.204	1.789
25	0.229	1.422
26	0.220	2.274
27	0.112	1.825
28	0.236	1.368
29	0.162	1.941
30	0.217	1.751
31	0.149	1.938
32	0.161	1.886
33	0.196	1.488
34	0.161	2.411
35	0.230	2.374
36	0.181	2.342
37	0.134	1.533
38	0.196	1.495
39	0.183	2.401
40	0.172	1.813
41	0.153	2.171
42	0.148	2.101
43	0.194	1.839
44	0.152	1.697
45	0.195	3.579
46	0.171	1.789
47	0.184	1.816
48	0.158	1.574
49	0.208	1.785
50	0.214	2.293

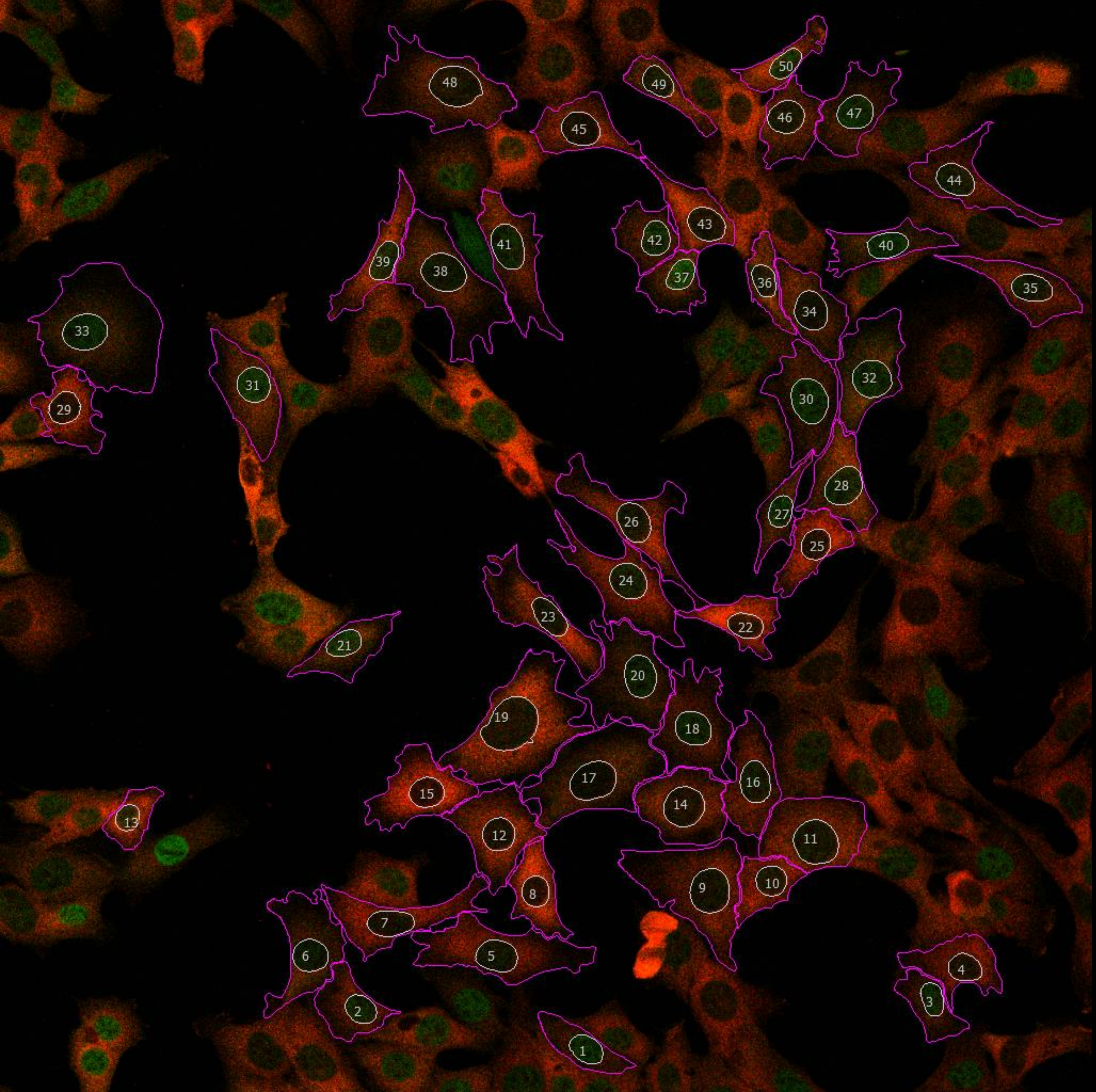

100 min TNFα 10 ng/ml

#	NFkB (nuc/total)	IkBa(total)/NFkB(total)
1	0.524	1.691
2	0.388	2.513
3	0.499	2.035
4	0.249	4.357
5	0.257	3.794
6	0.273	2.044
7	0.232	4.134
8	0.157	3.939
9	0.230	3.261
10	0.283	3.686
11	0.269	2.957
12	0.154	3.336
13	0.360	3.106
14	0.251	2.874
15	0.140	4.603
16	0.312	2.897
17	0.142	2.246
18	0.344	2.297
19	0.220	3.074
20	0.317	1.736
21	0.415	1.593
22	0.208	4.040
23	0.193	3.128
24	0.286	3.126
25	0.188	4.008
26	0.256	3.034
27	0.436	1.659
28	0.304	2.139
29	0.164	1.805
30	0.447	1.578
31	0.336	1.969
32	0.312	1.418
33	0.272	1.565
34	0.217	2.325
35	0.282	2.783
36	0.277	2.258
37	0.502	1.441
38	0.230	2.170
39	0.322	2.628
40	0.419	1.273
41	0.347	2.278
42	0.466	1.887
43	0.239	3.114
44	0.333	2.513
45	0.226	3.180
46	0.300	2.444
47	0.426	1.748
48	0.266	2.471
49	0.306	2.324
50	0.375	2.442

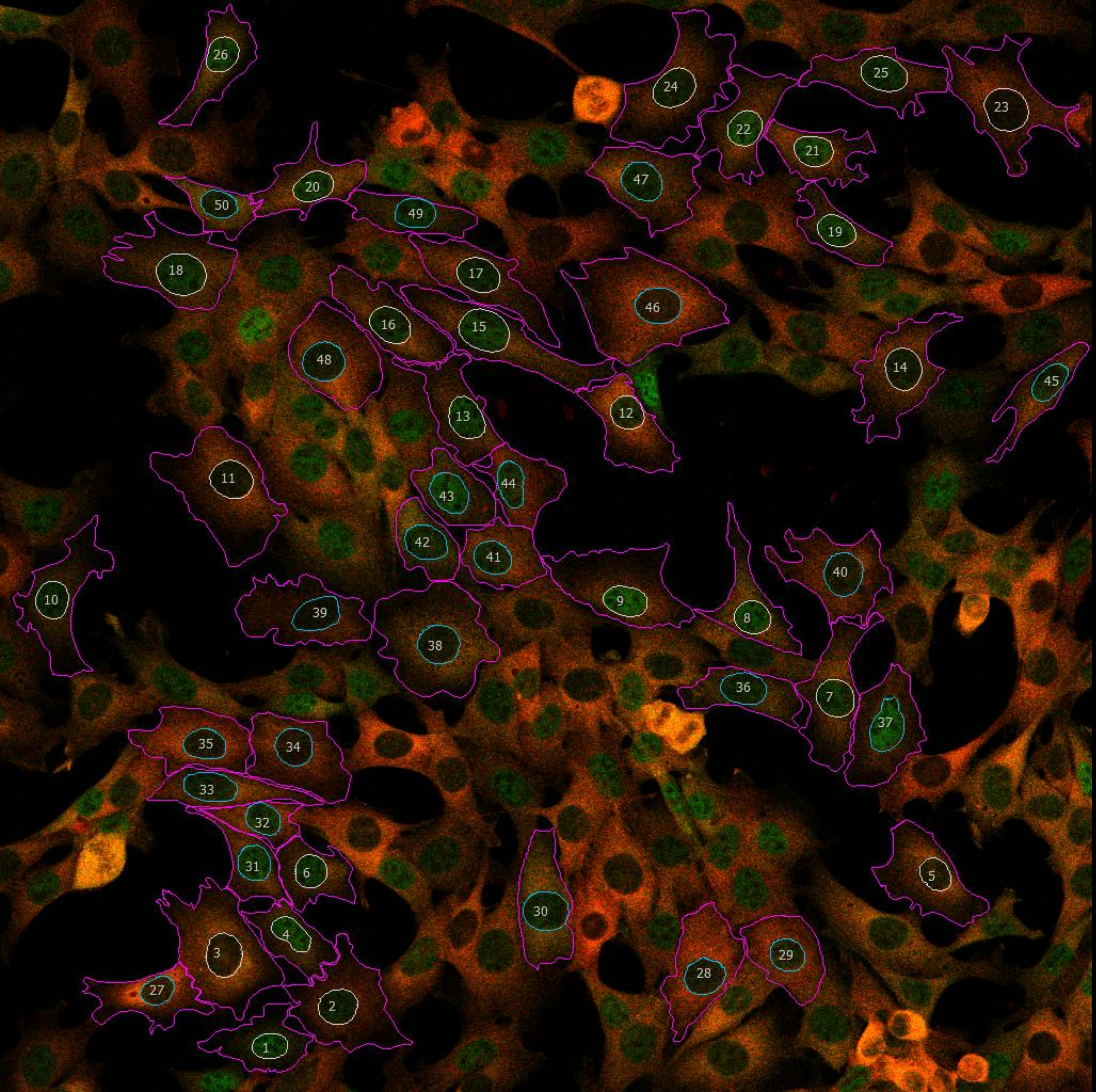

180 min TNFα 10 ng/ml

#	NFkB (nuc/total)	IkBa(total)/NFkB(total)
1	0.424	0.796
2	0.252	2.010
3	0.152	2.092
4	0.450	1.163
5	0.163	1.974
6	0.364	1.344
7	0.280	1.502
8	0.332	1.262
9	0.384	1.447
10	0.294	1.263
11	0.156	2.158
12	0.242	2.126
13	0.395	1.310
14	0.220	1.783
15	0.352	1.356
16	0.271	1.632
17	0.273	1.737
18	0.321	1.414
19	0.404	1.155
20	0.295	1.580
21	0.390	1.527
22	0.344	1.419
23	0.164	2.325
24	0.234	1.811
25	0.364	1.430
26	0.367	1.155
27	0.219	2.605
28	0.167	2.653
29	0.206	2.386
30	0.224	1.455
31	0.405	1.448
32	0.391	1.776
33	0.316	1.768
34	0.173	2.401
35	0.174	2.586
36	0.303	1.287
37	0.475	1.212
38	0.132	1.690
39	0.209	2.013
40	0.230	2.148
41	0.261	1.856
42	0.317	1.157
43	0.440	1.067
44	0.379	1.908
45	0.317	1.610
46	0.118	2.556
47	0.232	1.684
48	0.187	2.148
49	0.353	1.162
50	0.243	1.412
