# Supplementary material for: Importins promote high-frequency NF-κB oscillations increasing information channel capacity
Source: Biol Direct. 2016 Nov 11;11:61. doi: 10.1186/s13062-016-0164-z (PMC5106790; doi:10.1186/s13062-016-0164-z)
Supplement: Additional file 5: — Full confocal images with marked cells used for fluorescence quantification after 1 μg/ml LPS stimulation. Data table show raw nuclear to total NF-κB ratio and normalized total IκBα to total NF-κB ratio for each analysed cell (for quantification details see Methods). (PDF 1238 kb) [file 13062_2016_164_MOESM5_ESM.pdf]

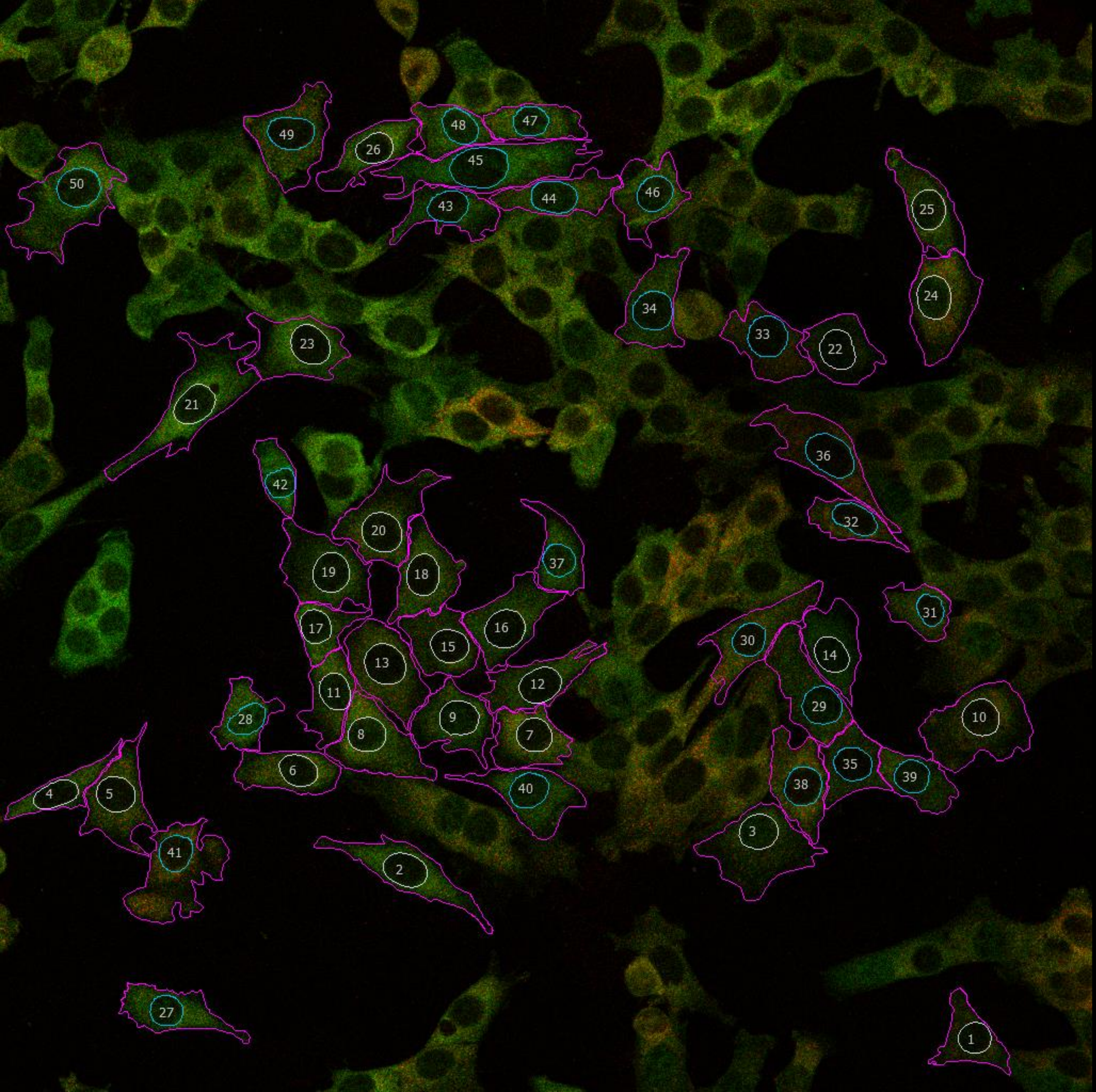

0 min - untreated

#	NFkB (nuc/total)	IkBa(total)/NFkB(total)
1	0.209	0.693
2	0.169	0.461
3	0.231	0.532
4	0.203	0.523
5	0.156	0.704
6	0.186	0.624
7	0.154	0.738
8	0.178	0.610
9	0.233	0.479
10	0.154	0.749
11	0.232	0.603
12	0.169	0.575
13	0.145	0.727
14	0.198	0.285
15	0.163	0.631
16	0.143	0.493
17	0.229	0.513
18	0.139	0.603
19	0.194	0.485
20	0.209	0.635
21	0.152	0.417
22	0.265	0.446
23	0.143	0.479
24	0.223	0.796
25	0.238	0.596
26	0.185	0.557
27	0.185	0.403
28	0.329	0.439
29	0.177	0.557
30	0.109	0.841
31	0.164	0.700
32	0.247	0.934
33	0.182	0.927
34	0.217	0.515
35	0.146	0.629
36	0.156	1.128
37	0.288	0.446
38	0.150	0.921
39	0.228	0.536
40	0.221	0.434
41	0.151	0.978
42	0.300	0.249
43	0.210	0.429
44	0.275	0.579
45	0.171	0.346
46	0.198	0.618
47	0.225	0.553
48	0.282	0.508
49	0.138	0.686
50	0.155	0.500

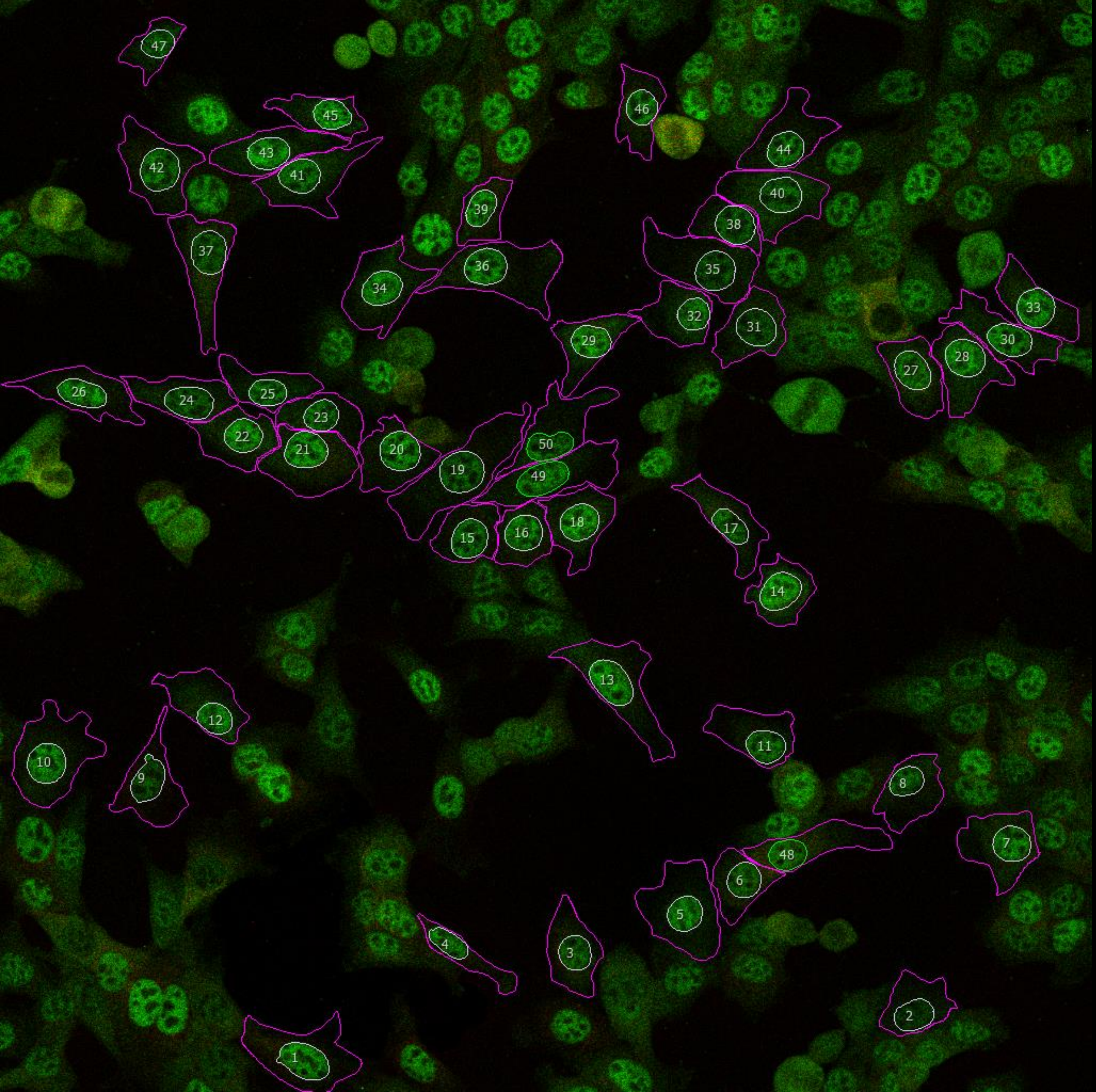

30 min LPS 1 μg/ml

#	NFkB (nuc/total)	IkBa(total)/NFkB(total)
1	0.760	0.233
2	0.553	0.168
3	0.603	0.267
4	0.738	0.253
5	0.598	0.071
6	0.621	0.290
7	0.557	0.208
8	0.544	0.191
9	0.795	0.394
10	0.643	0.159
11	0.729	0.165
12	0.564	0.144
13	0.667	0.136
14	0.713	0.154
15	0.733	0.165
16	0.708	0.203
17	0.546	0.246
18	0.686	0.190
19	0.586	0.203
20	0.738	0.167
21	0.618	0.240
22	0.608	0.180
23	0.601	0.178
24	0.612	0.167
25	0.602	0.097
26	0.749	0.161
27	0.581	0.244
28	0.602	0.156
29	0.653	0.177
30	0.594	0.170
31	0.487	0.209
32	0.560	0.157
33	0.688	0.163
34	0.578	0.152
35	0.513	0.212
36	0.603	0.180
37	0.504	0.295
38	0.735	0.158
39	0.705	0.248
40	0.394	0.184
41	0.558	0.144
42	0.662	0.219
43	0.610	0.136
44	0.643	0.194
45	0.697	0.099
46	0.656	0.252
47	0.638	0.134
48	0.553	0.345
49	0.678	0.150
50	0.604	0.140

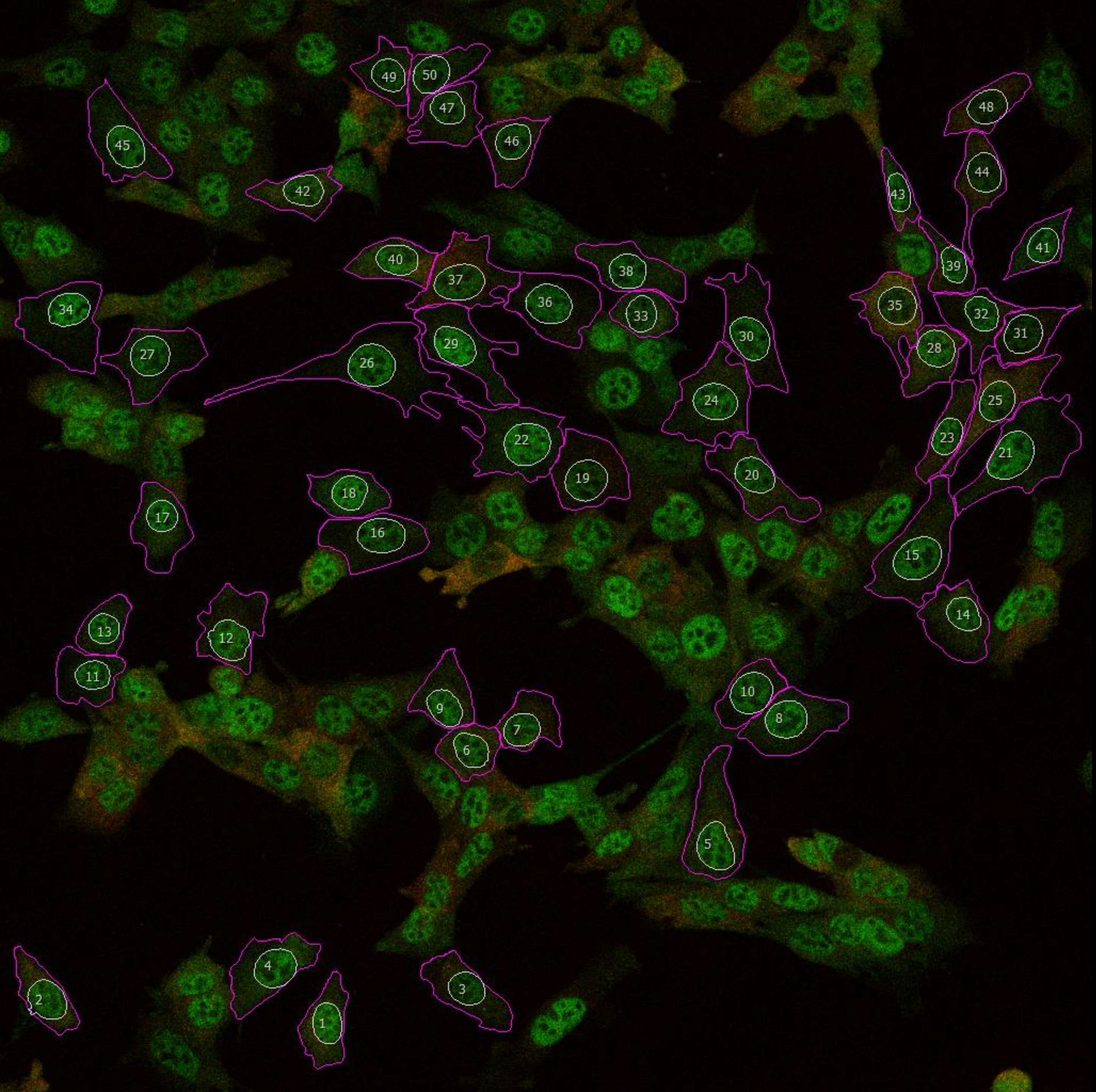

60 min LPS 1 μg/ml

#	NFkB (nuc/total)	IkBa(total)/NFkB(total)
1	0.589	0.411
2	0.652	0.438
3	0.502	0.472
4	0.502	0.272
5	0.499	0.383
6	0.523	0.502
7	0.653	0.532
8	0.561	0.268
9	0.590	0.368
10	0.752	0.276
11	0.577	0.193
12	0.649	0.379
13	0.591	0.229
14	0.464	0.409
15	0.539	0.334
16	0.579	0.325
17	0.453	0.261
18	0.573	0.185
19	0.592	0.562
20	0.435	0.419
21	0.594	0.189
22	0.619	0.226
23	0.551	0.454
24	0.487	0.345
25	0.398	0.706
26	0.640	0.251
27	0.543	0.233
28	0.564	0.490
29	0.634	0.198
30	0.618	0.294
31	0.629	0.526
32	0.550	0.366
33	0.454	0.314
34	0.622	0.162
35	0.356	0.808
36	0.673	0.249
37	0.517	0.710
38	0.452	0.295
39	0.579	0.328
40	0.512	0.506
41	0.594	0.164
42	0.529	0.489
43	0.592	0.271
44	0.484	0.591
45	0.547	0.212
46	0.550	0.377
47	0.463	0.401
48	0.512	0.752
49	0.523	0.308
50	0.530	0.317

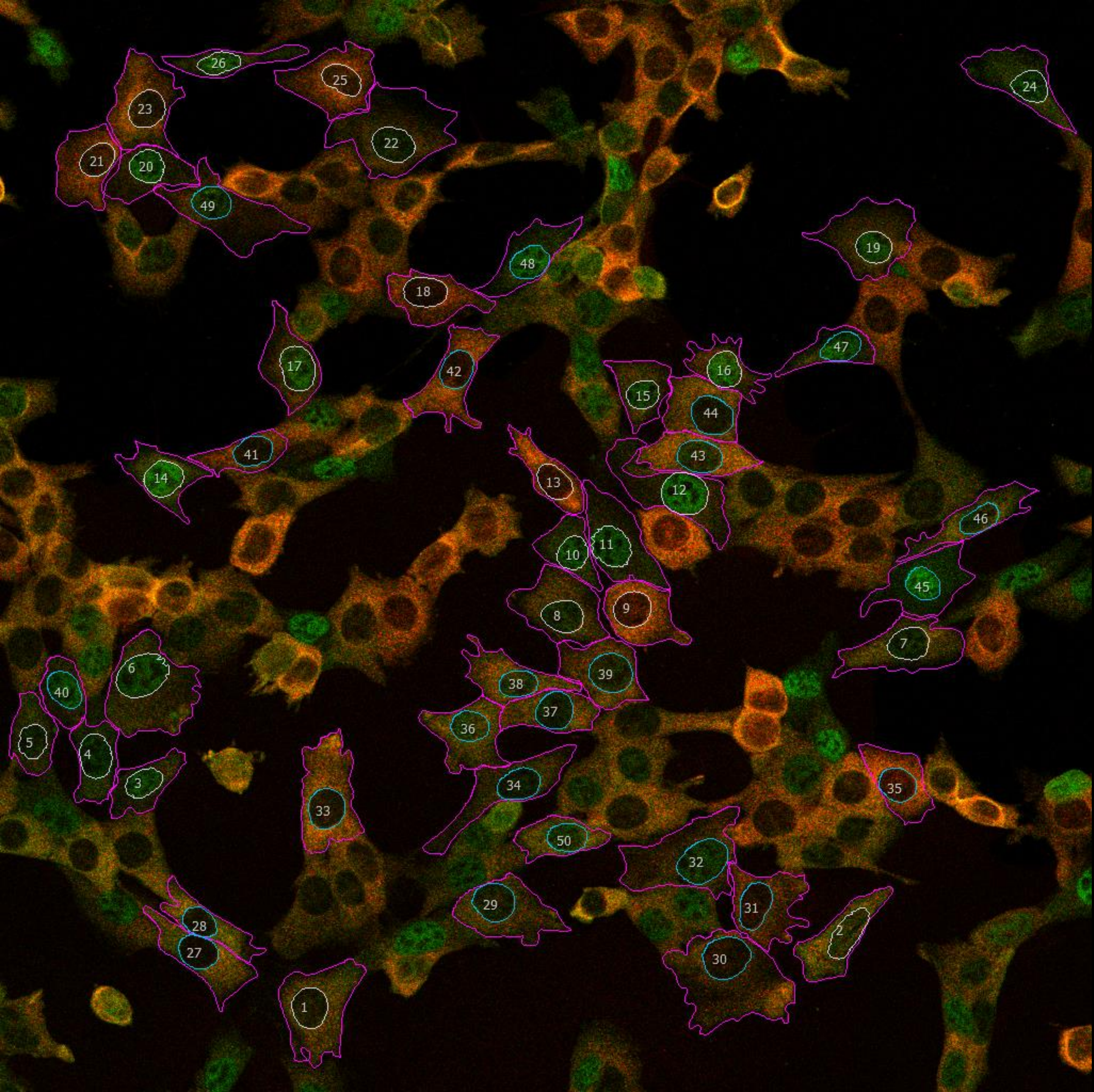

90 min LPS 1 μg/ml

#	NFkB (nuc/total)	IkBa(total)/NFkB(total)
1	0.167	1.215
2	0.297	1.114
3	0.472	0.610
4	0.420	0.484
5	0.259	0.765
6	0.346	0.797
7	0.238	1.030
8	0.252	1.165
9	0.171	2.125
10	0.458	0.561
11	0.445	0.605
12	0.573	0.625
13	0.190	2.269
14	0.585	0.511
15	0.367	0.903
16	0.645	0.748
17	0.548	0.845
18	0.172	2.094
19	0.331	0.981
20	0.499	0.674
21	0.213	1.975
22	0.226	1.058
23	0.175	1.682
24	0.309	0.496
25	0.203	2.012
26	0.565	0.515
27	0.113	1.217
28	0.161	1.153
29	0.240	1.137
30	0.124	1.529
31	0.176	1.849
32	0.354	1.070
33	0.129	1.690
34	0.225	1.040
35	0.193	2.331
36	0.257	1.133
37	0.239	1.241
38	0.231	1.319
39	0.305	1.367
40	0.579	0.565
41	0.246	1.747
42	0.123	1.976
43	0.237	1.501
44	0.193	1.363
45	0.435	0.274
46	0.253	0.948
47	0.584	0.548
48	0.517	0.410
49	0.330	1.150
50	0.321	0.982

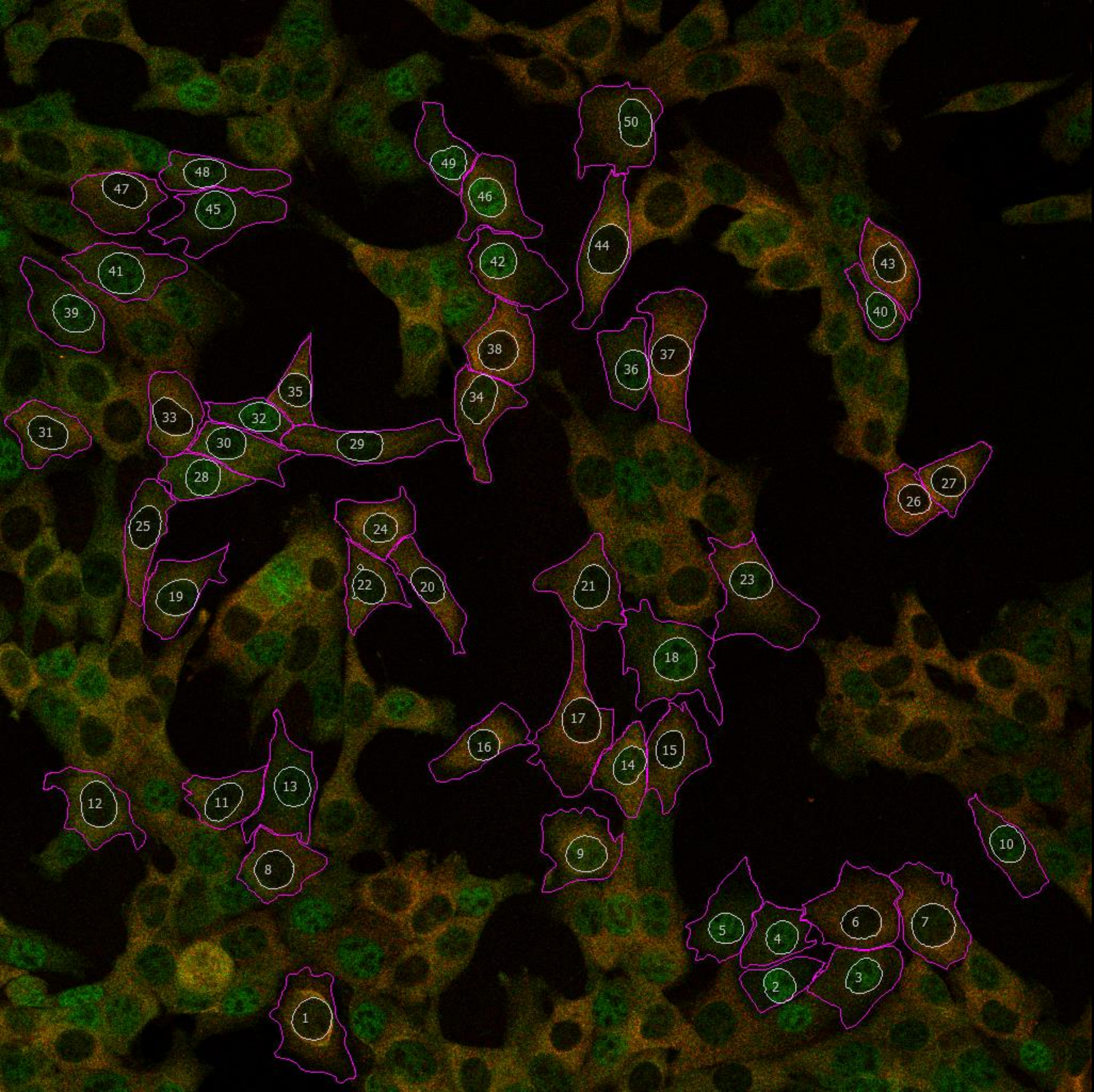

120 min LPS 1 μg/ml

#	NFkB (nuc/total)	IkBa(total)/NFkB(total)
1	0.276	1.252
2	0.609	0.277
3	0.347	0.739
4	0.432	0.547
5	0.439	0.461
6	0.155	1.407
7	0.271	1.249
8	0.218	1.086
9	0.347	0.982
10	0.432	0.701
11	0.240	0.895
12	0.236	0.890
13	0.249	0.615
14	0.289	1.027
15	0.152	1.110
16	0.218	1.184
17	0.192	1.496
18	0.349	0.638
19	0.293	0.920
20	0.205	1.196
21	0.205	1.109
22	0.244	1.018
23	0.258	1.089
24	0.259	1.245
25	0.160	1.007
26	0.198	1.897
27	0.196	1.447
28	0.356	0.545
29	0.218	0.931
30	0.237	0.727
31	0.208	1.021
32	0.546	0.410
33	0.171	1.225
34	0.336	1.212
35	0.296	1.116
36	0.404	0.634
37	0.157	1.500
38	0.214	1.523
39	0.350	0.537
40	0.443	0.608
41	0.400	0.612
42	0.437	0.744
43	0.224	1.466
44	0.209	1.111
45	0.397	0.546
46	0.452	0.731
47	0.214	1.015
48	0.321	0.694
49	0.482	0.432
50	0.299	1.018
