# Supplementary material for: Importins promote high-frequency NF-κB oscillations increasing information channel capacity
Source: Biol Direct. 2016 Nov 11;11:61. doi: 10.1186/s13062-016-0164-z (PMC5106790; doi:10.1186/s13062-016-0164-z)
Supplement: Additional file 6: — Full confocal images with marked nuclei used for NF-κB fluorescence quantification after 5 μg/ml CHX + 1 μg/ml LPS stimulation. CHX stimulation starts 60 min before LPS. (PDF 1087 kb) [file 13062_2016_164_MOESM6_ESM.pdf]

Supplementary Figure to **Figure 1D**

MEF WT

60 min CHX 5 µg/ml

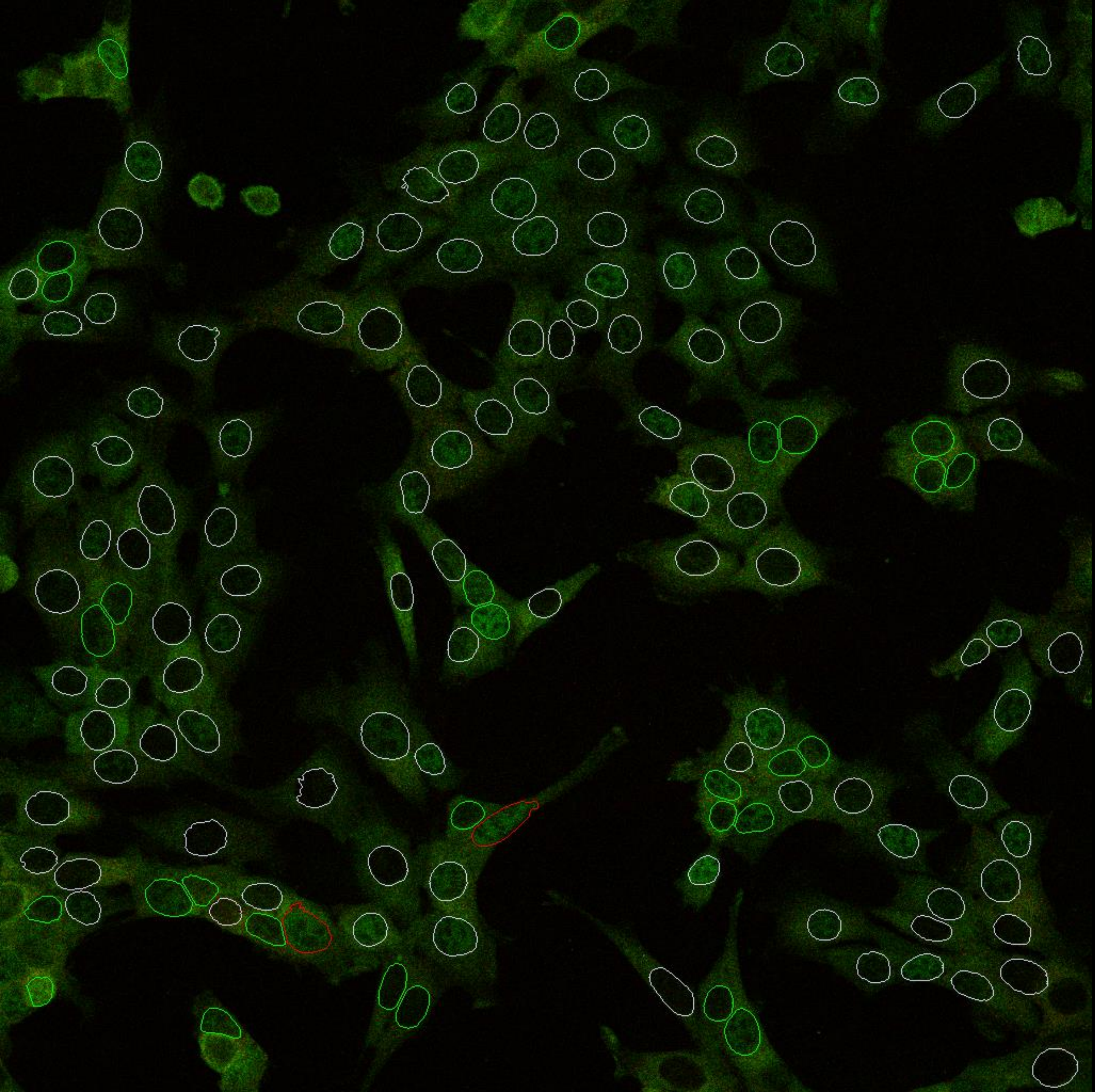

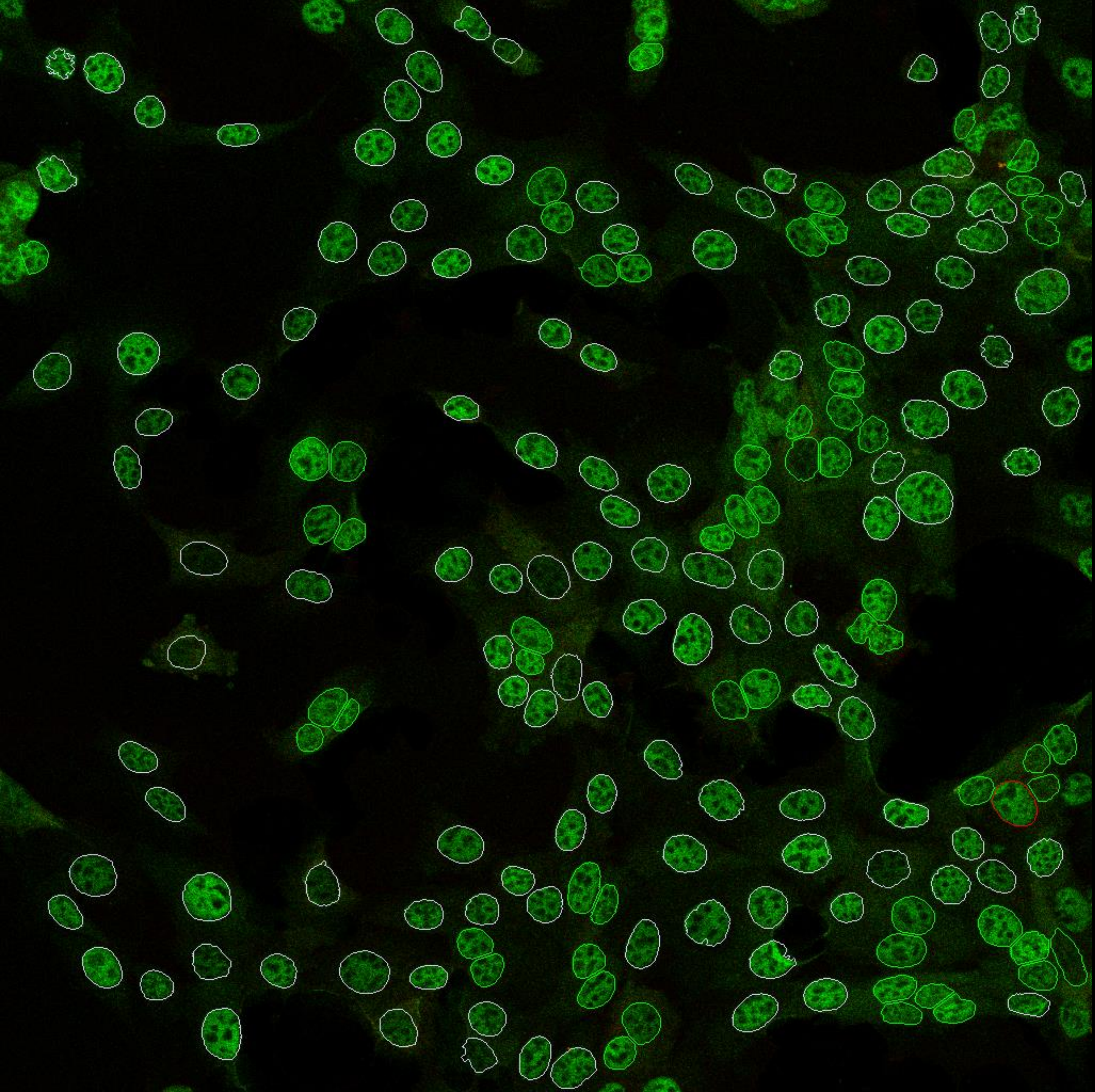

Supplementary Figure to **Figure 1D**

MEF WT

90 min CHX 5 µg/ml +
30 min LPS 1 µg/ml

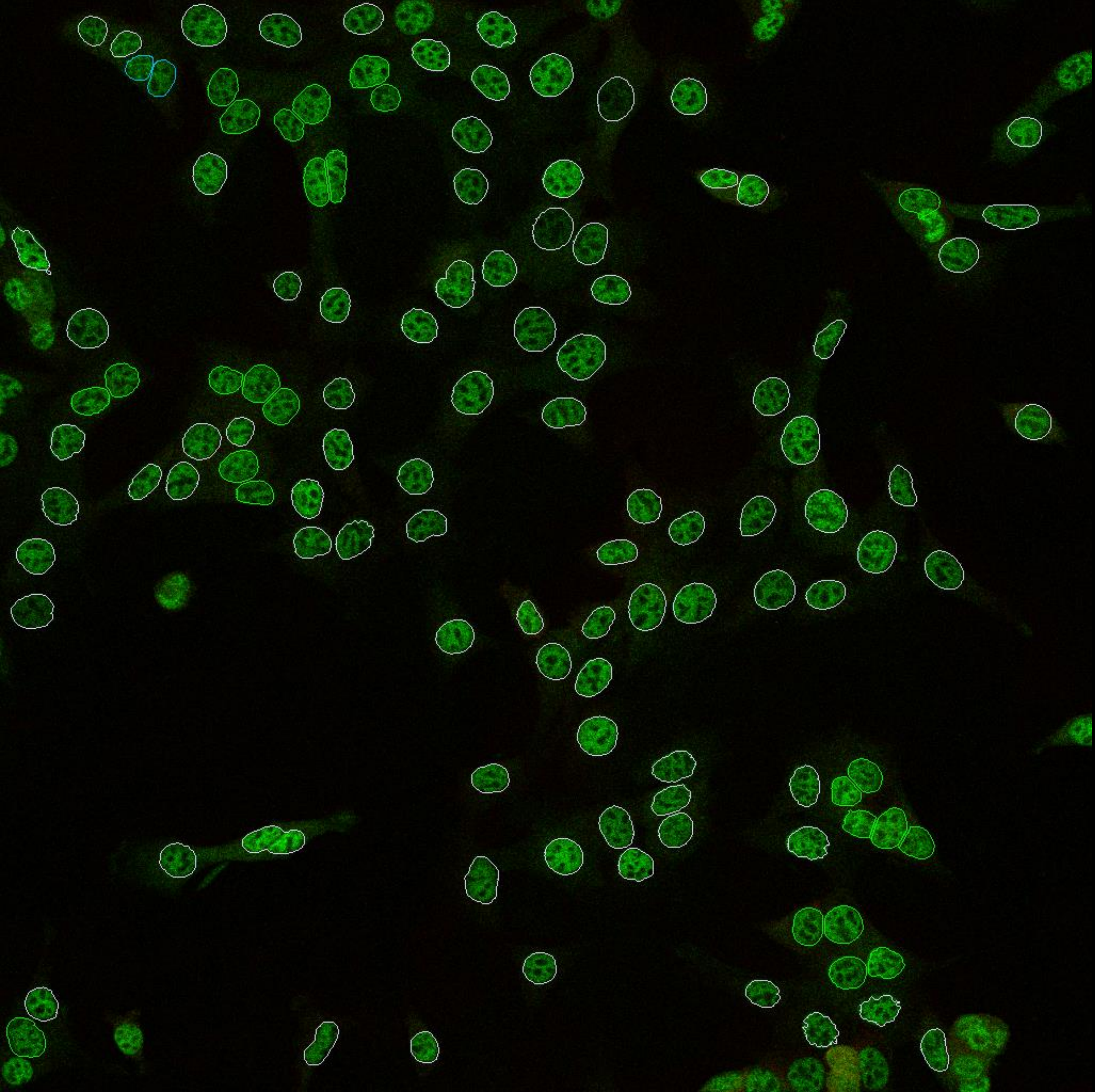

Supplementary Figure to **Figure 1D**

MEF WT

120 min CHX 5 µg/ml +
60 min LPS 1 µg/ml

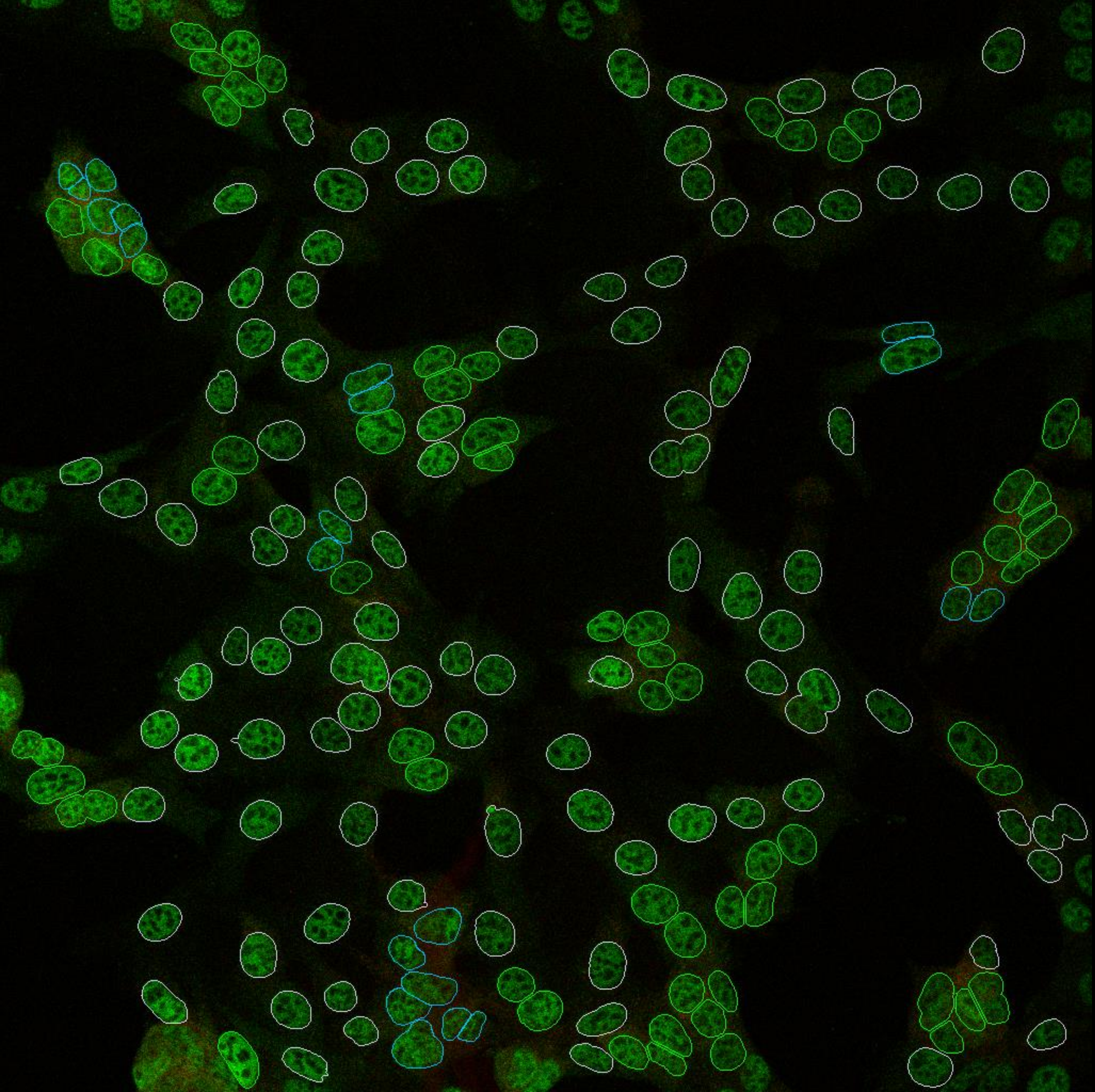

Supplementary Figure to **Figure 1D**

MEF WT

150 min CHX 5 µg/ml +
90 min LPS 1 µg/ml

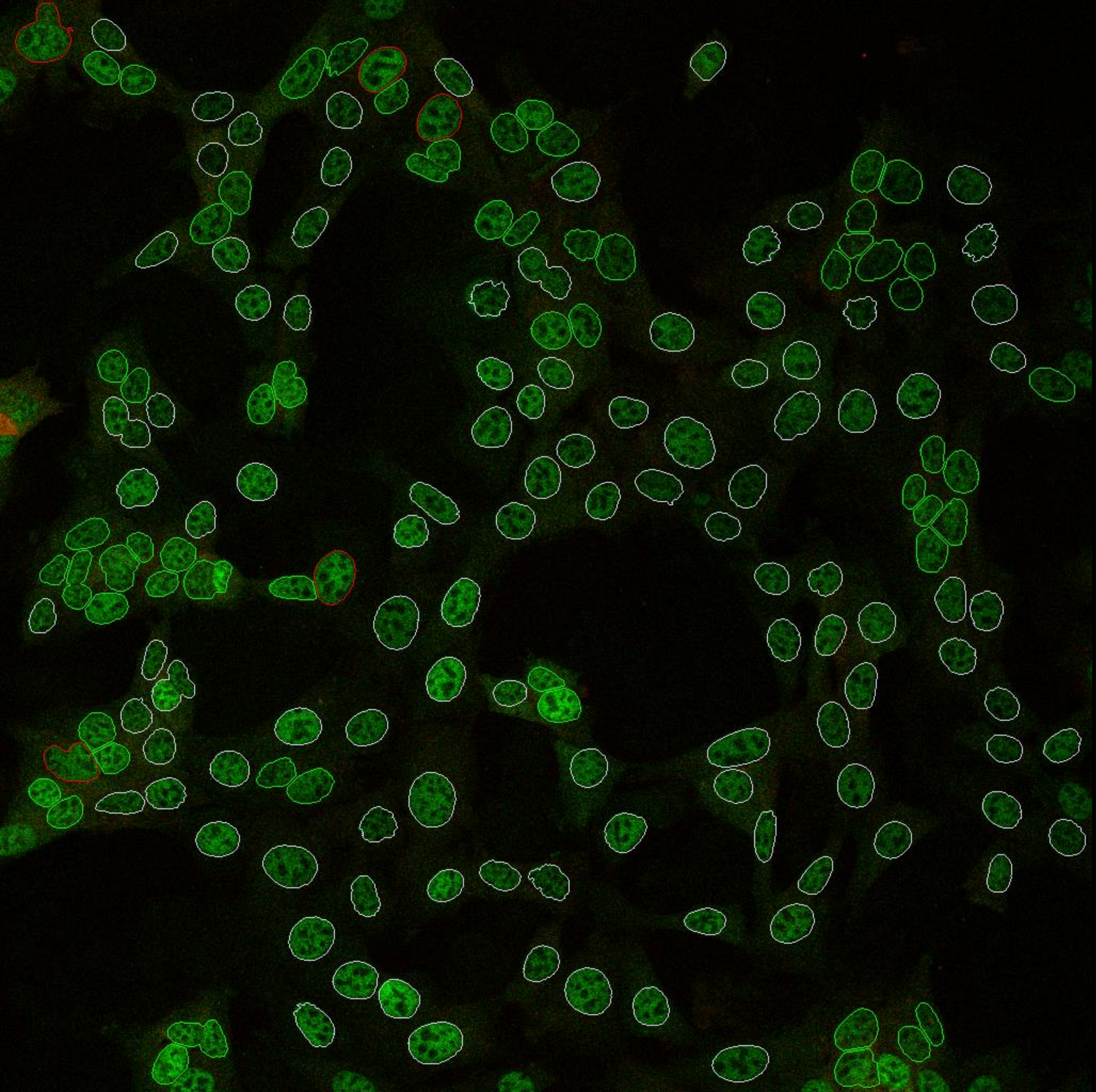

Supplementary Figure to **Figure 1D**

MEF WT

180 min CHX 5 µg/ml +
120 min LPS 1 µg/ml

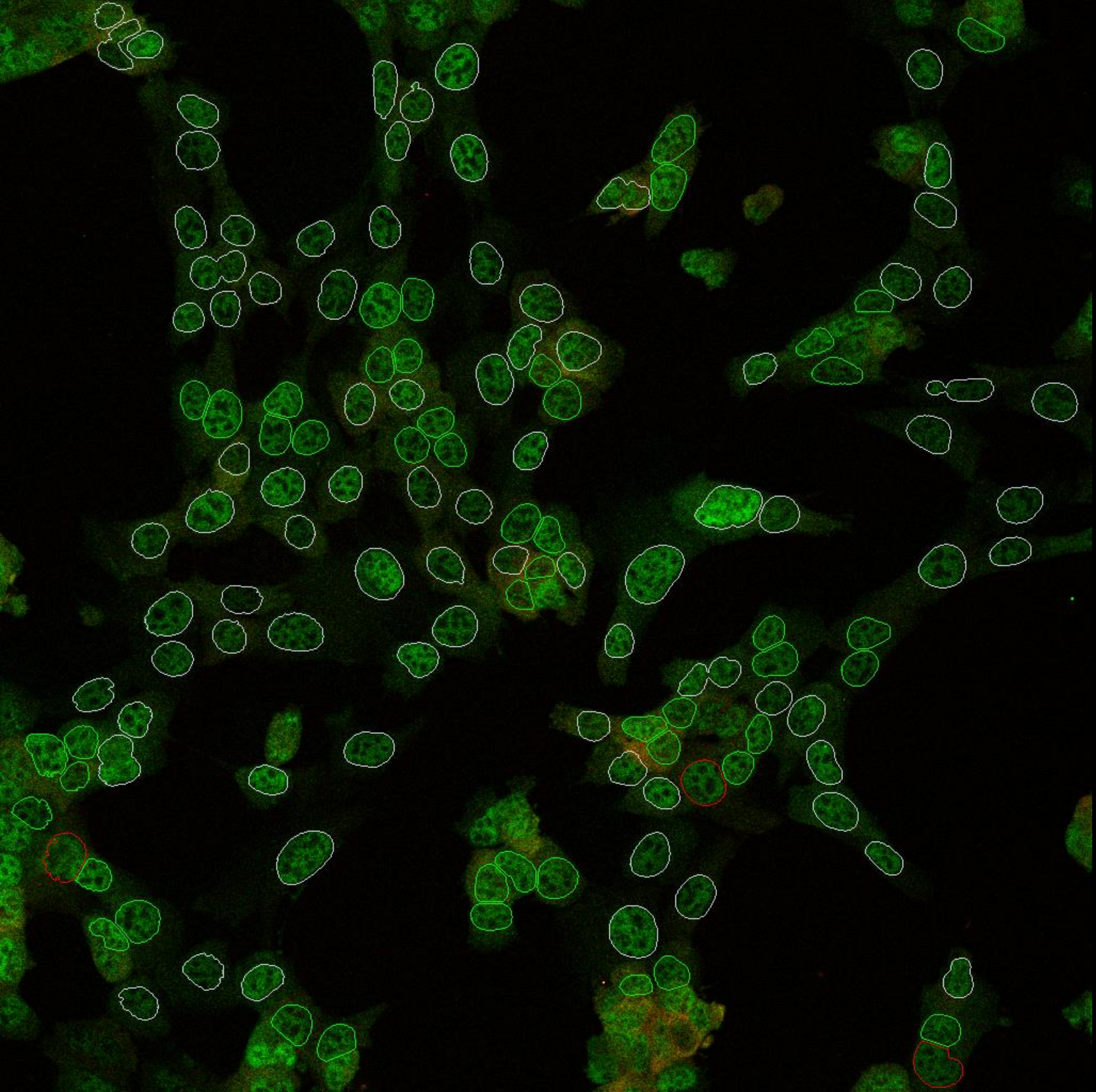

Supplementary Figure to **Figure 1D**

MEF WT

300 min CHX 5 µg/ml +
240 min LPS 1 µg/ml
