# Supplementary material for: Importins promote high-frequency NF-κB oscillations increasing information channel capacity
Source: Biol Direct. 2016 Nov 11;11:61. doi: 10.1186/s13062-016-0164-z (PMC5106790; doi:10.1186/s13062-016-0164-z)
Supplement: Additional file 7: — Full confocal images of cells stimulated with 10 ng/ml TNFα for 0, 5, 10, 15, 20, and 30 min. (PDF 733 kb) [file 13062_2016_164_MOESM7_ESM.pdf]

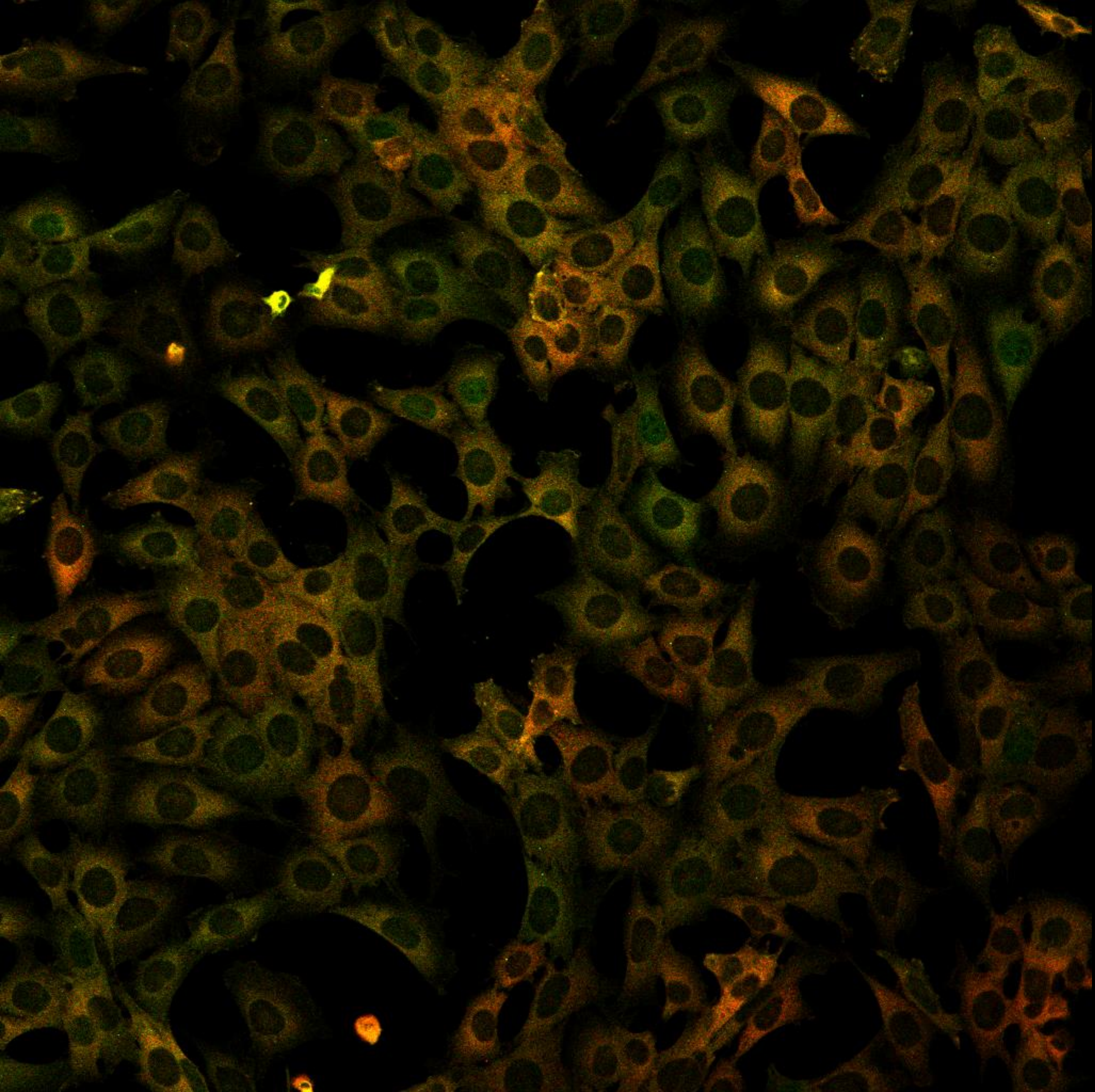

Supplementary Figure to **Figure 1A**

MEF WT

0 min (untreated)

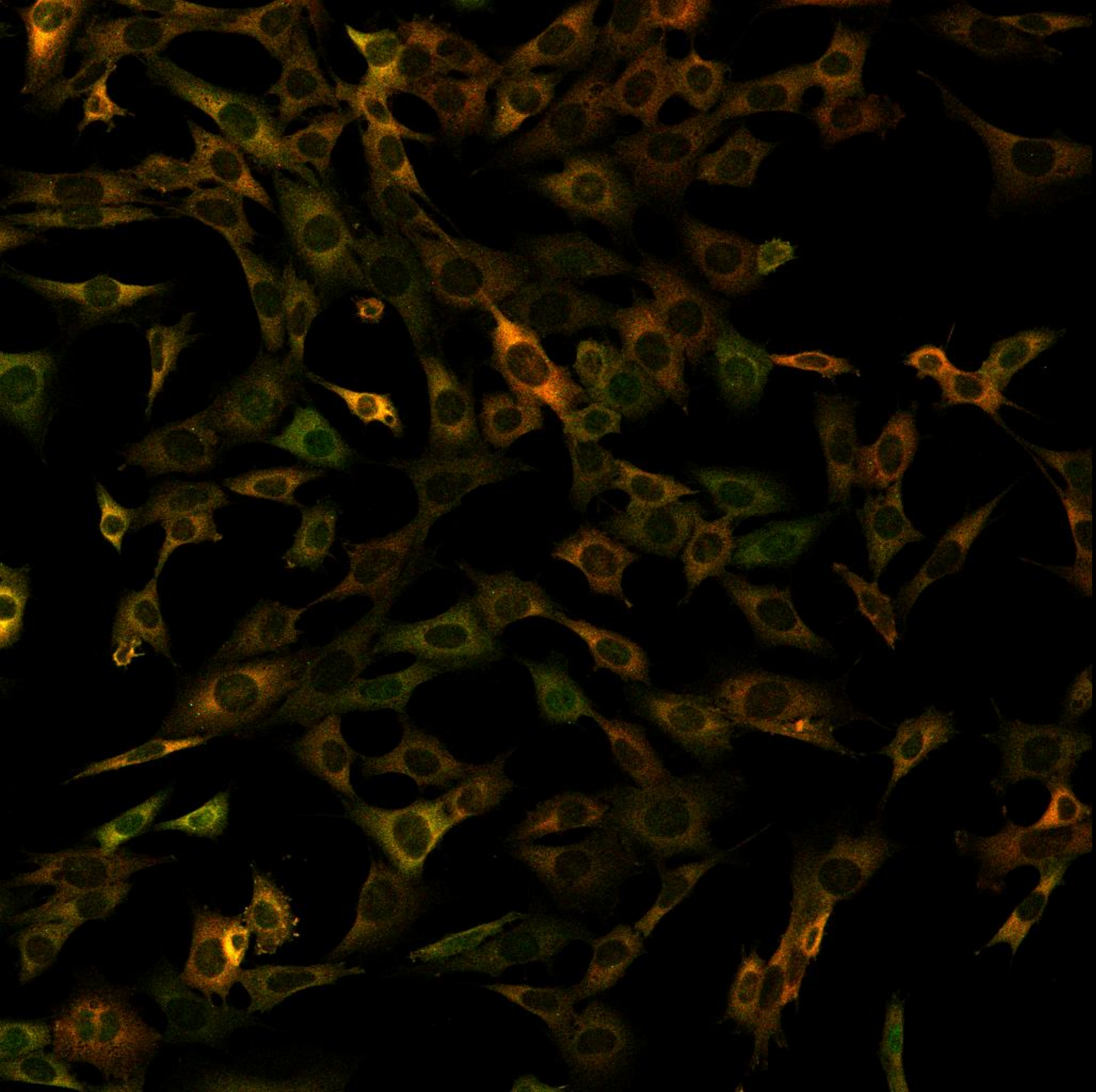

Supplementary Figure to **Figure 1A**

MEF WT

5 min TNFα 10 ng/ml

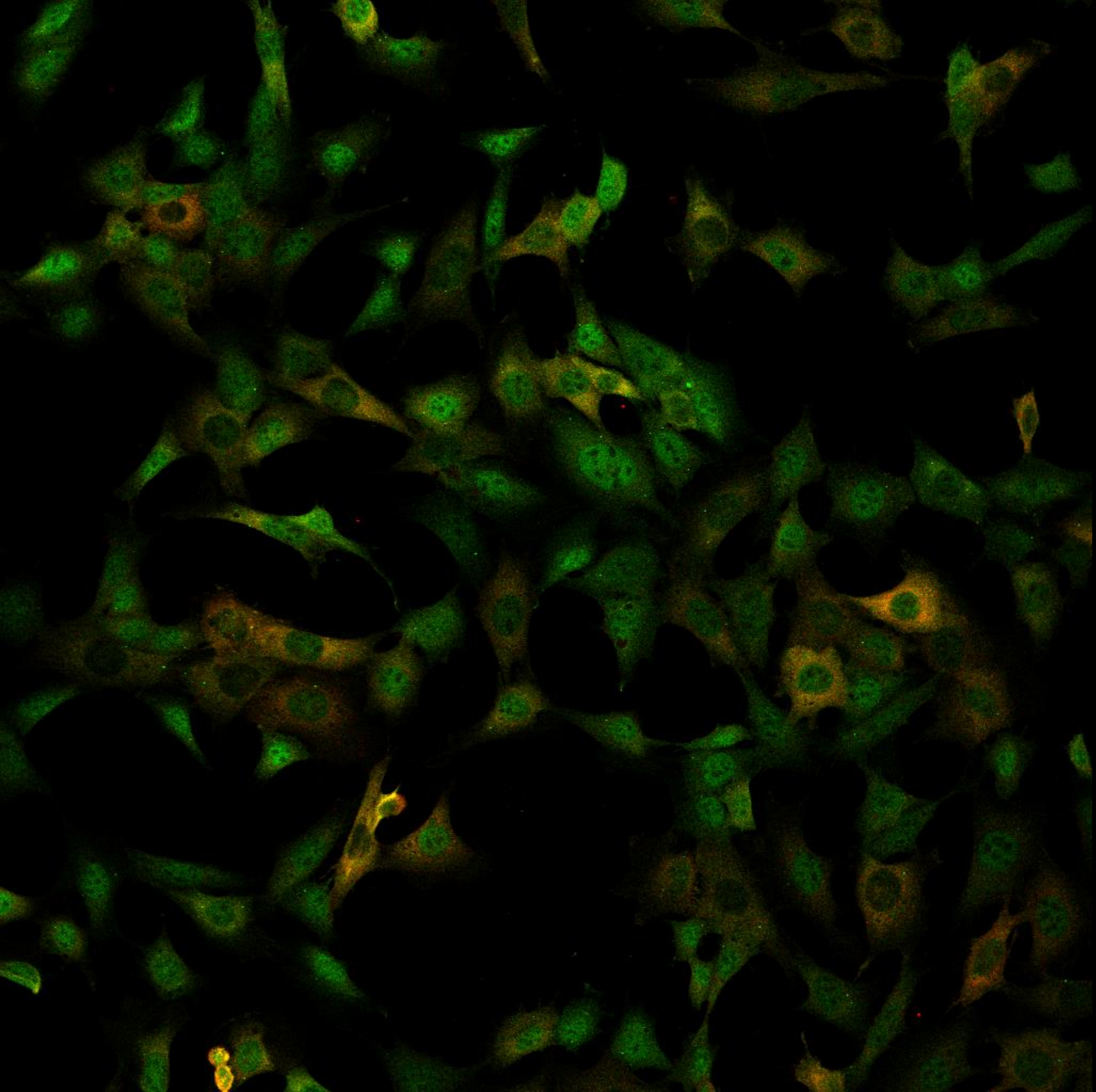

Supplementary Figure to **Figure 1A**

MEF WT

10 min TNFα 10 ng/ml

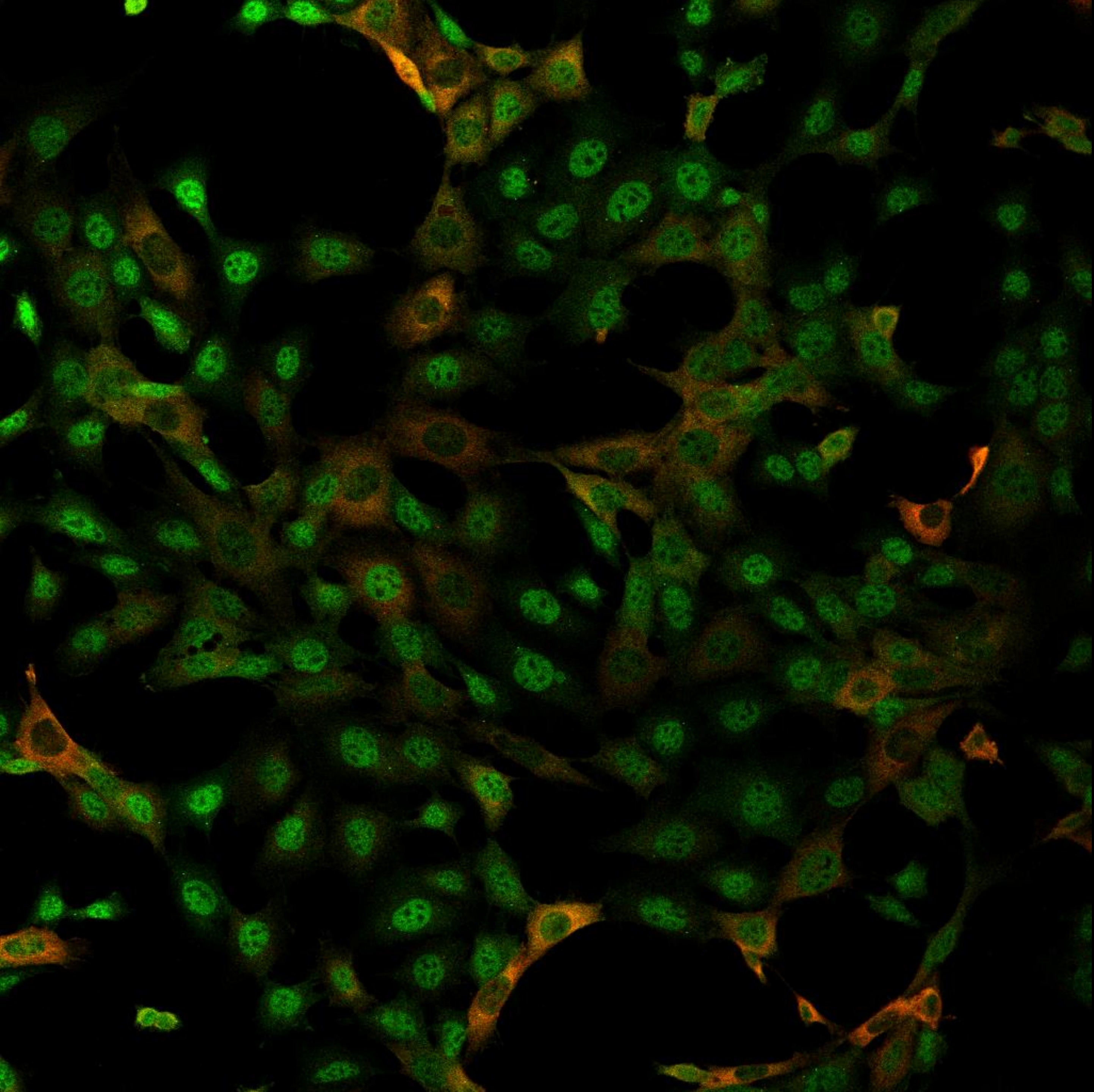

Supplementary Figure to **Figure 1A**

MEF WT

15 min TNFα 10 ng/ml

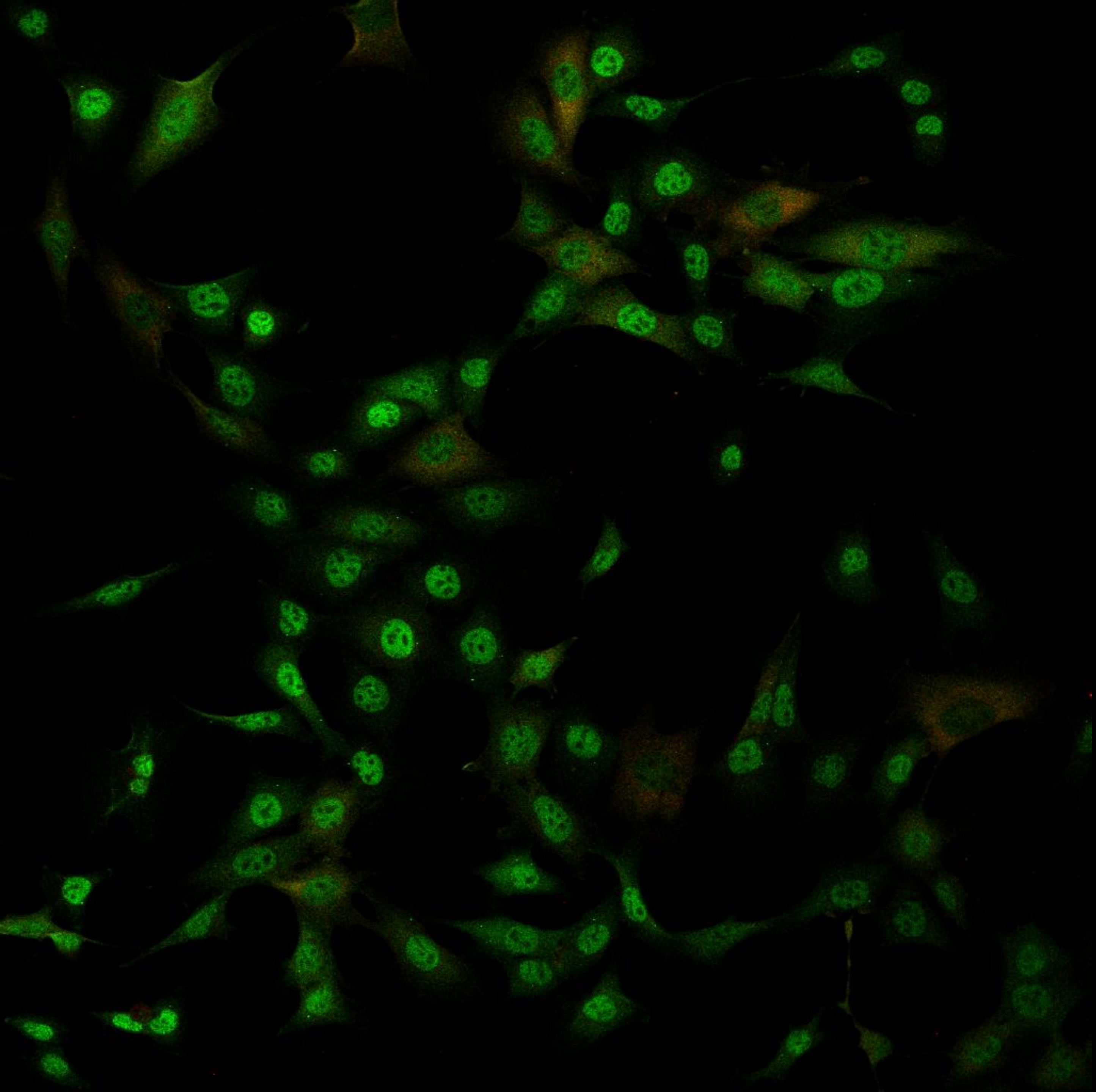

Supplementary Figure to **Figure 1A**

MEF WT

20 min TNFα 10 ng/ml

Supplementary Figure to **Figure 1A**

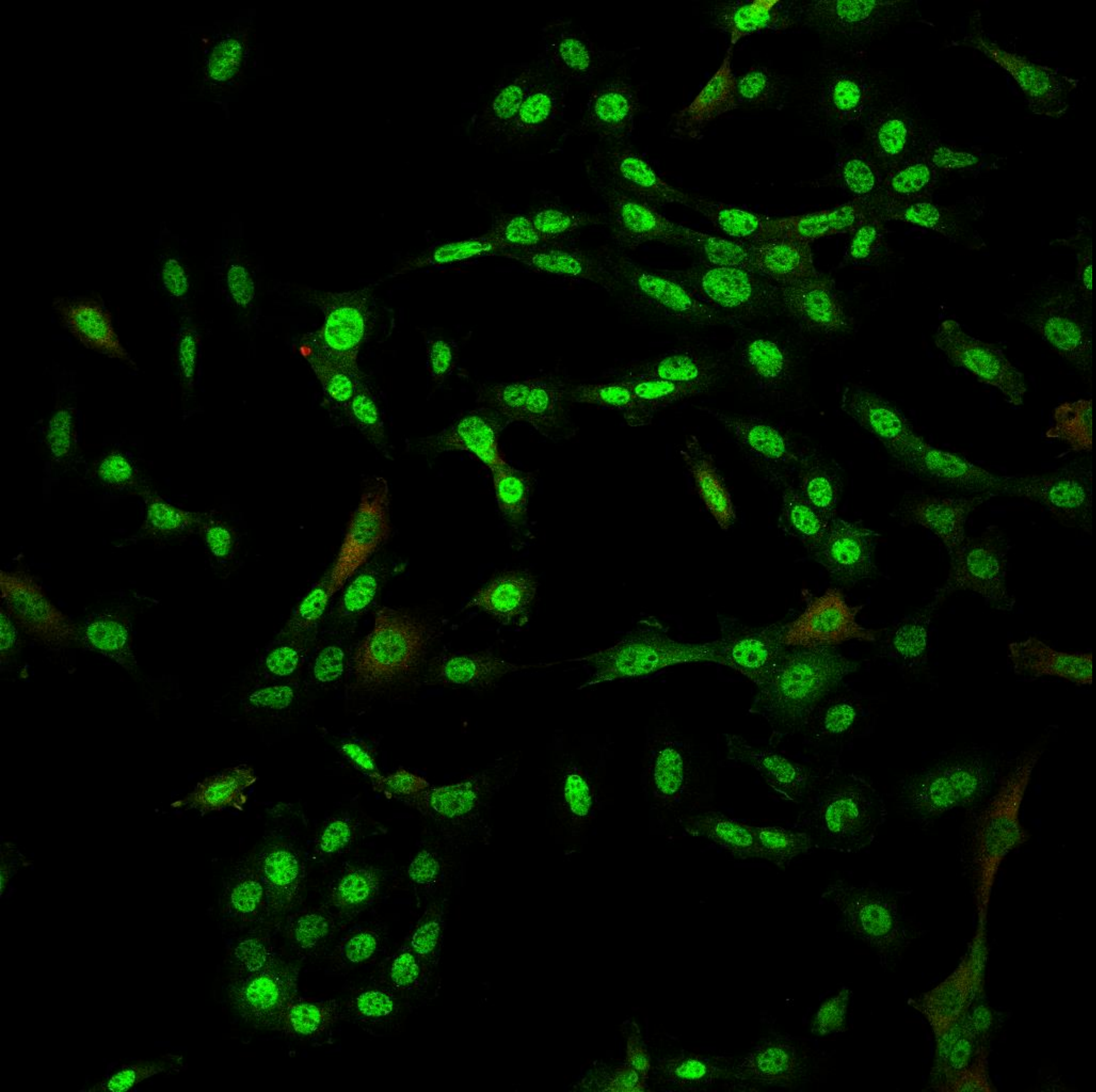

MEF WT

30 min TNFα 10 ng/ml
